# Mechanochemical Dehalogenative Deuteration of Alkyl Halides Through Piezoelectric Catalysis Initiated by a Single‐Electron Oxidation Strategy

**DOI:** 10.1002/advs.202515449

**Published:** 2025-08-30

**Authors:** Ruiling Qu, Ruoxuan Liu, Xiaochun He, Xuemei Zhang, Zhong Lian

**Affiliations:** ^1^ Department of Dermatology State Key Laboratory of Biotherapy and Cancer Center West China Hospital Sichuan University Chengdu 610041 P. R. China

**Keywords:** ball milling, deuterodehalogenation, mechanochemistry, piezoelectric catalysis, single‐electron oxidation

## Abstract

Deuterium labeling is extensively utilized across various scientific disciplines. The dehalogenative deuteration of organic halides offers a promising approach for achieving deuterium labeling. However, existing methods for dehalogenative deuteration primarily focus on sp^2^‐hybridized aryl halides, while sp^3^‐hybridized alkyl halides, especially bromides and chlorides, exhibit low reactivity and pose significant challenges for reduction. This limitation hampers the development of deuteration methodologies. In this study, a robust and versatile mechanochemical strategy is introduced for deuterating both activated and unactivated alkyl halides (X = Cl, Br, I), employing D_2_O as an economical deuterium source and electron donor, catalyzed by a piezoelectric material. Importantly, unlike previously mechanochemical piezoelectric catalysis reactions that are typically initiated by a single‐electron reduction process, this transformation is triggered by a single‐electron oxidation pathway. Employing this innovative technique, a variety of organic halides are successfully converted, including primary, secondary, and tertiary alkyl halides, into deuterated products with good yields and high deuterium incorporation, using only a chemically equivalent amount of D_2_O. The practical application of this green and efficient methodology is further demonstrated by late‐stage deuteration on drug molecule analogs, underscoring its potential utility in pharmaceutical development.

## Graphical abstract

A mechanochemical method is developed using D_2_O and a piezoelectric catalyst to deuterate both activated and unactivated alkyl halides (Cl, Br, I) efficiently. Unlike typical mechanochemical reactions, this one starts with single‐electron oxidation. This green technique successfully deuterates various alkyl halides, including primary, secondary, and tertiary ones, with high yields and deuteration, making it valuable for pharmaceutical development.

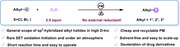

## 1 Introduction

**Figure 1:**
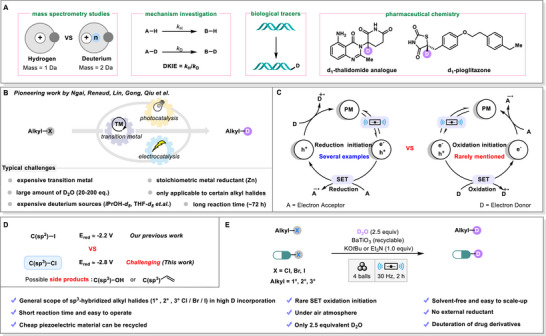
A) Applications of deuterium labeling; B) State‐of‐the‐art in deuteration of alkyl halides; C) Modes of mechanochemical piezoelectric catalysis; D) Challenges in deuterodehalogenation of alkyl chlorides; E) Summary of our approach.

Since its discovery in 1931,^[^ ^1^ ^]^ deuterium has become an indispensable element across various fields, including mass spectrometry,^[^ ^2^ ^]^ mechanistic studies,^[^ ^3^ ^]^ biological tracing,^[^ ^4^ ^]^ and pharmaceutical chemistry (**Figure** **1**A).^[^ ^5^ ^]^ It is particularly critical in medicinal chemistry, where deuterium labeling plays a vital role in drug development by modifying the absorption, distribution, metabolism, and excretion (ADME) properties of drugs through subtle chemical changes. In 2017, the FDA approved the first deuterated drug, Austedo (deutetrabenazine), for the treatment of chorea associated with Huntington's disease. This milestone has not only stimulated interest in deuterated drug research within the pharmaceutical industry but has also increased the demand for practical and effective deuteration strategies.

Current research on deuteration has predominantly concentrated on the direct dehalogenative deuteration of organic halides, especially sp^2^‐hybridized aryl halides (For selected examples, see).^[^ ^6^ ^]^ However, the deuteration of unactivated sp^3^‐hybridized alkyl halides, particularly those with highly negative reduction potentials (e.g., E_red_ [(*n*PrCl)/(*n*PrCl)^·‒^] = −2.8 V vs SCE),^[^ ^7^ ^]^ remains inadequately explored. Traditional methods primarily depend on transition metal catalysis,^[^ ^8^ ^]^ but several innovative strategies have recently been introduced to mitigate this reliance (Figure 1B).^[^ ^9^ ^]^ Despite these achievements, these new approaches still face several challenges that restrict their applicability (Figure 1B). These challenges include: 1) only applicable to a particular type of alkyl halide (e.g., alkyl iodides^[^ ^9a,b^ ^]^ or benzyl chlorides^[^ ^9c^ ^]^); 2) the requirement for stoichiometric amounts of reducing metals (e.g., Zn^[^ ^9d^ ^]^); 3) the demand for expensive deuterium sources (e.g., *i*PrOH‐*d_8_*,^[^ ^8a^ ^]^ THF‐*d_8_* et.al^[^ ^8c,d^ ^]^) or the need for substantial quantities of D_2_O (20–200 equiv.);^[^ ^9e–j^ ^]^ 4) prolonged reaction times (e.g., up to 72 h).^[^ ^9f^ ^]^ Therefore, there is a pressing need to develop a sustainable, efficient, and cost‐effective universal strategy for the dehalogenative deuteration of alkyl halides.

In recent years, the research field of mechanochemistry has been rapidly advanced (For selected reviews on organic synthesis using mechanochemistry, see; For selected examples on organic synthesis using mechanochemistry, see),^[^ ^10^, ^11^ ^]^ utilizing piezoelectric materials to facilitate charge transfer between reactants under mechanical stimulation, thereby offering a novel pathway for compound synthesis (For selected reviews on organic synthesis using piezoelectric materials under mechanochemistry, see).^[^ ^12^ ^]^ In 2019, Ito successfully employed piezoelectric BaTiO_3_ for the first time in the redox activation‐assisted C−H arylation and borylation reactions, thereby pioneering the application of piezoelectric materials in mechanochemical synthesis.^[^ ^13^ ^]^ Almost simultaneously, Bolm's group demonstrated that piezoelectric materials could facilitate the reduction of a Cu (II) precatalyst into the active Cu (I) species in solvent‐free atom transfer radical cyclization (ATRC) reactions.^[^ ^14^ ^]^ Recently, several research groups, including ours, have successfully utilized piezoelectric materials to perform various transformations through mechanochemical strategies.^[^ ^15^ ^]^ However, in these studies, chemical reactions are normally initiated by a single‐electron reduction process, while reactions initiated by a single‐electron oxidation process have rarely been reported (Figure 1C) (There is only one report of single‐electron oxidation initiation).^[^ ^16^ ^]^

We recently reported the mechanochemical dehalogenative deuteration of aryl iodides,^[^ ^16^ ^]^ and we were interested in extending this approach to the deuteration of C(sp^3^)‐X bonds. However, the mechanochemical conversion of unactivated alkyl halides, especially alkyl chlorides with highly negative reduction potential (E_red_ ≈ −2.8 V), poses significant challenges due to their higher reduction potential compared to aryl iodides (E_red_ ≈ −2.2 V) (Figure 1D). Herein, we present a universal strategy for the mechanochemical deuteration of both activated and unactivated alkyl halides, triggered by a single‐electron oxidation process facilitated by charge exchange with piezoelectric materials. In this process, inexpensive D_2_O serves as both the deuterium source and the electron donor. This method operates effectively under ambient conditions with stoichiometric amounts of D_2_O, achieving high levels of deuteration for primary, secondary, and tertiary alkyl halides within a short time. Notably, the system suppresses common side reactions such as alcohol or alkene formation induced by strong bases, ensuring excellent deuterium incorporation. Additionally, the late‐stage deuteration modification of drug‐like molecules and the recyclability of piezoelectric materials highlight the practicality and economic viability of this approach (Figure 1E).

## 2 Results and Discussion

**Figure 2:**
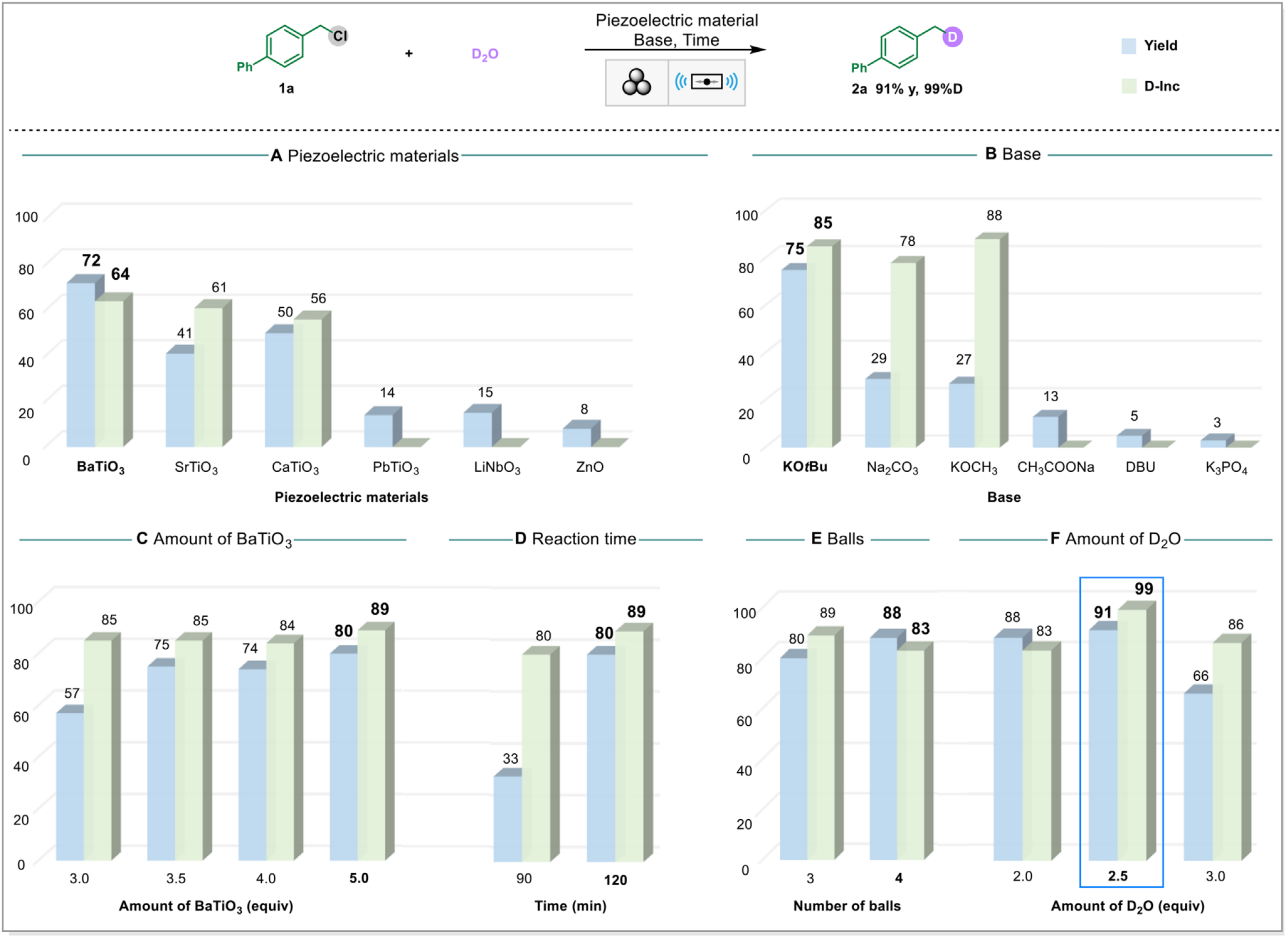
Screening of reaction conditions. Optimized conditions: 4‐(Chloromethyl)biphenyl (**1a**, 0.2 mmol), BaTiO_3_ (5.0 equiv.), KO*t*Bu (1.0 equiv.), D_2_O (2.5 equiv.), ball milling under air for 2 h. Reactions were conducted in a stainless‐steel milling jar (5 mL) at 30 Hz using four stainless‐steel balls (diameter: 7 mm). Yield determined by GC using dodecane as an internal standard. Deuterium incorporation determined by ^1^H NMR spectrum. Deuterium incorporation measurements are not conducted when the GC yield of the product is less than 25%.

Initially, we employed 4‐(chloromethyl) biphenyl (**1a**) and D_2_O (2.0 equiv.) as model substrates, together with a piezoelectric material (3.5 equiv.) and a base (1.0 equiv.), to investigate the optimal conditions for the deuteration reaction under ball milling at a frequency of 30 Hz (**Figure** **2**). Various piezoelectric materials were evaluated, revealing that BaTiO_3_ was the most effective (Figure 2A). Screening of different bases showed that KO*t*Bu outperformed others in terms of efficiency (Figure 2B). The optimal loading of BaTiO_3_ was determined to be 5.0 equivalents (Figure 2C). Attempts to reduce the reaction time from 2 to 1.5 h resulted in a significant decrease in yield (Figure 2D). Additional experiments investigating the number of stainless‐steel balls demonstrated that using four balls achieved more effective grinding and improved reaction efficiency (Figure 2E). Furthermore, adjusting the amount of D_2_O within the range of 2.0–4.0 equivalents revealed that 2.5 equivalents yielded the best result, producing product **2a** with a yield of 91% and 99% deuterium incorporation (D‐inc.) (Figure 2F; Table S6, Supporting Information).

**Figure 3:**
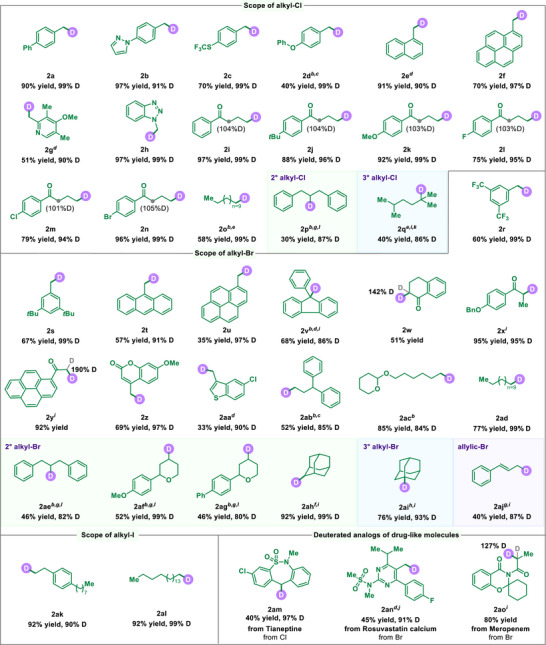
Substrates scope of alkyl halides^a^. *^a^* Conditions: **1a** (0.2 mmol), BaTiO_3_ (5.0 equiv), KO*t*Bu (1.0 equiv), D_2_O (2.5 equiv), ball milling under air for 2 h, Reactions were conducted in stainless‐steel milling jar (5 mL) at 30 Hz using four stainless‐steel balls (diameter: 7 mm), isolated yields. *^b^* 35 Hz. *^c^* D_2_O (3.0 equiv). *^d^* 3 h. *^e^* 4 h. *^f^* 5 h. *^g^* 6 h. *^h^* 8 h. *^i^* Et_3_N. *^j^* D_2_O (5.0 equiv). *^k^* D_2_O (10.0 equiv). *^l^* D_2_O (15.0 equiv).

After establishing the optimized conditions for mechanochemical dehalogenative deuteration, we proceeded to investigate the substrate scope of the reaction, with the results presented in **Figure** **3**. First, we tested various alkyl chlorides under the optimal experimental conditions. The benzyl C(sp^3^)‐Cl compounds containing various substituents, including phenyl groups (**2a**), pyrazole (**2b**), and electron‐withdrawing groups such as SCF_3_ (**2c**) and phenoxy (**2d**) groups, could be successfully converted into the desired products with good yields and high deuterium incorporation under mechanochemical conditions. We observed that substrates containing naphthalene and hetero substituents provided the corresponding products in high yields with exceptional deuterium incorporation rates (**2e‐2h**). The reaction also demonstrated compatibility with unactivated alkyl chlorides (**2i‐2o**). Specifically, primary alkyl chlorides containing electron‐donating groups such as tert‐butyl and methoxy, as well halides (F, Cl, Br), were all well‐suited for this transformation (**2j‐2n**). It is noteworthy that if there are protons present at the α position of the carbonyl, a portion of the *α*‐H can be deuterated (**2i‐2n**). This result may be attributed to the electron‐withdrawing effect of the neighboring carbonyl, making it prone to H/D exchange reactions under basic conditions. In addition, long‐chain alkyl chloride was also tolerated in the deuteration reaction (**2o**). Notably, secondary and tertiary alkyl chlorides both participated effectively in this transformation, delivering the desired products (**2p‐2q**) with good D‐incorporation. Subsequently, we examined a series of alkyl bromides in this transformation. Alkyl bromides containing various functionalities, such as benzyl (**2r‐2v**), *α*‐carbonyl moieties (**2w‐2y**), and heterocycles (**2z‐2aa**), were amenable to our mechanochemical deuteration strategy. When a halogen atom occupies the *α*‐position of a carbonyl group, D‐incorporations are varying within 1 to 2 (**2w, 2y, 2ao**). This phenomenon likely arises from the simultaneous processes of dehalogenative deuteration and H/D exchange at the *α*‐position. Alkyl bromides with different carbon chain lengths produced the corresponding products with moderate to good yields and high deuterium incorporation (**2ab‐2ad**). More importantly, the reaction also proved compatible with secondary and tertiary alkyl bromides, efficiently generating deuterated products (**2ae‐2ai**). Remarkably, allylic bromide (**2aj**), which performed poorly in previous studies,^[^ ^9c^ ^]^ was efficiently deuterated with satisfactory D‐incorporation in this protocol. In addition, we assessed the suitability of alkyl iodides; fortunately, they yielded deuteration products with high efficiency and excellent deuterium incorporation (**2ak‐2al**). Using our mechanochemical deuteration method, we successfully performed late‐stage deuteration on drug molecule analogs, further demonstrating the versatility of this approach and its potential for drug modification and discovery (**2am‐2ao**).

**Figure 4:**
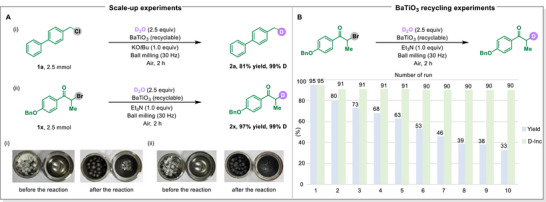
Scale‐up experiments and BaTiO_3_ recycling experiments. A) Scale‐up experiments: Reaction was conducted in a stainless‐steel milling jar (50 mL) at 30 Hz using 50 stainless‐steel balls. B) BaTiO_3_ recycling experiments.

To evaluate the feasibility of this strategy, scale‐up experiments for the production of compound **2a** from alkyl chloride and compound **2x** from alkyl bromide were performed at a 2.5 mmol scale in a 50 mL stainless‐steel milling jar. To our delight, both reactions provided encouraging results. The desired product **2a** was acquired with an 81% yield and 99% D‐Inc., while product **2x** was obtained with a 97% yield and 99% D‐Inc. (**Figure** **4**A). In addition, recycling experiments on the piezoelectric material BaTiO_3_ were conducted (Figure 4B). We found that BaTiO_3_ could be easily separated and recovered from the reaction mixture after each reaction. The recovered BaTiO_3_ was successfully reused in nine additional consecutive dehalogenative deuteration reactions. Throughout these cycles, the deuterium content in the product remained consistent, although the yield gradually declined. This reduction in yield is likely attributable to the diminished piezoelectric properties of BaTiO_3_ due to particle size reduction during the ball milling process. Through sintering and traditional solid‐state processing techniques, it is possible to enlarge the particle size of the piezoelectric materials, facilitating their further reuse.^[^ ^16^ ^]^

**Figure 5:**
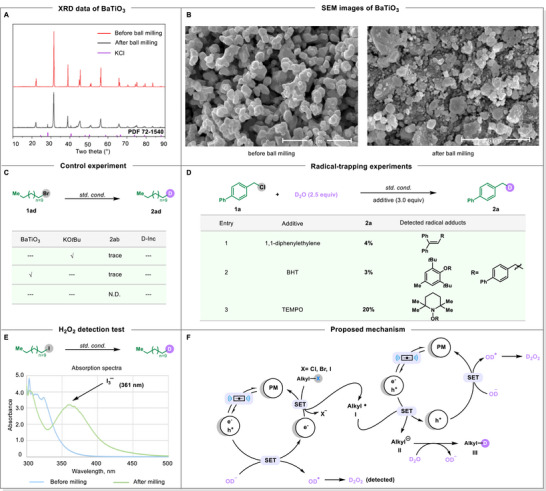
Mechanistic studies. A) XRD data of BaTiO_3_. B) SEM images of BaTiO_3_. C) Control experiments. D) Radical‐trapping experiments. E) H_2_O_2_ detection test: absorption spectra of iodimetry method. F) Proposed mechanism.

To elucidate the reaction mechanism, we undertook a series of studies (**Figure** **5**). Initially, we performed high‐resolution X‐ray diffraction (XRD) and scanning electron microscopy (SEM) tests on BaTiO_3_ nanoparticles (Figure 5A,B). Comparison of the XRD small additional peaks detected post‐milling were identified as KCl (PDF 72‐1540), likely formed during the reaction. SEM analysis revealed that ball milling induced shape deformation in the BaTiO_3_ particles (Figure 5B). Moreover, repeated milling cycles progressively reduced the average particle size of BaTiO_3_ (For more details, see Figures S2–S5, Supporting Information). These test results suggest that the piezoelectric potential induced by mechanical stress in BaTiO_3_ particles plays an important role for the reaction.^[^ ^13^ ^]^

To further investigate the reaction mechanism, we conducted control experiments and radical trapping studies (Figure 5C,D). The control experiments confirmed that both BaTiO_3_ and KO*t*Bu were essential for dehalogenative deuteriation (Figure 5C). Under standard conditions, the addition of radical scavengers—1,1‐diphenylethylene, butylated hydroxytoluene (BHT), and 2,2,6,6‐tetramethyl‐1‐piperinedinyloxy (TEMPO)—resulted in markedly reduced yields of product **2a**, specifically 4%, 3%, and 20%, respectively (Figure 5D). The subsequent detection of corresponding radical adducts via GC‐MS and LCMS analysis supports the involvement of an alkyl radical in the deuteriation process. Additionally, the formation of D_2_O_2_ was observed during the iodometry method, indicating that the OD^‐^ from D_2_O might act as a reductant in the reaction (Figure 5E; Figure S11, Supporting Information).

Based on mechanistic studies and existing literature,^[^ ^17^ ^]^ we propose a plausible mechanism for the mechanochemical dehalogenative deuteration of alkyl halides (Figure 5F). Initially, OD^‐^ion from D_2_O fills the holes in polarized BaTiO_3_ through electron transfer, thereby enhancing the reducing capability of BaTiO_3_ via a single‐electron oxidation process. Subsequently, BaTiO_3_ undergoes a single electron transfer (SET) process with the alkyl halides, resulting in the formation of the corresponding alkyl radical (**I**) and halide anions. This alkyl radical (**I**) is then reduced to an alkyl anion (**II**) through a successive SET process. Finally, the alkyl anion (**II**) reacts with D_2_O to yield the expected product (**III**), while the OD^‐^ ion replenishes an electron to BaTiO_3_ through electron transfer, restoring it to its original state.

## 3 Conclusion

In conclusion, we have developed a universal strategy for the dehalogenative deuteration of both activated and unactivated sp^3^‐hybridized alkyl halides (Cl, Br, I), catalyzed by piezoelectric materials under mechanical force. This approach demonstrates high reactivity and efficient deuterium incorporation. Distinct from typical mechanisms that initiate through single‐electron reduction, our strategy employs a single‐electron oxidation strategy to trigger subsequent reactions. This method facilitates the conversion of a variety of organic halides, including primary, secondary, and tertiary alkyl halides as well as drug molecule analogs, into deuterated products with high yields, using only stoichiometric amounts of D_2_O. Notably, D_2_O also serves as an electron donor, obviating the need for external reducing agents. Additionally, the recyclability of piezoelectric materials, the rapid reaction time, and the absence of solvents underscore the practicality, economy, and environmental friendliness of this deuteration strategy.

## Conflict of Interest

The authors declare no conflict of interest.

## Supporting information



Supporting Information

## Data Availability

The data that support the findings of this study are available in the supplementary material of this article.
