# Supplementary material for: Mechanochemical Dehalogenative Deuteration of Alkyl Halides Through Piezoelectric Catalysis Initiated by a Single‐Electron Oxidation Strategy
Source: Adv Sci (Weinh). 2025 Aug 30;12(44):e15449. doi: 10.1002/advs.202515449 (PMC12667503; doi:10.1002/advs.202515449)
Supplement: Supplementary file 1 — Supporting Information [file ADVS-12-e15449-s001.pdf]

# Supporting Information

## Mechanochemical Dehalogenative Deuteration of Alkyl Halides through Piezoelectric Catalysis Initiated by a Single-Electron Oxidation Strategy

*Ruiling Qu, Ruoxuan Liu, Xiaochun He, Xuemei Zhang,* and Zhong Lian**

## Table of Contents


1. **General information** .......... S2
2. **General procedure for the dehalogenative deuteration of alkyl halides** .......... S2
3. **Optimization of reaction conditions** .......... S2
4. **The procedure for the scale-up reaction** .......... S5
5. **$BaTiO_3$ recycling experiments** .......... S6
6. **Characterization of $BaTiO_3$ particles by SEM** .......... S6
7. **The XRD patterns of $BaTiO_3$** .......... S7
8. **Radical-trapping experiments** .......... S7
9. **Identification of $D_2O_2$ in the reaction solution using the iodimetry method** .......... S10
10. **Characterization data of products** .......... S11
11. **References** .......... S23
12. **NMR spectra of products 2** .......... S24

## 1. General information

Materials were obtained from commercial suppliers and purified by standard procedures unless otherwise noted. Deuterium oxide (Energy chemical, D, 99.9%), *tet*-$BaTiO_3$ (99%, product No. A66124), $CaTiO_3$ (~2 μm particle size, 99%, product No. A67685), $PbTiO_3$ (product No. A60705), $Li_2TiO_3$ (product No. A60327), $BaCO_3$ (product No. E010537), ZnO (<1 μm particle size, 99%, product No. A64210), $SrTiO_3$ (0.5-5 μm particle size, 99%, product No. E061762) and $LiNbO_3$ (product No. A60320) were purchased from 3A Materials®. Substrates 1p, [33a] 1ae, [9d] 1af, [33b] 1ag, [33b] were synthesized according to the published procedures. Other alkyl halides were purchased from Adamas-beta®, Energy chemical or Bidepharm without further purification. All reactions were performed using grinding vessels in Beijing Gladman vibration ball mill GT300. Both jars and balls were made of stainless steel. Solvents for reactions were purchased from commercial suppliers. $^1$H NMR, $^{19}$F NMR, $^{13}$C NMR spectra were recorded in $CDCl_3$ on 400 MHz and 100 MHz spectrometers. Multiplicity was recorded as follows: s = singlet, brs = broad singlet, d = doublet, t = triplet, q = quartet, m = multiplet. Chemical shifts (δ) were reported with respect to the corresponding solvent residual peak at 7.26 ppm for $CDCl_3$ for $^1$H NMR. $^{13}$C NMR spectra (1H-broadband decoupled) were reported in ppm using the central peak of $CDCl_3$ (77.16 ppm).

GC yields were recorded with a Shimadzu GC-2030 equipped with a Rtx-5 column (30 m × 0.25 mm) and dodecane was added as an internal standard. GC-MS analysis was conducted on Agilent 5977B GC/MSD instrument equipped with a HP-5MS UI column (30 m × 0.25 mm).

High-resolution mass data were recorded on a high-resolution mass spectrometer in the EI mode or ESI mode. The molecular ion $[M]^+$ $[M+H]^+$, $[M+Na]^+$ are given in m/z units. Column chromatography was generally performed on silica gel (300-400 mesh) and reactions were monitored by thin layer chromatography (TLC) using UV light to visualize the course of the reactions.

## 2. General procedure for the dehalogenative deuteration of alkyl halides

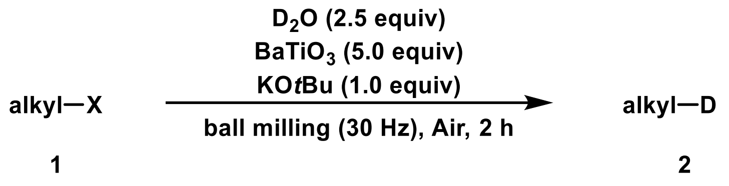


**General Procedure:** A dried 5 mL stainless-steel milling jar was charged with corresponding alkyl halides **1** (0.20 mmol), $BaTiO_3$ (5.0 equiv) under air. Then, KO*t*Bu (1.0 equiv) and $D_2O$ (2.5 equiv) were added. After that, four stainless-steel balls (7 mm, diameter) were added, all performed under ambient conditions. After the jar was closed, it was placed in Gladman vibration ball mill GT300 (30 Hz). After grinding for 2 h (30min*4+5min stop), the reaction mixture was washed with ethyl acetate. The solvent was evaporated and the crude residue was purified through by flash chromatography on silica gel to afford product **2**.

## 3. Optimization of reaction conditions

**Table S1 Optimization of piezoelectric materials (PM)**

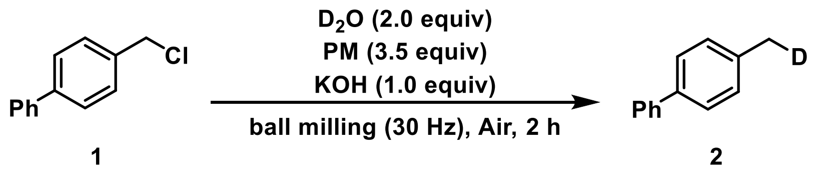

| Entry | Piezoelectric Materials | 2 (GC yield) | D-inc |
|---|---|---|---|
| 1 | $BaTiO_3$ | 72% | 64% |
| 2 | $SrTiO_3$ (0.5-5 μm) | 41% | 61% |
| 3 | $CaTiO_3$ (~2 μm) | 50% | 56% |
| 4 | $PbTiO_3$ | 14% | --- |
| 5 | $LiNbO_3$ | 15% | --- |
| 6 | ZnO (<1 μm) | 8% | --- |
| 7 | $BaCO_3$ | N.D. | --- |
| 8 | $Li_2TiO_3$ | 22% | --- |

Reaction conditions: A dried 5 mL stainless-steel milling jar was charged with corresponding alkyl chloride (**1**, 0.20 mmol), PM (3.5 equiv) under air. Then, KOH (1.0 equiv) and $D_2O$ (2.0 equiv) were added. After that, three stainless-steel balls (7 mm, diameter) were added, all performed under air conditions. After the jar was closed, it was placed in Gladman vibration ball mill GT300 (30 Hz, 2h); the yields were determined by GC analysis using dodecane as an internal standard. Deuterium incorporation was determined by $^1H$ NMR spectrum. Deuterium incorporation measurements are not conducted when the GC yield of the product is less than 25%.

**Table S2 Optimization of base**

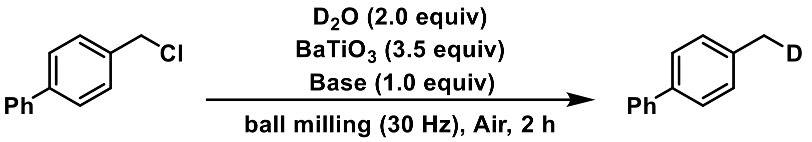


1 2

| Entry | Base | 2 (GC yield) | D-inc |
|---|---|---|---|
| 1 | KO*t*Bu | 75% | 85% |
| 2 | KOH | 72% | 64% |
| 3 | $KOCH_3$ | 27% | 88% |
| 4 | $CH_3COONa$ | 13% | --- |
| 5 | DBU | 5% | --- |
| 6 | $K_3PO_4$ | 3% | --- |
| 7 | $Na_2CO_3$ | 29% | 78% |
| 8 | LiO*t*Bu | 14% | --- |
| 9 | NaO*t*Bu | 29% | 74% |

Reaction conditions: A dried 5 mL stainless-steel milling jar was charged with corresponding alkyl chloride (**1**, 0.20 mmol), $BaTiO_3$ (3.5 equiv.) under air. Then, base (1.0 equiv) and $D_2O$ (2.0 equiv) were added. After that, three stainless-steel balls (7 mm, diameter) were added, all performed under air conditions. After the jar was closed, it was placed in Gladman vibration ball mill GT300 (30 Hz, 2h); the yields were determined by GC analysis using dodecane as an internal standard. Deuterium incorporation was determined by $^1H$ NMR spectrum. Deuterium incorporation measurements are not conducted when the GC yield of the product is less than 25%.

**Table S3 Optimization of the amount of $BaTiO_3$**

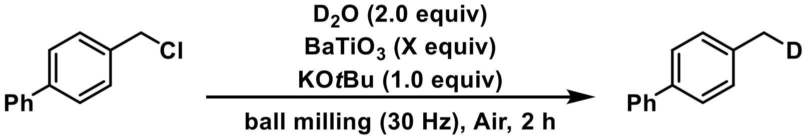


1 2

| Entry | $BaTiO_3$ | 2 (GC yield) | D-inc |
|---|---|---|---|
| 1 | 1.0 eq. | 16% | --- |
| 2 | 2.0 eq | 44% | 83% |
| 3 | 3.0 eq. | 57% | 85% |
| 4 | 3.5 eq. | 75% | 85% |
| 5 | 4.0 eq. | 74% | 84% |
| 6 | 5.0 eq. | 80% | 89% |

Reaction conditions: A dried 5 mL stainless-steel milling jar was charged with corresponding alkyl chloride (**1**, 0.20 mmol), $BaTiO_3$ (X equiv) under air. Then, KO*t*Bu (1.0 equiv) and $D_2O$ (2.0 equiv) were added. After that, three stainless-steel balls (7 mm, diameter) were added, all performed under air conditions. After the jar was close, it was placed in Gladman vibration ball mill GT300 (30 Hz, 2h); the yields were determined by GC analysis using dodecane as an internal standard. Deuterium incorporation was determined by $^1H$ NMR spectrum. Deuterium incorporation measurements are not conducted when the GC yield of the product is less than 25%.

**Table S4 Optimization of reaction time**

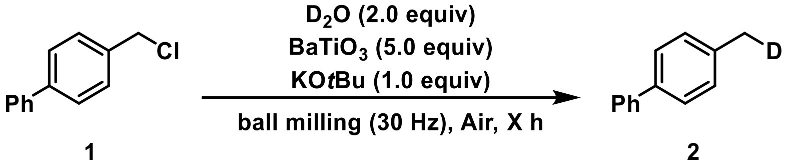


| Entry | Reaction time | 2 (GC yield) | D-inc |
|---|---|---|---|
| 1 | 60 min | 19% | --- |
| 2 | 90 min | 33% | 80% |
| 3 | 120 min | 80% | 89% |

Reaction conditions: A dried 5 mL stainless-steel milling jar was charged with corresponding alkyl chloride (**1**, 0.20 mmol), $BaTiO_3$ (5.0 equiv) under air. Then, KO*t*Bu (1.0 equiv) and $D_2O$ (2.0 equiv) were added. After that, three stainless-steel balls (7 mm, diameter) were added, all performed under air conditions. After the jar was closed, it was placed in Gladman vibration ball mill GT300 (30 Hz, X h); the yields were determined by GC analysis using dodecane as an internal standard. Deuterium incorporation was determined by $^1H$ NMR spectrum. Deuterium incorporation measurements are not conducted when the GC yield of the product is less than 25%.

**Table S5 Optimization of number of balls**

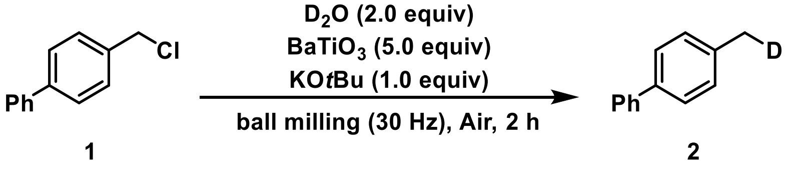


| Entry | Number of balls | 2 (GC yield) | D-inc |
|---|---|---|---|
| 1 | 3 | 80% | 89% |
| 2 | 4 | 88% | 83% |

Reaction conditions: A dried 5 mL stainless-steel milling jar was charged with corresponding alkyl chloride (**1**, 0.20 mmol), $BaTiO_3$ (5.0 equiv) under air. Then, KO*t*Bu (1.0 equiv) and $D_2O$ (2.0 equiv) were added. After that, X stainless-steel balls (7 mm, diameter) were added, all performed under air conditions. After the jar was close, it was placed in Gladman vibration ball mill GT300 (30 Hz, 2h); the yields were determined by GC analysis using dodecane as an internal standard. Deuterium incorporation was determined by $^1$H NMR spectrum. Deuterium incorporation measurements are not conducted when the GC yield of the product is less than 25%.

**Table S6 Optimization of the amount of $D_2O$**

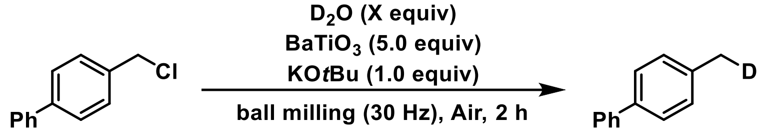


1 2

| Entry | $D_2O$ | 2 (GC yield) | D-inc |
|---|---|---|---|
| 1 | 2.0 eq. | 88% | 83% |
| 2 | 2.5 eq. | 91% | 99% |
| 3 | 3.0 eq. | 66% | 86% |
| 4 | 4.0 eq. | 23% | 88% |

Reaction conditions: A dried 5 mL stainless-steel milling jar was charged with corresponding alkyl chloride (**1**, 0.20 mmol), $BaTiO_3$ (5.0 equiv) under air. Then, KO*t*Bu (1.0 equiv) and $D_2O$ (X equiv) were added. After that, four stainless-steel balls (7 mm, diameter) were added, all performed under air conditions. After the jar was closed, it was placed in Gladman vibration ball mill GT300 (30 Hz, 2h); the yields were determined by GC analysis using dodecane as an internal standard. Deuterium incorporation was determined by $^1$H NMR spectrum.

## 4. The Procedure for the scale-up reaction

**Scheme S1. Scale-up reaction of 1a.**

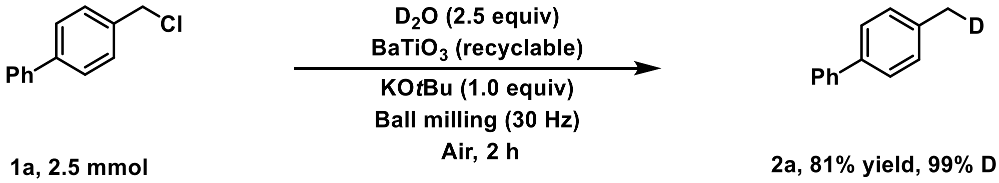


1a, 2.5 mmol 2a, 81% yield, 99% D

A dried 50 mL stainless-steel milling jar was charged with 4-(chloromethyl)-1,1'-biphenyl (**1a**, 2.5 mmol), $BaTiO_3$ (5.0 equiv, 12.5 mmol) under air. Then, KO*t*Bu (1.0 equiv, 2.5 mmol) and $D_2O$ (2.5 equiv, 6.25 mmol) were added. After that, fifty stainless-steel balls (7 mm, diameter) were added, all performed under air conditions. After the jar was closed, it was placed in Gladman vibration ball mill GT300 (30 Hz). After grinding for 2 h, the reaction mixture was washed with ethyl acetate. The solvent was evaporated and the crude residue was purified through by flash chromatography on silica gel (eluted with PE) to afford product **2a** (342 mg, 81% yield, 99% D) as a white solid.

**Scheme S2. Scale-up reaction of 1v.**

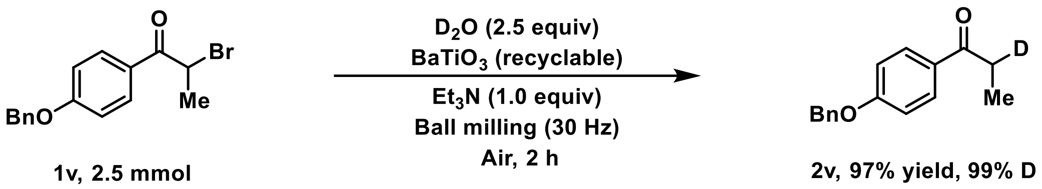


**1v, 2.5 mmol** **2v, 97% yield, 99% D**

A dried 50 mL stainless-steel milling jar was charged with 1-(4-(benzyloxy)phenyl)-2 bromopropan-1-one (**1v**, 2.5 mmol), $BaTiO_3$ (5.0 equiv, 12.5 mmol) under air. Then, $Et_3N$ (1.0 equiv, 2.5 mmol) and $D_2O$ (2.5 equiv, 6.25 mmol) were added. After that, fifty stainless-steel balls (7 mm, diameter) were added under air. After the jar was closed, it was placed in Gladman vibration ball mill GT300 (30 Hz). After grinding for 2 h, the reaction mixture was washed with ethyl acetate. The solvent was evaporated and the crude residue was purified through by flash chromatography on silica gel (eluted with PE/EA=10:1) to afford product **2v** (584 mg, 97% yield, 99% D) as a white solid.

## 5. $BaTiO_3$ recycling experiments

A dried 5 mL stainless-steel milling jar was charged with 1-(4-(benzyloxy)phenyl)-2-bromopropan-1-one (**1v**, 0.2 mmol), $BaTiO_3$ (5.0 equiv, 1.0 mmol) under air. Then, KO*t*Bu (1.0 equiv, 0.2 mmol) and $D_2O$ (2.5 equiv, 0.5 mmol) were added. After that, four stainless-steel balls (7 mm, diameter) were added under air. After the jar was closed, it was placed in Gladman vibration ball mill GT300 (30 Hz). After grinding for 2 h, the reaction mixture was washed with ethyl acetate. $BaTiO_3$ was filtrated and washed with ethyl acetate, and dried over under reduce pressure for 2 h then can be reused for the dehalogenative deuteration of 1-(4-(benzyloxy)phenyl)-2-bromopropan-1-one (**1v**) under the same reaction conditions for at least ten times (Figure S1**)**. The yields of 1-(4-(benzyloxy)phenyl)propan-1-one-2-D (**2v**) was isolated yields. Deuterium incorporation was determined by $^1$H NMR spectrum.

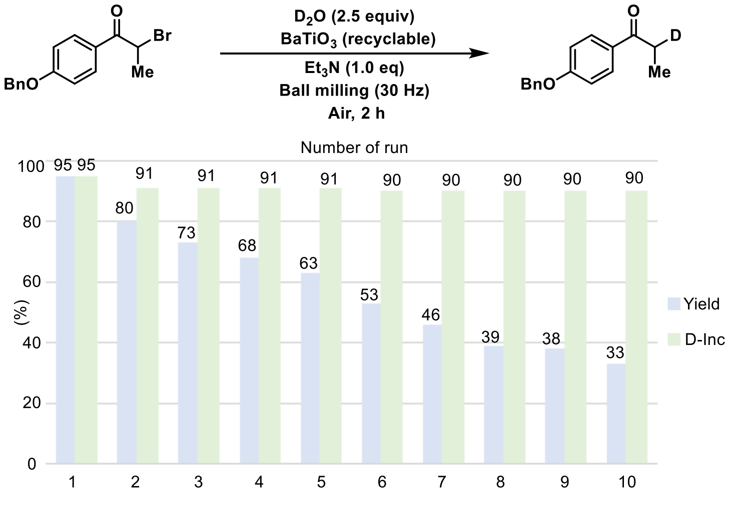


**Figure S1** $BaTiO_3$ recycling experiments were conducted using model reaction

## 6. Characterization of $BaTiO_3$ particles by SEM

In $BaTiO_3$ recovery experiments, samples were taken for SEM characterization after being ground once, five times, and ten times, respectively. The images are shown in Figure S2 – Figure S5.

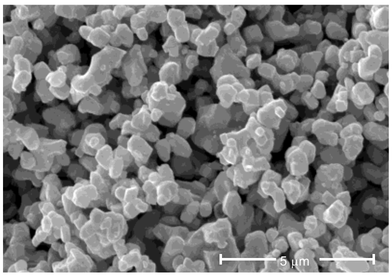


**Figure S2** before ball milling

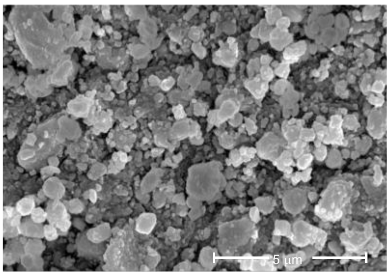


**Figure S3** after one cycle of ball milling

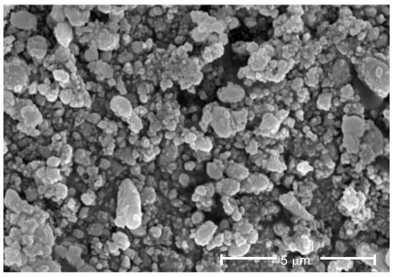


**Figure S4** after five cycles of ball milling

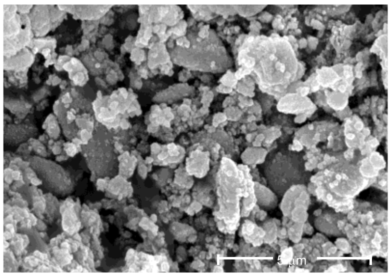


**Figure S5** after ten cycles of ball milling

## 7. The XRD patterns of $BaTiO_3$

The sample of $BaTiO_3$ after ball mill was prepared by general procedure for the dehalogenative deuteration of **1a**. The images are shown in Figure S6.

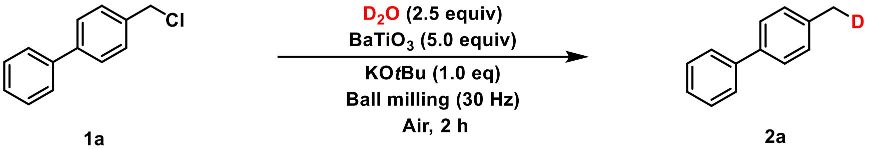


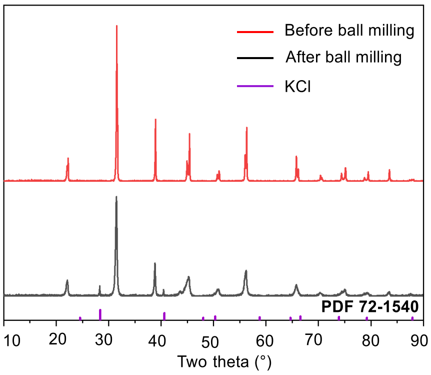


**Figure S6** XRD images of $BaTiO_3$ particles before and after ball milling

## 8. Radical-trapping experiments

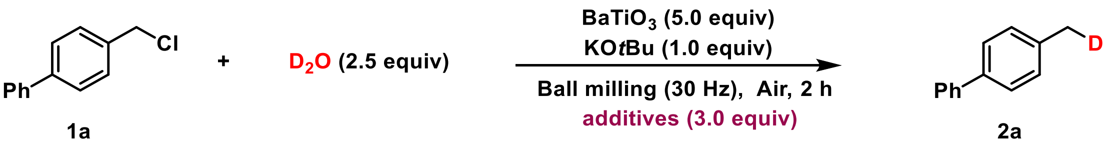


A dried 5 mL stainless-steel milling jar was charged with corresponding alkyl chloride (**1a**, 0.2 mmol), radical-trapping reagents (3.0 equiv), $BaTiO_3$ (5.0 equiv, 1.0 mmol) under air. Then, KO*t*Bu (1.0 equiv, 0.2 mmol) and $D_2O$ (2.5 equiv, 0.5 mmol) were added. After that, four stainless-steel balls (7 mm, diameter) were added under air. After the jar was close, it was placed in Gladman vibration ball mill GT300 (30 Hz). After grinding for 2 h, the reaction mixture was washed with

ethyl acetate. The yield of **2a** was determined by GC analysis using dodecane as an internal standard. The adduct of alkyl radical with 1,1-diphenylethylene, BHT were detected by GC-MS.

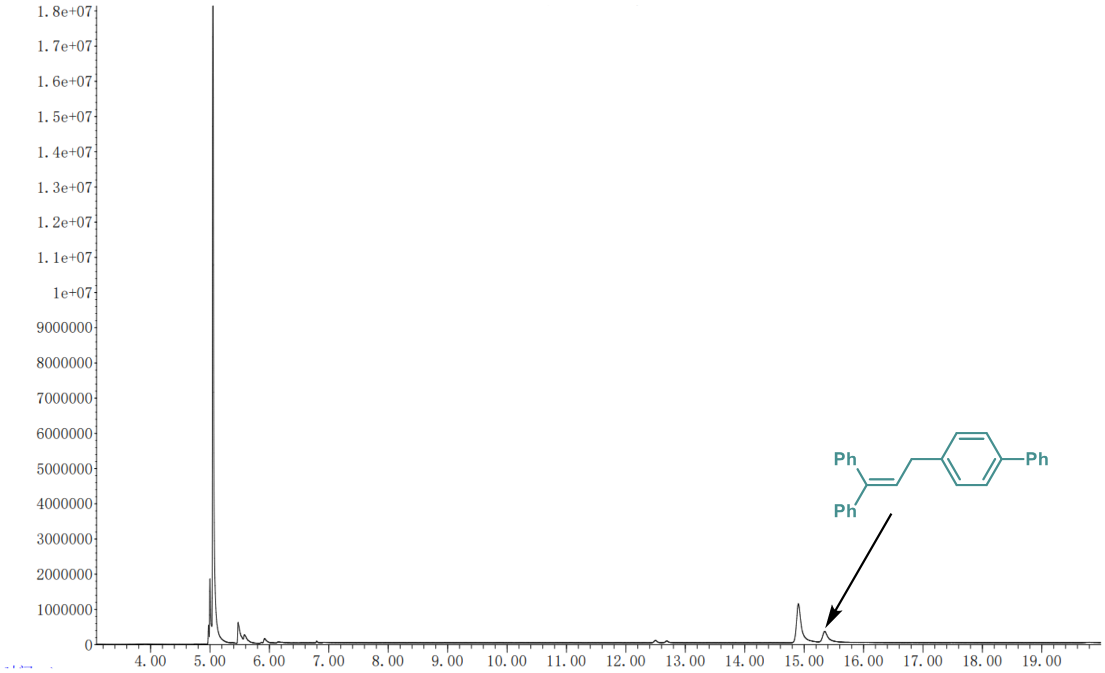


**Figure S7** GC spectra of radical trapping experiment with 1,1-diphenylethylene

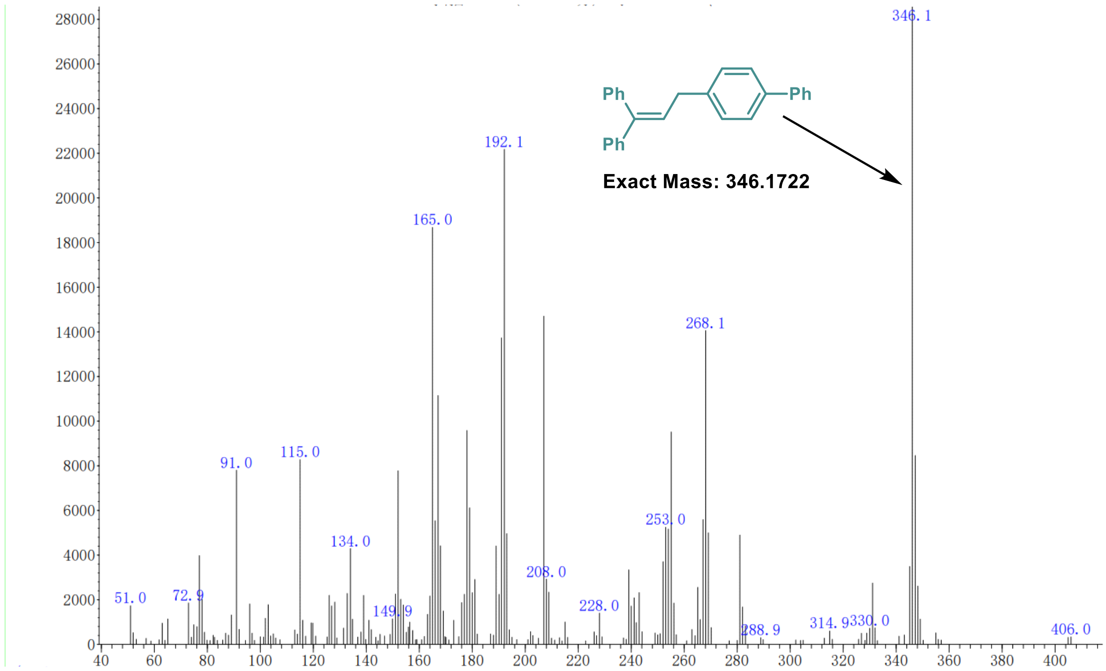


**Figure S8** MS spectra of radical trapping experiment with 1,1-diphenylethylene

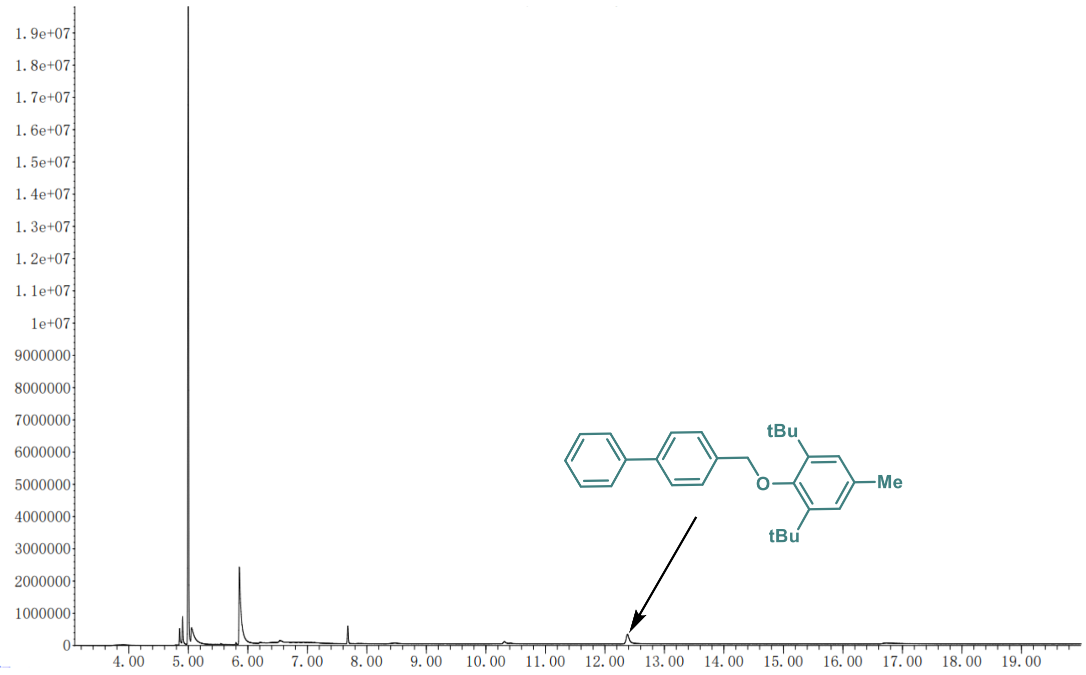


**Figure S9** GC spectra of radical trapping experiment with BHT

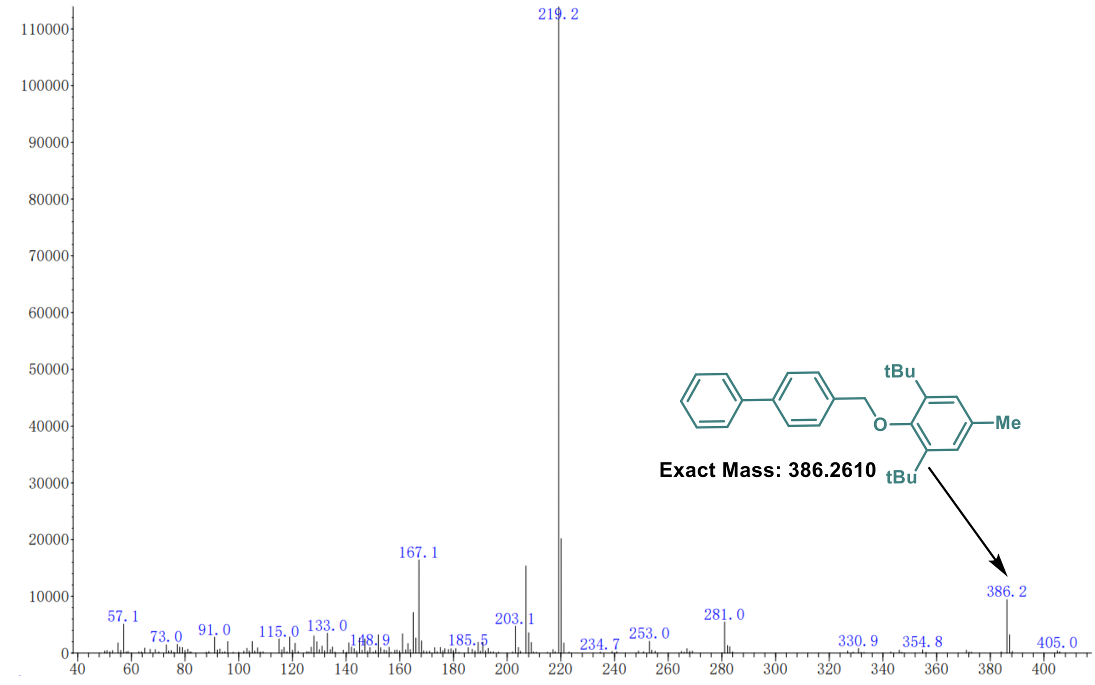


**Figure S10** MS spectra of radical trapping experiment with BHT

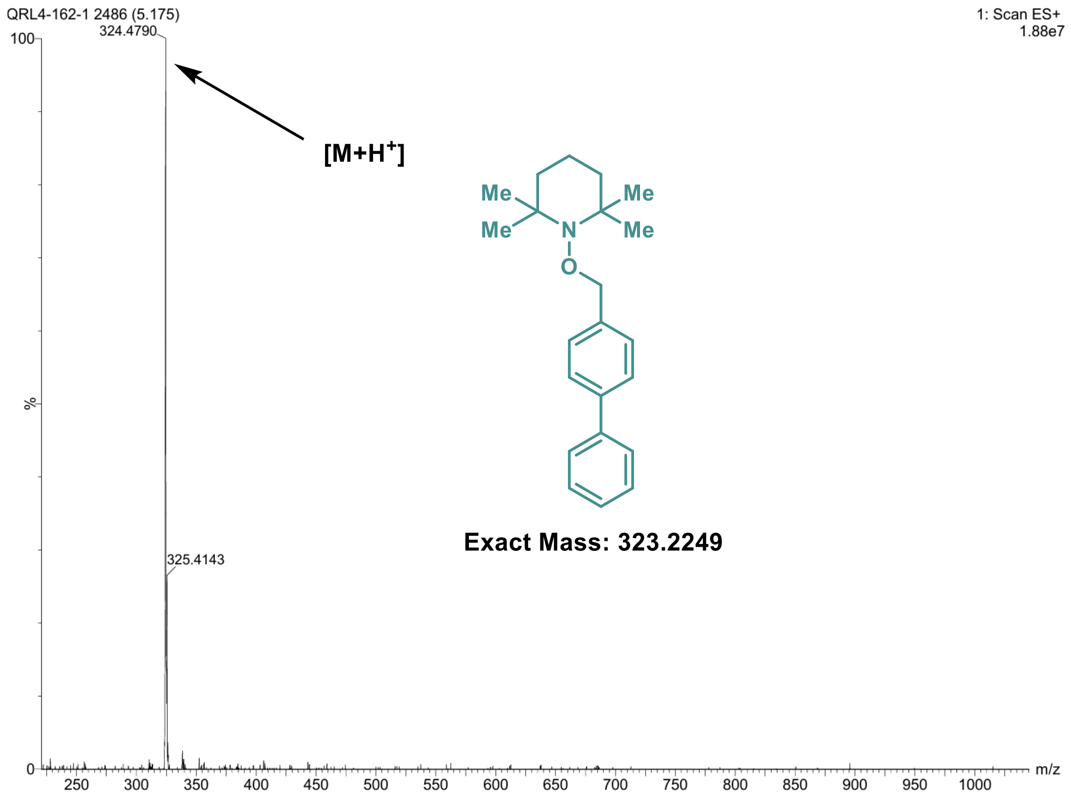


**Figure S11** LCMS spectra of radical trapping experiment with TEMPO

## 9. Identification of $D_2O_2$ in the reaction solution using the iodimetry method.

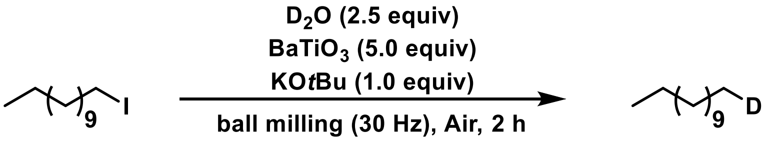


According to previous reports [1-2], a dried 5 mL stainless-steel milling jar was charged with corresponding alkyl iodide (0.2 mmol), $BaTiO_3$ (5.0 equiv, 1.0 mmol) under air. Then, KO*t*Bu (1.0 equiv, 0.2 mmol) and $D_2O$ (2.5 equiv, 0.5 mmol) were added. After that, four stainless-steel balls (7 mm, diameter) were added under air. After the jar was closed, it was placed in Gladman vibration ball mill GT300 (30 Hz). After grinding for 2 h, the reaction mixture was washed with MeCN. Due to the formation of KI in our reaction system, solution can be tested immediately without treatment. The absorption spectrum of $I_3^-$ species formed by the reaction of $I^-$ with $H_2O_2$ was measured. The absorbance at the absorption maximum (λ = 361 nm) of the $I_3^-$ species.

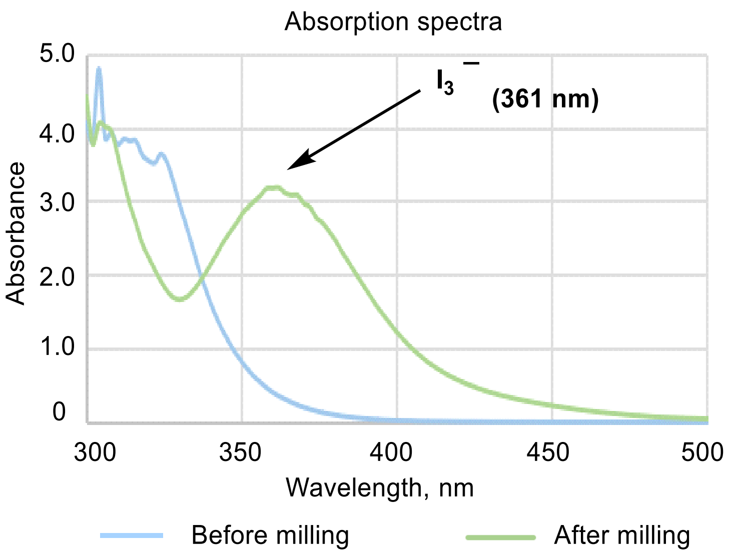


**Figure S12** Absorption spectra of reaction solution before and after milling of the product mixture.

## 10. Characterization data of products

### 4-(Methyl-D)-1,1'-biphenyl (2a)

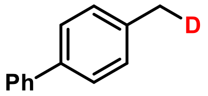


A dried 5 mL stainless-steel milling jar was charged with corresponding alkyl chloride (0.20 mmol), $BaTiO_3$ (5.0 eq.) under air, then add KO*t*Bu (1.0 eq.), $D_2O$ (2.5 eq.) and four stainless-steel balls (7 mm, diameter) were added. After the jar was closed in air, the jar was placed in Gladman vibration ball mill GT300 (30 Hz). After grinding for 2 h, the reaction mixture was washed with ethyl acetate. The solvent was evaporated and the crude residue was purified through by flash chromatography on silica gel (eluted with PE) to afford product 2a (30.4 mg, 90% yield, 99% D) as a white solid. $^1$H NMR (400 MHz, $CDCl_3$) δ 7.49 (d, $J$ = 7.5 Hz, 2H), 7.41 (d, $J$ = 8.1 Hz, 2H), 7.33 (d, $J$ = 15.3 Hz, 2H), 7.26-7.20 (m, 1H), 7.16 (d, $J$ = 7.9 Hz, 2H), 2.29 (dd, $J$ = 5.4, 3.2 Hz, 2.01H). $^{13}$C NMR (100 MHz, $CDCl_3$) δ 141.11, 138.31, 136.99, 136.96, 129.45, 128.68, 126.96, 126.94, 21.18-20.49 (m, C-D). Spectroscopic data match those previously reported in the literature [3].

### 1-(4-(methyl-D)phenyl)-1*H*-pyrazole (2b)

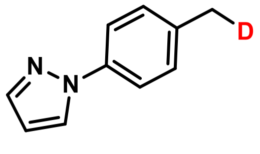


A dried 5 mL stainless-steel milling jar was charged with corresponding alkyl chloride (0.20 mmol), $BaTiO_3$ (5.0 eq.) under air, then add KO*t*Bu (1.0 eq.), $D_2O$ (2.5 eq.) and four stainless-steel balls (7 mm, diameter) were added under air. After the jar was closed in air, the jar was placed in Gladman vibration ball mill GT300 (30 Hz). After grinding for 2 h, the reaction mixture was washed with ethyl acetate. The solvent was evaporated and the crude residue was purified through by flash chromatography on silica gel (eluted with PE/EA=20:1) to afford product 2b (30.9 mg, 97% yield, 91% D) as a white solid. $^1$H NMR (400 MHz, $CDCl_3$) δ 7.87 (d, $J$ = 2.3 Hz, 1H), 7.70 (d, $J$ = 1.7 Hz, 1H), 7.58-7.53 (m, 2H), 7.24 (d, $J$ = 8.4 Hz, 2H), 6.44 (t, $J$ = 2.0 Hz, 1H), 2.38-2.34 (m, 2.09H). $^{13}$C NMR (100 MHz, $CDCl_3$) δ 140.82, 138.03, 136.36, 130.02, 126.84, 119.33, 107.41, 20.73 (t, $J_{C\text{-}D}$ = 20.2 Hz). Spectroscopic data match those previously reported in the literature [18].

**(4-(methyl-D)phenyl)(trifluoromethyl)sulfane (2c)**

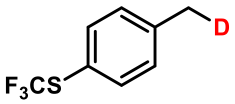


A dried 5 mL stainless-steel milling jar was charged with corresponding alkyl chloride (0.20 mmol), $BaTiO_3$ (5.0 eq.) under air, then add KO*t*Bu (1.0 eq.), $D_2O$ (2.5 eq.) and four stainless-steel balls (7 mm, diameter) were added under air. After the jar was closed in air, the jar was placed in Gladman vibration ball mill GT300 (30 Hz). After grinding for 2 h, the reaction mixture was washed with ethyl acetate. The solvent was evaporated and the crude residue was purified through by flash chromatography on silica gel (eluted with PE) to afford product 2c (27.0 mg, 70% yield, 99% D) as a white solid. $^1H$ NMR (400 MHz, $CDCl_3$) δ 7.56 (d, $J$ = 8.1 Hz, 2H), 7.19 (d, $J$ = 8.2 Hz, 2H), 2.96 (s, 2H). $^{19}F$ NMR (376 MHz, $CDCl_3$) δ -43.22 (s, 3F). Spectroscopic data match those previously reported in the literature [18].

**1-(methyl-D)-4-phenoxybenzene (2d)**

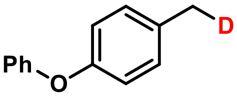


A dried 5 mL stainless-steel milling jar was charged with corresponding alkyl chloride (0.20 mmol), $BaTiO_3$ (5.0 eq.) under air, then add KO*t*Bu (1.0 eq.), $D_2O$ (3.0 eq.) and four stainless-steel balls (7 mm, diameter) were added under air. After the jar was closed in air, the jar was placed in Gladman vibration ball mill GT600 (35 Hz). After grinding for 2 h, the reaction mixture was washed with ethyl acetate. The solvent was evaporated and the crude residue was purified through by flash chromatography on silica gel (eluted with PE/EA=20:1) to afford product 2d (14.8 mg, 40% yield, 99% D) as a colorless oil. $^1H$ NMR (400 MHz, $CDCl_3$) δ 7.38-7.29 (m, 2H), 7.15 (d, $J$ = 8.5 Hz, 2H), 7.10 (t, $J$ = 7.4 Hz, 1H), 7.01 (d, $J$ = 8.0 Hz, 2H), 6.96 (d, $J$ = 8.5 Hz, 2H), 2.92 (s, 2H). $^{13}C$ NMR (100 MHz, $CDCl_3$) δ 157.58, 155.16, 136.62, 129.66, 129.65, 122.90, 119.00, 118.45, 37.27. Spectroscopic data match those previously reported in the literature [3].

**1-(Methyl-D)naphthalene (2e)**

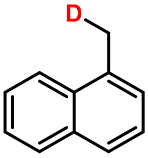


A dried 5 mL stainless-steel milling jar was charged with corresponding alkyl chloride (0.20 mmol), $BaTiO_3$ (5.0 eq.) under air, then add KO*t*Bu (1.0 eq.), $D_2O$ (2.5 eq.) and four stainless-steel balls (7 mm, diameter) were added under air. After the jar was closed in air, the jar was placed in Gladman vibration ball mill GT300 (30 Hz). After grinding for 3 h, the reaction mixture was washed with ethyl acetate. The solvent was evaporated and the crude residue was purified through by flash chromatography on silica gel (eluted with PE) to afford product 2e (26.0 mg, 91% yield, 90% D) as a colorless oil. $^1H$ NMR (400 MHz, $CDCl_3$) δ 8.03-8.00 (m, 1H), 7.89-7.84 (m, 1H), 7.73 (d, $J$ = 8.0 Hz, 1H), 7.57-7.47 (m, 2H), 7.43-7.37 (m, 1H), 7.34 (dd, $J$ = 7.0, 1.2 Hz, 1H), 2.72-2.68 (m, 2.10H). $^{13}C$ NMR (100 MHz, $CDCl_3$) δ 134.21, 133.49, 132.57, 128.49, 126.52, 126.33, 125.68, 125.51, 125.51, 124.08, 19.11 (t, $J_{C\text{-}D}$ = 19.2 Hz). Spectroscopic data match those previously reported in the literature [5].

**1-(Methyl-D)pyrene (2f)**

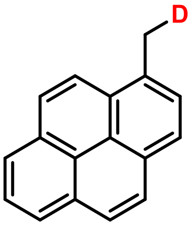


A dried 5 mL stainless-steel milling jar was charged with corresponding alkyl chloride (0.20 mmol), $BaTiO_3$ (5.0 eq.) under air, then add KO*t*Bu (1.0 eq.), $D_2O$ (2.5 eq.) and four stainless-steel balls (7 mm, diameter) were added under air. After the jar was closed in air, the jar was placed in Gladman vibration ball mill GT300 (30 Hz). After grinding for 2 h, the reaction mixture was washed with ethyl acetate. The solvent was evaporated and the crude residue was purified through by flash chromatography on silica gel (eluted with PE/EA=20:1) to afford product 2f (30.4 mg, 70% yield, 97% D) as a white solid. $^1H$

NMR (400 MHz, $CDCl_3$) δ 8.25 (d, $J$ = 9.2 Hz, 1H), 8.18 (dd, $J$ = 7.6, 4.6 Hz, 2H), 8.15-8.07 (m, 2H), 8.06-7.97 (m, 3H), 7.88 (d, $J$ = 7.7 Hz, 1H), 3.00-2.96 (m, 2.03H). $^{13}C$ NMR (100 MHz, $CDCl_3$) the graph is consistent with 2s. Spectroscopic data match those previously reported in the literature [5].

**4-methoxy-3,5-dimethyl-2-(methyl-D)pyridine (2g)**

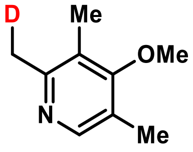


A dried 5 mL stainless-steel milling jar was charged with corresponding alkyl chloride (0.20 mmol), $BaTiO_3$ (5.0 eq.) under air, then add KO*t*Bu (1.0 eq.), $D_2O$ (2.5 eq.) and four stainless-steel balls (7 mm, diameter) were added under air. After the jar was closed in air, the jar was placed in Gladman vibration ball mill GT300 (30 Hz). After grinding for 3 h, the reaction mixture was washed with ethyl acetate. The solvent was evaporated and the crude residue was purified through by flash chromatography on silica gel (eluted with PE/EA=10:1) to afford product 2g (15.5 mg, 51% yield, 90% D) as a colorless oil. $^1H$ NMR (400 MHz, $CDCl_3$) δ 8.07 (s, 1H), 3.70 (s, 3H), 2.40 (dd, $J$ = 5.6, 3.3 Hz, 2.10H), 2.17 (s, 3H), 2.14 (s, 3H). $^{13}C$ NMR (100 MHz, $CDCl_3$) δ 163.49, 156.82, 148.33, 124.18, 123.72, 59.70, 22.40-21.90 (m, C-D), 13.04, 11.47. Spectroscopic data match those previously reported in the literature [18].

**1-(methyl-D)-*1H*-benzo[*d*][1,2,3]triazole (2h)**

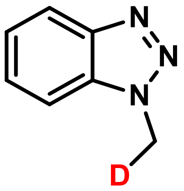


A dried 5 mL stainless-steel milling jar was charged with corresponding alkyl chloride (0.20 mmol), $BaTiO_3$ (5.0 eq.) under air, then add KO*t*Bu (1.0 eq.), $D_2O$ (2.5 eq.) and four stainless-steel balls (7 mm, diameter) were added under air. After the jar was closed in air, the jar was placed in Gladman vibration ball mill GT300 (30 Hz). After grinding for 2 h, the reaction mixture was washed with ethyl acetate. The solvent was evaporated and the crude residue was purified through by flash chromatography on silica gel (eluted with PE/EA=20:1) to afford product 2h (26.0 mg, 97% yield, 99% D) as a yellow solid. $^1H$ NMR (400 MHz, $CDCl_3$) δ 8.05 (d, $J$ = 8.4 Hz, 1H), 7.55-7.46 (m, 2H), 7.40-7.34 (m, 1H), 4.31-4.26 (m, 2.01H). $^{13}C$ NMR (100 MHz, $CDCl_3$) δ 127.28, 123.82, 119.92, 109.08, 33.98 (t, $J_{C\text{-}D}$ = 21.2 Hz).HR-MS (ESI): m/z calcd for $[M+H]^+$ $C_7H_7DN_3^+$, 135.0776; found 135.0772.

**1-phenylbutan-1-one-4-D (2i)**

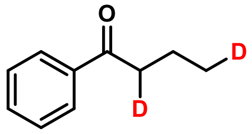


A dried 5 mL stainless-steel milling jar was charged with corresponding alkyl chloride (0.20 mmol), $BaTiO_3$ (5.0 eq.) under air, then add KO*t*Bu (1.0 eq.), $D_2O$ (2.5 eq.) and four stainless-steel balls (7 mm, diameter) were added under air. After the jar was closed in air, the jar was placed in Gladman vibration ball mill GT300 (30 Hz). After grinding for 2 h, the reaction mixture was washed with ethyl acetate. The solvent was evaporated and the crude residue was purified through by flash chromatography on silica gel (eluted with PE/EA=20:1) to afford product 2i (29.0 mg, 97% yield, 99% D) as a colorless oil. $^1H$ NMR (400 MHz, $CDCl_3$) δ 7.99-7.92 (m, 2H), 7.53-7.48 (m, 1H), 7.45-7.38 (m, 2H), 2.66-2.58 (m, 0.96H), 1.22-1.15 (m, 2H), 1.02-0.96 (m, 2.01H).$^{13}C$ NMR (100 MHz, $CDCl_3$) δ 199.59, 136.61, 131.75, 129.52, 127.81, 17.09, 11.87 (d, $J_{C\text{-}D}$=10.1 Hz). Spectroscopic data match those previously reported in the literature [7].

**1-(4-(*tert-butyl*)phenyl)butan-1-one-4-D (2j)**

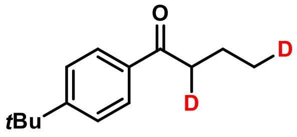


A dried 5 mL stainless-steel milling jar was charged with corresponding alkyl chloride (0.20 mmol), $BaTiO_3$ (5.0 eq.) under air, then add KO*t*Bu (1.0 eq.), $D_2O$ (2.5 eq.) and four stainless-steel balls (7 mm, diameter) were added under air. After the jar was closed in air,

the jar was placed in Gladman vibration ball mill GT300 (30 Hz). After grinding for 2 h, the reaction mixture was washed with ethyl acetate. The solvent was evaporated and the crude residue was purified through by flash chromatography on silica gel (eluted with PE/EA=20:1) to afford product 2j (36.1 mg, 88% yield, 96% D) as a white solid. $^{1}$H NMR (400 MHz, $CDCl_3$) δ 7.97 (d, $J$ = 8.1 Hz, 2H), 7.49 (d, $J$ = 8.1 Hz, 2H), 2.71-2.64 (m, 0.96H), 1.35 (s, 9H), 1.25-1.16 (m, 2H), 1.03-1.00 (m, 2.04H). $^{13}$C NMR (100 MHz, $CDCl_3$) δ 200.16, 156.35, 135.35, 127.92, 125.39, 35.02, 31.07, 16.91, 11.29 (d, $J_{C\text{-}D}$=10.1 Hz). Spectroscopic data match those previously reported in the literature [8].

**1-(4-methoxyphenyl)butan-1-one-4-D (2k)**

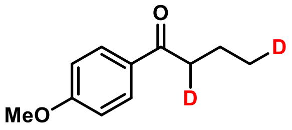


A dried 5 mL stainless-steel milling jar was charged with corresponding alkyl chloride (0.20 mmol), $BaTiO_3$ (5.0 eq.) under air, then add KO*t*Bu (1.0 eq.), $D_2O$ (2.5 eq.) and four stainless-steel balls (7 mm, diameter) were added under air. After the jar was closed in air, the jar was placed in Gladman vibration ball mill GT300 (30 Hz). After grinding for 2 h, the reaction mixture was washed with ethyl acetate. The solvent was evaporated and the crude residue was purified through by flash chromatography on silica gel (eluted with PE/EA=20:1) to afford product 2k (32.9 mg, 92% yield, 99% D) as a colorless oil. $^{1}$H NMR (400 MHz, $CDCl_3$) δ 8.08-7.94 (m, 2H), 7.02-6.89 (m, 2H), 3.87 (s, 3H), 2.66-2.60 (m, 0.97H), 1.23-1.17 (m, 2H), 1.01-0.97 (m, 1.96H). $^{13}$C NMR (100 MHz, $CDCl_3$) δ 199.03, 163.22, 130.96, 130.19, 113.54, 55.41, 16.58, 11.15 (d, $J_{C\text{-}D}$=10.1 Hz). Spectroscopic data match those previously reported in the literature [9].

**1-(4-fluorophenyl)butan-1-one-4-D (2l)**

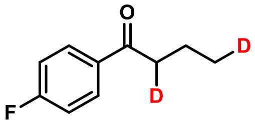


A dried 5 mL stainless-steel milling jar was charged with corresponding alkyl chloride (0.20 mmol), $BaTiO_3$ (5.0 eq.) under air, then add KO*t*Bu (1.0 eq.), $D_2O$ (2.5 eq.) and four stainless-steel balls (7 mm, diameter) were added under air. After the jar was closed in air, the jar was placed in Gladman vibration ball mill GT300 (30 Hz). After grinding for 2 h, the reaction mixture was washed with ethyl acetate. The solvent was evaporated and the crude residue was purified through by flash chromatography on silica gel (eluted with PE/EA=20:1) to afford product 2l (25.0 mg, 75% yield, 95% D) as a colorless oil. $^{1}$H NMR (400 MHz, $CDCl_3$) δ 8.04 (m, 2H), 7.14 (m, 2H), 2.65-2.59 (m, 0.97H), 1.26-1.20 (m, 2H), 1.07-1.00 (m, 2.05H). $^{13}$C NMR (100 MHz, $CDCl_3$) δ 199.03, 165.56 (d, $J_{C\text{-}F}$ =255.5 Hz), 134.32 (d, $J_{C\text{-}F}$ =4.0 Hz), 130.54 (d, $J_{C\text{-}F}$ =10.1 Hz), 115.52 (d, $J_{C\text{-}F}$ =22.2 Hz), 16.99, 11.62 (d, $J_{C\text{-}D}$=10.1 Hz). $^{19}$F NMR (376 MHz, $CDCl_3$) δ -105.98 (s, 1F). Spectroscopic data match those previously reported in the literature [10].

**1-(4-chlorophenyl)butan-1-one-4-D (2m)**

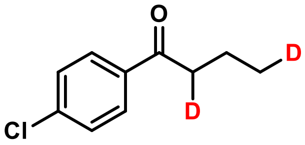


A dried 5 mL stainless-steel milling jar was charged with corresponding alkyl chloride (0.20 mmol), $BaTiO_3$ (5.0 eq.) under air, then add KO*t*Bu (1.0 eq.), $D_2O$ (2.5 eq.) and four stainless-steel balls (7 mm, diameter) were added under air. After the jar was closed in air, the jar was placed in Gladman vibration ball mill GT300 (30 Hz). After grinding for 2 h, the reaction mixture was washed with ethyl acetate. The solvent was evaporated and the crude residue was purified through by flash chromatography on silica gel (eluted with PE/EA=20:1) to afford product 2m (29.0 mg, 79% yield, 94% D) as a colorless oil. $^{1}$H NMR (400 MHz, $CDCl_3$) δ 7.94 (d, $J$ = 8.6 Hz, 2H), 7.43 (d, $J$ = 8.6 Hz, 2H), 2.64-2.57 (m, 0.99H), 1.27-1.19 (m, 2H), 1.07-1.01 (m, 2.06H).$^{13}$C NMR (100 MHz, $CDCl_3$) δ 199.34, 139.06, 136.17, 129.36, 128.72, 17.06, 11.80 (d, $J_{C\text{-}D}$=9.1 Hz). Spectroscopic data match those previously reported in the literature [11].

**1-(4-bromophenyl)butan-1-one-4-D (2n)**

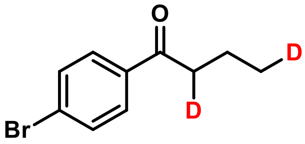


A dried 5 mL stainless-steel milling jar was charged with corresponding alkyl chloride (0.20 mmol), $BaTiO_3$ (5.0 eq.) under air, then add KO*t*Bu (1.0 eq.), $D_2O$ (2.5 eq.) and four stainless-steel balls (7 mm, diameter) were added under air. After the jar was closed in air, the jar was placed in Gladman vibration ball mill GT300 (30 Hz). After grinding for 2 h, the reaction mixture was washed with ethyl acetate. The solvent was evaporated and the crude residue was purified through by flash chromatography on silica gel (eluted with PE/EA=20:1) to afford product 2n (43.6 mg, 96% yield, 99% D) as a colorless oil. $^1$H NMR (400 MHz, $CDCl_3$) δ 7.89-7.83 (m, 2H), 7.63-7.57 (m, 2H), 2.63-2.56 (m, 0.95H), 1.26-1.21 (m, 2H), 1.07-1.03 (m, 2H). $^{13}$C NMR (100 MHz, $CDCl_3$) δ 200.71, 137.96, 132.72, 128.48, 127.99, 17.12, 11.64 (d, $J_{C\text{-}D}$=10.1 Hz). Spectroscopic data match those previously reported in the literature [11].

***n*-Dodecan-1-D (2o)**

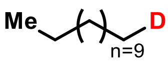


A dried 5 mL stainless-steel milling jar was charged with corresponding alkyl chloride (0.20 mmol), $BaTiO_3$ (5.0 eq.) under air, then add KO*t*Bu (1.0 eq.), $D_2O$ (2.5 eq.) and four stainless-steel balls (7 mm, diameter) were added under air. After the jar was closed in air, the jar was placed in Gladman vibration ball mill GT300 (35 Hz). After grinding for 4 h, the reaction mixture was washed with ethyl acetate. The solvent was evaporated and the crude residue was purified through by flash chromatography on silica gel (eluted with PE) to afford product 2o (19.9 mg, 58% yield, 91% D, determined by GCMS [15]) as a colorless oil. $^1$H NMR (400 MHz, $CDCl_3$) δ 1.26 (s, 20H), 0.88 (t, $J$ = 6.7 Hz, 5H). $^{13}$C NMR (100 MHz, $CDCl_3$) δ 31.93, 31.91, 29.71, 29.67, 29.37, 22.70, 22.61, 14.12-13.63 (m, C-D). Spectroscopic data match those previously reported in the literature [6].

**(propane-1,3-diyl-2-D)dibenzene (2p)**

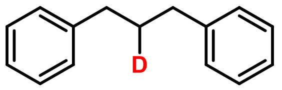


A dried 5 mL stainless-steel milling jar was charged with corresponding alkyl chloride (0.20 mmol), $BaTiO_3$ (5.0 eq.) under air, then add KO*t*Bu (1.0 eq.), $D_2O$ (15.0 eq.) and four stainless-steel balls (7 mm, diameter) were added under air. After the jar was closed in air, the jar was placed in Gladman vibration ball mill GT600 (35 Hz). After grinding for 6 h, the reaction mixture was washed with ethyl acetate. The solvent was evaporated and the crude residue was purified through by flash chromatography on silica gel (eluted with PE) to afford product 2p (12 mg, 30% yield, 87% D, determined by GCMS [32]) as a colorless oil. $^1$H NMR (400 MHz, $CDCl_3$) δ 7.33-7.25 (m, 5H), 7.23-7.16 (m, 5H), 2.67 (t, $J$ = 7.6 Hz, 4H), 2.03-1.92 (m, 1.75H). $^{13}$C NMR (100 MHz, $CDCl_3$) δ142.39, 128.54, 128.40, 125.83, 35.53, 35.44, 33.08. Spectroscopic data match those previously reported in the literature [19].

**2,5-dimethylhexane-2-D (2q)**

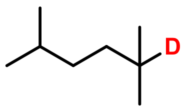


A dried 5 mL stainless-steel milling jar was charged with corresponding alkyl chloride (0.20 mmol), $BaTiO_3$ (5.0 eq.) under air, then add $Et_3N$ (1.0 eq.), $D_2O$ (10.0 eq.) and four stainless-steel balls (7 mm, diameter) were added under air. After the jar was closed in air, the jar was placed in Gladman vibration ball mill GT300 (30 Hz). After grinding for 4 h, the crude mixture was analyzed by $^1$H and $^{13}$C NMR directly. Yield was determined to be 40% (NMR) and deuterium incorporation (86% D) was determined by GCMS [15] .$^1$H NMR (400 MHz, $CDCl_3$) δ 1.53-1.41 (m, 1.9H), 1.18-1.10 (m, 4H), 0.85 (d, $J$ = 6.7 Hz, 12H). $^{13}$C NMR (101 MHz, $CDCl_3$). $^{13}$C NMR (100 MHz, $CDCl_3$) δ 36.82, 28.34, 22.76. Spectroscopic data match those previously reported in the literature [17a].

**1-(methyl-D)-3,5-bis(trifluoromethyl)benzene (2r)**

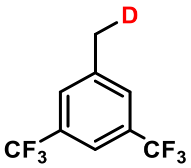


A dried 5 mL stainless-steel milling jar was charged with corresponding alkyl bromide (0.20 mmol), $BaTiO_3$ (5.0 eq.) under air, then add KO*t*Bu (1.0 eq.), $D_2O$ (2.5 eq.) and four stainless-steel balls (7 mm, diameter) were added under air. After the jar was closed in air, the jar was placed in Gladman vibration ball mill GT300 (30 Hz). After grinding for 2 h, the reaction mixture was washed with ethyl acetate. The solvent was evaporated and the crude residue was purified through by flash chromatography on silica gel (eluted with PE) to afford product 2r (27.5 mg, 60% yield, 99% D) as a colorless oil. $^1H$ NMR (400 MHz, $CDCl_3$) δ 7.76 (s, 1H), 7.58 (s, 2H), 3.11 (s, 2H). $^{13}C$ NMR (100 MHz, $CDCl_3$) δ 142.49, 131.92 (dd, $J_{1C\text{-}F}$ = 33.3, $J_{2C\text{-}F}$ = 66.7, 2C), 128.78-128.48 (m, C-F, 2C), 124.58, 121.87, 120.74-120.44 (m, C-F), 36.97. $^{19}F$ NMR (376 MHz, $CDCl_3$) δ -63.01 (s, 6F). HR-MS (EI): m/z calcd for $[M]^+$ $C_9H_5DF_6^+$, 229.0431; found 229.0422.

**1,3-di-*tert*-butyl-5-(methyl-D)benzene (2s)**

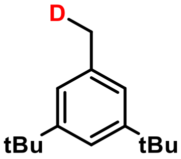


A dried 5 mL stainless-steel milling jar was charged with corresponding alkyl bromide (0.20 mmol), $BaTiO_3$ (5.0 eq.) under air, then add KO*t*Bu (1.0 eq.), $D_2O$ (2.5 eq.) and four stainless-steel balls (7 mm, diameter) were added under air. After the jar was closed in air, the jar was placed in Gladman vibration ball mill GT300 (30 Hz). After grinding for 2 h, the reaction mixture was washed with ethyl acetate. The solvent was evaporated and the crude residue was purified through by flash chromatography on silica gel (eluted with PE) to afford product 2s (27.5 mg, 67% yield, 99% D) as a white solid. $^1H$ NMR (400 MHz, $CDCl_3$) δ 7.27 (t, $J$ = 1.9 Hz, 1H), 7.06 (d, $J$ = 1.9 Hz, 2H), 2.94 (s, 2.01H), 1.33 (s, 18H). $^{13}C$ NMR (100 MHz, $CDCl_3$) δ 150.66, 141.17, 122.72, 119.93, 38.76, 34.89, 31.66. Spectroscopic data match those previously reported in the literature [12].

**9-(Methyl-D)anthracene (2t)**

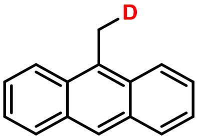


A dried 5 mL stainless-steel milling jar was charged with corresponding alkyl bromide (0.20 mmol), $BaTiO_3$ (5.0 eq.) under air, then add KO*t*Bu (1.0 eq.), $D_2O$ (2.5 eq.) and four stainless-steel balls (7 mm, diameter) were added under air. After the jar was closed in air, the jar was placed in Gladman vibration ball mill GT300 (30 Hz). After grinding for 2 h, the reaction mixture was washed with ethyl acetate. The solvent was evaporated and the crude residue was purified through by flash chromatography on silica gel (eluted with PE) to afford product 2t (22.0 mg, 57% yield, 91% D) as a yellow oil. $^1H$ NMR (400 MHz, $CDCl_3$) δ 8.35 (s, 1H), 8.30 (d, $J$ = 9.6 Hz, 2H), 8.02 (d, $J$ = 7.8 Hz, 2H), 7.56-7.44 (m, 4H), 3.13-3.07 (m, 2.09H). $^{13}C$ NMR (100 MHz, $CDCl_3$) δ 131.57, 130.22, 129.17, 125.40, 125.35, 124.93, 124.80, 13.79 (t, $J_{C\text{-}D}$ = 25.3). Spectroscopic data match those previously reported in the literature [5].

**1-(Methyl-D)pyrene (2u)**

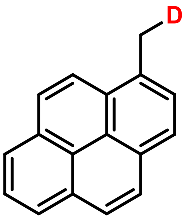


A dried 5 mL stainless-steel milling jar was charged with corresponding alkyl bromide (0.20 mmol), $BaTiO_3$ (5.0 eq.) under air, then add KO*t*Bu (1.0 eq.), $D_2O$ (2.5 eq.) and four stainless-steel balls (7 mm, diameter) were added under air. After the jar was closed in air, the jar was placed in Gladman vibration ball mill GT300 (30 Hz). After grinding for 2 h, the reaction mixture was washed with ethyl acetate. The solvent was evaporated and the crude residue was purified through by flash chromatography on silica gel (eluted with PE/EA=20:1) to afford product 2u (15.2 mg, 35% yield, 97% D) as a white solid. $^1H$ NMR (400 MHz, $CDCl_3$) δ 8.24 (d, $J$ = 9.2 Hz, 1H), 8.20-8.17 (m, 2H), 8.13-8.09 (m, 2H), 8.07-7.98 (m, 3H), 7.88 (d, $J$ = 7.7 Hz,

1H), 3.00-2.97 (m, 2.03H).$^{13}$C NMR (100 MHz, $CDCl_3$) δ 132.12, 131.37, 130.91, 129.65, 129.12, 127.78, 127.47, 127.02, 126.35, 125.70, 124.78, 124.75, 124.73, 124.68, 124.60, 123.62, 19.83-19.20 (m, C-D). Spectroscopic data match those previously reported in the literature [5].

**9-Phenyl-*9H*-fluorene-9-D (2v)**

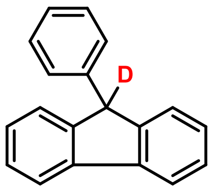


A dried 5 mL stainless-steel milling jar was charged with corresponding alkyl bromide (0.20 mmol), $BaTiO_3$ (5.0 eq.) under air, then add $Et_3N$ (1.0 eq.), $D_2O$ (2.5 eq.) and four stainless-steel balls (7 mm, diameter) were added under air. After the jar was closed in air, the jar was placed in Gladman vibration ball mill GT600 (35 Hz). After grinding for 3 h, the reaction mixture was washed with ethyl acetate. The solvent was evaporated and the crude residue was purified through by flash chromatography on silica gel (eluted with PE) to afford product 2v (33.1 mg, 68% yield, 86% D) as a white solid. $^1$H NMR (400 MHz, $CDCl_3$) δ 7.69 (d, $J$ = 7.6 Hz, 2H), 7.29-7.23 (m, 2H), 7.20 (d, $J$ = 6.6 Hz, 2H), 7.18-7.07 (m, 5H), 7.01-6.94 (m, 2H), 4.93 (s, 0.14H).$^{13}$C NMR (100 MHz, $CDCl_3$) δ 147.82, 141.53, 141.00, 128.65, 128.27, 127.27, 126.78, 125.30, 119.83, 54.47-53.63 (m, C-D). Spectroscopic data match those previously reported in the literature [3].

**3,4-dihydronaphthalen-1(*2H*)-one-2-D (2w)**

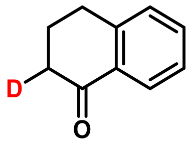


A dried 5 mL stainless-steel milling jar was charged with corresponding alkyl bromide (0.20 mmol), $BaTiO_3$ (5.0 eq.) under air, then add KO*t*Bu (1.0 eq.), $D_2O$ (2.5 eq.) and four stainless-steel balls (7 mm, diameter) were added under air. After the jar was closed in air, the jar was placed in Gladman vibration ball mill GT300 (30 Hz). After grinding for 2 h, the reaction mixture was washed with ethyl acetate. The solvent was evaporated and the crude residue was purified through by flash chromatography on silica gel (eluted with PE/EA=20:1) to afford product 2w (15.0 mg, 51% yield, 142% D) as a yellowish oil. $^1$H NMR (400 MHz, $CDCl_3$) δ 8.03 (dd, $J$ = 7.8, 1.4 Hz, 1H), 7.46 (td, $J$ = 7.5, 1.4 Hz, 1H), 7.30 (t, $J$ = 7.1 Hz, 1H), 7.26 (s, 1H), 2.96 (t, $J$ = 6.1 Hz, 2H), 2.69-2.59 (m, 0.58H), 2.17-2.10 (m, 2H).$^{13}$C NMR (100 MHz, $CDCl_3$) δ 198.21 (t, $J$ = 5.1 Hz), 144.33, 133.21, 132.40, 128.61, 126.90, 126.40, 39.00-37.80 (m, C-D), 29.47 (t, $J$ = 3.0 Hz), 23.01 (t, $J$ = 7.1 Hz). Spectroscopic data match those previously reported in the literature [13].

**1-(4-(benzyloxy)phenyl)propan-1-one-2-D (2x)**

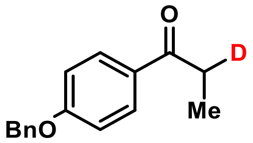


A dried 5 mL stainless-steel milling jar was charged with corresponding alkyl bromide (0.20 mmol), $BaTiO_3$ (5.0 eq.) under air, then add $Et_3N$ (1.0 eq.), $D_2O$ (2.5 eq.) and four stainless-steel balls (7 mm, diameter) were added under air. After the jar was closed in air, the jar was placed in Gladman vibration ball mill GT300 (30 Hz). After grinding for 2 h, the reaction mixture was washed with ethyl acetate. The solvent was evaporated and the crude residue was purified through by flash chromatography on silica gel (eluted with PE/EA=10:1) to afford product 2x (45.8 mg, 95% yield, 95% D) as a white solid. $^1$H NMR (400 MHz, $CDCl_3$) δ 7.97-7.90 (m, 2H), 7.44-7.31 (m, 5H), 7.03-6.97 (m, 2H), 5.11 (s, 2H), 2.97-2.87 (m, 1.05H), 1.20 (d, $J$ = 6.7 Hz, 3H). $^{13}$C NMR (100 MHz, $CDCl_3$) δ 199.65, 162.53, 136.31, 130.34, 130.28, 128.79, 128.33, 127.58, 114.62, 70.19, 31.56-30.97 (m, C-D), 8.49. HR-MS (ESI): m/z calcd for $[M+H]^+$ $C_{16}H_{16}DO_2^+$, 242.1286; found 242.1283.

**1-(pyren-1-yl)ethan-1-one-2-D (2y)**

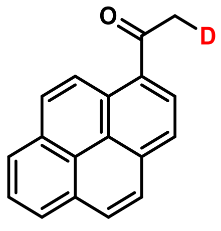

A dried 5 mL stainless-steel milling jar was charged with corresponding alkyl bromide (0.20 mmol), $BaTiO_3$ (5.0 eq.) under air, then add $Et_3N$ (1.0 eq.), $D_2O$ (2.5 eq.) and four stainless-steel balls (7 mm, diameter) were added under air. After the jar was closed in air, the jar was placed in Gladman vibration ball mill GT300 (30 Hz). After grinding for 2 h, the reaction mixture was washed with ethyl acetate. The solvent was evaporated and the crude residue was purified through by flash chromatography on silica gel (eluted with PE/EA=10:1) to afford product 2y (45.1 mg, 92% yield, 190% D) as a yellow solid. $^1$H NMR (400 MHz, $CDCl_3$) δ 9.05 (d, $J$ = 9.4 Hz, 1H), 8.30 (d, $J$ = 8.0 Hz, 1H), 8.22-8.13 (m, 3H), 8.11-7.95 (m, 4H), 2.88-2.83 (m, 1.10H). $^{13}$C NMR (100 MHz, $CDCl_3$) δ 202.15, 133.93, 131.75, 130.97, 130.41, 129.67, 129.56, 129.41, 127.08, 126.99, 126.32, 126.26, 126.02, 124.91, 124.17, 123.89, 30.44-29.56 (m, C-D). HR-MS (EI): m/z calcd for $[M]^+$ $C_{18}H_{11}DO^+$, 245.0945; found 245.0946.

**7-methoxy-4-(methyl-D-*2H*-chromen-2-one (2z)**

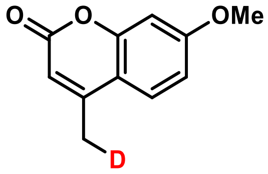

A dried 5 mL stainless-steel milling jar was charged with corresponding alkyl bromide (0.20 mmol), $BaTiO_3$ (5.0 eq.) under air, then add KO*t*Bu (1.0 eq.), $D_2O$ (2.5 eq.) and four stainless-steel balls (7 mm, diameter) were added under air. After the jar was closed in air, the jar was placed in Gladman vibration ball mill GT300 (30 Hz). After grinding for 2 h, the reaction mixture was washed with ethyl acetate. The solvent was evaporated and the crude residue was purified through by flash chromatography on silica gel (eluted with PE) to afford product 2z (26.4 mg, 69% yield, 97% D) as a colorless oil. $^1$H NMR (400 MHz, $CDCl_3$) δ 7.49 (d, $J$ = 8.8 Hz, 1H), 6.87-6.84 (m,1H), 6.81 (d, $J$ = 2.5 Hz, 1H), 6.12 (s, 1H), 3.86 (s, 3H), 2.39-2.37 (m, 2.03H). $^{13}$C NMR (100 MHz, $CDCl_3$) δ 162.58, 161.30, 155.22, 152.58, 125.49, 113.51, 112.25, 111.88, 100.76, 55.71, 18.67-18.17 (m, C-D). Spectroscopic data match those previously reported in the literature [20].

**5-chloro-3-(methyl-D)benzo[*b*]thiophene (2aa)**

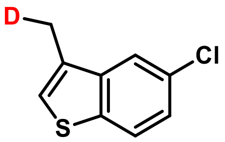

A dried 5 mL stainless-steel milling jar was charged with corresponding alkyl bromide (0.20 mmol), $BaTiO_3$ (5.0 eq.) under air, then add KO*t*Bu (1.0 eq.), $D_2O$ (2.5 eq.) and four stainless-steel balls (7 mm, diameter) were added under air. After the jar was closed in air, the jar was placed in Gladman vibration ball mill GT300 (30 Hz). After grinding for 3 h, the reaction mixture was washed with ethyl acetate. The solvent was evaporated and the crude residue was purified through by flash chromatography on silica gel (eluted with PE) to afford product 2aa (12.1 mg, 33% yield, 90% D) as a colorless oil. $^1$H NMR (400 MHz, $CDCl_3$) δ 7.75 (d, $J$ = 8.5 Hz, 1H), 7.69 (d, $J$ = 2.0 Hz, 1H), 7.31 (dd, $J$ = 8.5, 2.1 Hz, 1H), 7.13 (s, 1H), 2.42-2.39 (m, 2.10H). $^{13}$C NMR (100 MHz, $CDCl_3$) δ 140.91, 138.31, 131.59, 130.23, 124.47, 123.70, 123.45, 121.47, 13.91-13.28 (m, C-D). HR-MS (EI): m/z calcd for $[M]^+$ $C_9H_6DClS^+$, 183.0014; found 183.0015.

**(Propane-1,1-diyl-3-D)dibenzene (2ab)**

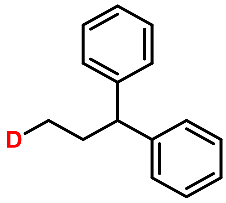

A dried 5 mL stainless-steel milling jar was charged with corresponding alkyl bromide (0.20 mmol), $BaTiO_3$ (5.0 eq.) under air, then add KO*t*Bu (1.0 eq.), $D_2O$ (3.0 eq.) and four stainless-steel balls (7 mm, diameter) were added under air. After the jar was closed in air, the jar was placed in Gladman vibration ball mill GT600 (35 Hz). After grinding for 2 h, the reaction mixture was washed with ethyl acetate. The solvent was evaporated and the crude residue was purified through by flash chromatography on silica gel (eluted with PE)

to afford product 2ab (20.5 mg, 52% yield, 87% D, determined by GCMS [15]) as a colorless oil. $^1$H NMR (400 MHz, $CDCl_3$) δ 7.34-7.27 (m, 8H), 7.24-7.19 (m, 2H), 3.84 (t, $J$ = 7.8 Hz, 1H), 2.16-2.09 (m, 2H), 0.95 (t, $J$ = 7.3 Hz, 2.15H).$^{13}$C NMR (100 MHz, $CDCl_3$) δ 145.26, 128.46, 128.01, 126.11, 53.34, 28.69, 12.90. Spectroscopic data match those previously reported in the literature [5].

**2-((hexyl-6-D)oxy)tetrahydro-*2H*-pyran (2ac)**

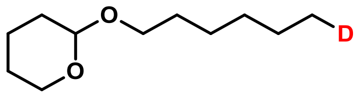


A dried 5 mL stainless-steel milling jar was charged with corresponding alkyl bromide (0.20 mmol), $BaTiO_3$ (5.0 eq.) under air, then add KO*t*Bu (1.0 eq.), $D_2O$ (2.5 eq.) and four stainless-steel balls (7 mm, diameter) were added under air. After the jar was closed in air, the jar was placed in Gladman vibration ball mill GT600 (35 Hz). After grinding for 2 h, the reaction mixture was washed with ethyl acetate. The solvent was evaporated and the crude residue was purified through by flash chromatography on silica gel (eluted with PE) to afford product 2ac (31.8 mg, 85% yield, 84% D, determined by GCMS [15]) as a colorless oil. $^1$H NMR (400 MHz, $CDCl_3$) δ 4.53-4.47 (m, 1H), 3.83-3.78 (m, 1H), 3.70-3.61 (m, 1H), 3.47-3.39 (m, 1H), 3.36-3.27 (m, 1H), 1.83-1.71 (m, 1H), 1.69-1.59 (m, 1H), 1.58-1.38 (m, 6H), 1.33-1.15 (m, 6H), 0.86-0.74 (m, 2.16H). $^{13}$C NMR (100 MHz, $CDCl_3$) δ 98.91, 67.78, 62.41, 31.78, 30.85, 29.79, 26.00, 25.58, 22.70, 19.77, 14.20-1.59 (m, C-D). Spectroscopic data match those previously reported in the literature [21].

***n*-Dodecan-1-D (2ad)**

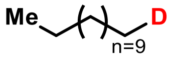


A dried 5 mL stainless-steel milling jar was charged with corresponding alkyl bromide (0.20 mmol), $BaTiO_3$ (5.0 eq.) under air, then add KO*t*Bu (1.0 eq.), $D_2O$ (2.5 eq.) and four stainless-steel balls (7 mm, diameter) were added under air. After the jar was closed in air, the jar was placed in Gladman vibration ball mill GT300 (30 Hz). After grinding for 2 h, the reaction mixture was washed with ethyl acetate. The solvent was evaporated and the crude residue was purified through by flash chromatography on silica gel (eluted with PE) to afford product 2ad (26.4 mg, 77% yield, 99% D, determined by GCMS [15]) as a colorless oil. $^1$H NMR (400 MHz, $CDCl_3$) δ 1.26 (s, 20H), 0.88 (t, $J$ = 6.7 Hz, 5H). $^{13}$C NMR (100 MHz, $CDCl_3$) δ 32.03, 29.80, 29.76, 29.47, 22.79, 22.71, 14.22-13.70 (m, C-D). Spectroscopic data match those previously reported in the literature [6].

**(propane-1,3-diyl-2-D) dibenzene (2ae)**

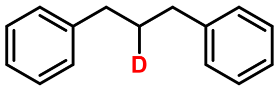


A dried 5 mL stainless-steel milling jar was charged with corresponding alkyl bromide (0.20 mmol), $BaTiO_3$ (5.0 eq.) under air, then add KO*t*Bu (1.0 eq.), $D_2O$ (15.0 eq.) and four stainless-steel balls (7 mm, diameter) were added under air. After the jar was closed in air, the jar was placed in Gladman vibration ball mill GT600 (35 Hz). After grinding for 6 h, the reaction mixture was washed with ethyl acetate. The solvent was evaporated and the crude residue was purified through by flash chromatography on silica gel (eluted with PE) to afford product 2ae (18 mg, 46% yield, 82% D, determined by GCMS [15]) as a colorless oil. $^1$H NMR (400 MHz, $CDCl_3$) δ 7.35-7.28 (m, 4H), 7.26-7.19 (m, 6H), 2.69 (t, $J$ = 7.6 Hz, 4H), 2.05-1.94 (m, 2H). $^{13}$C NMR (100 MHz, $CDCl_3$) δ142.38, 128.53, 128.38, 125.82, 35.53, 35.43, 33.09-33.03 (m, C-D). Spectroscopic data match those previously reported in the literature [19].

**2-(4-methoxyphenyl)tetrahydro-2H-pyran-4-D (2af)**

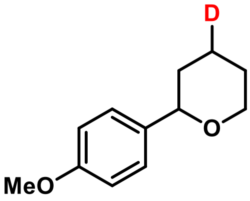


A dried 5 mL stainless-steel milling jar was charged with corresponding alkyl bromide (0.20 mmol), $BaTiO_3$ (5.0 eq.) under air, then add KO*t*Bu (1.0 eq.), $D_2O$ (15.0 eq.) and four stainless-steel balls (7 mm, diameter) were added under air. After the jar was closed in air, the jar was placed in Gladman vibration ball mill GT600 (35 Hz). After grinding for 6 h, the reaction mixture was washed with ethyl acetate. The solvent was evaporated and the crude residue was purified through by flash chromatography on silica gel (eluted with PE/EA=20:1) to afford product 2af (20 mg, 52% yield, 99% D, determined by GCMS [15]). $^1$H NMR (400 MHz, $CDCl_3$) 7.30 (d, $J$ = 8.4 Hz, 2H), 6.89 (d, $J$ = 8.4 Hz, 2H), 4.34-4.25 (m, 1H), 4.20-4.09 (m, 1H), 3.82 (s, 3H), 3.69-3.56 (m, 1H), 1.99-1.92 (m, 1H), 1.86-1.78 (m, 1H), 1.75-1.55 (m, 4H). $^{13}$C NMR (100 MHz, $CDCl_3$) δ 158.9, 135.7, 127.2, 113.7, 79.9, 69.1, 55.4, 33.9, 26.0, 24.1. Spectroscopic data match those previously reported in the literature [17b].

**2-([1,1'-biphenyl]-4-yl)tetrahydro-2H-pyran-4-D (2ag)**

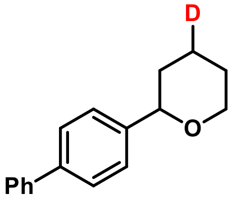


A dried 5 mL stainless-steel milling jar was charged with corresponding alkyl bromide (0.20 mmol), $BaTiO_3$ (5.0 eq.) under air, then add KO*t*Bu (1.0 eq.), $D_2O$ (15.0 eq.) and four stainless-steel balls (7 mm, diameter) were added under air. After the jar was closed in air, the jar was placed in Gladman vibration ball mill GT600 (35 Hz). After grinding for 6 h, the reaction mixture was washed with ethyl acetate. The solvent was evaporated and the crude residue was purified through by flash chromatography on silica gel (eluted with PE/EA=20:1) to afford product 2ag (22 mg, 46% yield, 80% D, determined by GCMS [15]). $^1$H NMR (400 MHz, $CDCl_3$) δ 7.59 (t, J = 7.6 Hz, 4H), 7.47-7.40 (m, 4H), 7.37-7.31 (m, 1H), 4.39 (d, $J$ = 10.6 Hz, 1H), 4.21-4.13 (m, 1H), 3.70-3.60 (m, 1H), 2.01-1.95 (m, 1H), 1.92-1.85 (m, 1H), 1.77-1.56 (m, 4H). $^{13}$C NMR (100 MHz, CDCl3) δ 142.5, 141.1, 140.3, 128.8, 127.23, 127.2, 127.16, 126.4, 80.0, 69.1, 34.1, 26.0, 24.1. Spectroscopic data match those previously reported in the literature [17c].

**adamantane-2-D (2ah)**

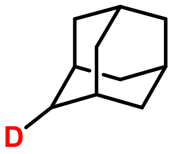


A dried 5 mL stainless-steel milling jar was charged with corresponding alkyl bromide (0.20 mmol), $BaTiO_3$ (5.0 eq.) under air, then add $Et_3N$ (1.0 eq.), $D_2O$ (2.5 eq.) and four stainless-steel balls (7 mm, diameter) were added under air. After the jar was closed in air, the jar was placed in Gladman vibration ball mill GT300 (30 Hz). After grinding for 5 h, the reaction mixture was washed with ethyl acetate. The solvent was evaporated and the crude residue was purified through by flash chromatography on silica gel (eluted with PE) to afford product 2ah (25.2 mg, 92% yield, 97% D, determined by GCMS [15]) as a white solid. $^1$H NMR (400 MHz, $CDCl_3$) δ 1.87 (s, 3.66H), 1.75 (s, 11H).$^{13}$C NMR (100 MHz, $CDCl_3$) δ 37.83, 28.42. Spectroscopic data match those previously reported in the literature [22].

**adamantane-1-D (2ai)**

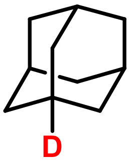


A dried 5 mL stainless-steel milling jar was charged with corresponding alkyl bromide (0.20 mmol), $BaTiO_3$ (5.0 eq.) under air, then add $Et_3N$ (1.0 eq.), $D_2O$ (2.5 eq.) and four stainless-steel balls (7 mm, diameter) were added under air. After the jar was closed in air, the jar was placed in Gladman vibration ball mill GT300 (30 Hz). After grinding for 8 h, the reaction mixture was washed with ethyl acetate. The solvent was evaporated and the crude residue was purified through by flash

chromatography on silica gel (eluted with PE) to afford product 2ai (21.0mg, 76% yield, 91% D, determined by GCMS [15]) as a white solid. $^1$H NMR (400 MHz, $CDCl_3$) δ 1.87 (s, 3.07H), 1.75 (s, 12H).$^{13}$C NMR (100 MHz, $CDCl_3$) δ 37.83,37.82, 28.41. Spectroscopic data match those previously reported in the literature [23].

**(E)-(prop-1-en-1-yl-3-D)benzene (2aj)**

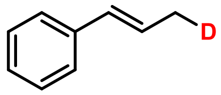


A dried 5 mL stainless-steel milling jar was charged with corresponding alkyl bromide (0.20 mmol), $BaTiO_3$ (5.0 eq.) under air, then add $Et_3N$ (1.0 eq.), $D_2O$ (2.5 eq.) and four stainless-steel balls (7 mm, diameter) were added under air. After the jar was closed in air, the jar was placed in Gladman vibration ball mill GT300 (30 Hz). After grinding for 6 h, the crude mixture was analyzed by $^1$H NMR directly. Yield was determined to be 40% (NMR) and deuterium incorporation (87% D) was determined by GCMS [15]. $^1$H NMR (400 MHz, CDCl3) δ 7.33-7.26 (m, 3H, overlapped with (propyl-1,2,3-$D_3$) benzene), 7.19-7.17 (m, 2H, overlapped with (propyl-1,2,3-$D_3$) benzene), 6.40 (d, $J$ = 16.0 Hz, 1H), 6.22 (m, 1H), 1.89-1.85 (m, 2H). Spectroscopic data match those previously reported in the literature [17d].

**1-(Ethyl-2-D)-4-octylbenzene (2ak)**

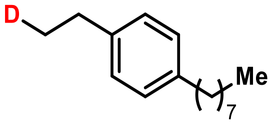


A dried 5 mL stainless-steel milling jar was charged with corresponding alkyl iodide (0.20 mmol), $BaTiO_3$ (5.0 eq.) under air, then add KO*t*Bu (1.0 eq.), $D_2O$ (2.5 eq.) and four stainless-steel balls (7 mm, diameter) were added under air. After the jar was closed in air, the jar was placed in Gladman vibration ball mill GT300 (30 Hz). After grinding for 2 h, the reaction mixture was washed with ethyl acetate. The solvent was evaporated and the crude residue was purified through by flash chromatography on silica gel (eluted with PE) to afford product 2ak (40.4 mg, 92% yield, 99% D, determined by GCMS [15]) as a colorless oil. $^1$H NMR (400 MHz, $CDCl_3$) δ 7.02 (s, 4H), 2.58-2.44 (m, 4H), 1.56-1.44 (m, 2H), 1.27-1.16 (m, 10H), 1.14 (d, $J$ = 7.6 Hz, 2.10H), 0.80 (t, $J$ = 6.7 Hz, 3H). $^{13}$C NMR (100 MHz, $CDCl_3$) δ 141.45, 140.24, 128.39, 127.77, 35.66, 31.99, 31.73, 29.60, 29.50, 29.37, 28.52, 22.77, 15.75, 14.20. Spectroscopic data match those previously reported in the literature [22].

**Octadecane-1-D (2al)**

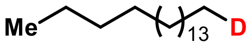


A dried 5 mL stainless-steel milling jar was charged with corresponding alkyl iodide (0.20 mmol), $BaTiO_3$ (5.0 eq.) under air, then add KO*t*Bu (1.0 eq.), $D_2O$ (2.5 eq.) and four stainless-steel balls (7 mm, diameter) were added under air. After the jar was closed in air, the jar was placed in Gladman vibration ball mill GT300 (30 Hz). After grinding for 2 h, the reaction mixture was washed with ethyl acetate. The solvent was evaporated and the crude residue was purified through by flash chromatography on silica gel (eluted with PE) to afford product 2al (47.0 mg, 92% yield, 99% D) as a colorless oil. $^1$H NMR (400 MHz, $CDCl_3$) δ 1.29 (s, 32H), 0.91 (t, $J$ = 6.8 Hz, 5H). $^{13}$C NMR (100 MHz, $CDCl_3$) δ 32.03, 29.80, 29.76, 29.46, 22.78, 14.19. HR-MS (EI): m/z calcd for $[M]^+$ $C_{18}H_{37}D^+$, 255.3031; found 255.3029.

**3-chloro-6-methyl-6,11-dihydrodibenzo[*c,f*][1,2]thiazepine 5,5-dioxide-11-D (2am)**

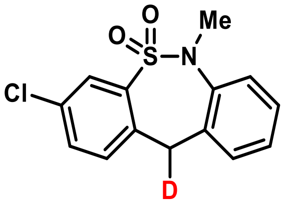


A dried 5 mL stainless-steel milling jar was charged with corresponding alkyl chloride (0.20 mmol), $BaTiO_3$ (5.0 eq.) under air, then add KO*t*Bu (1.0 eq.), $D_2O$ (2.5 eq.) and four stainless-steel balls (7 mm, diameter) were added under air. After the jar was closed in air, the jar was placed in Gladman vibration ball mill GT300 (30 Hz). After grinding for 2 h, the reaction mixture was washed with ethyl acetate. The solvent was evaporated and the crude residue was purified through by flash chromatography on

silica gel (eluted with PE/EA=10:1) to afford product 2am (23.5 mg, 40% yield, 97% D) as a white solid. $^{1}H$ NMR (400 MHz, $CDCl_3$) δ 7.93 (d, J = 2.3 Hz, 1H), 7.46 (dd, J = 8.1, 2.3 Hz, 1H), 7.38-7.31 (m, 3H), 7.30-7.21 (m, 2H), 4.42 (s, 1.03H), 3.25 (s, 3H). $^{13}C$ NMR (100 MHz, $CDCl_3$) δ 140.71, 140.60, 133.41, 132.90, 132.83, 132.68, 132.18, 130.74, 128.54, 127.74, 127.62, 127.55, 40.70-40.05 (m, C-D), 39.55. HR-MS (ESI): m/z calcd for [M+H]+ $C_{14}H_{13}DNO_2S^+$, 261.0803; found 261.0800.

**_N_-(4-(4-fluorophenyl)-6-isopropyl-5-(methyl-D)pyrimidin-2-yl)-_N_-methylmethanesulfonamide (2an)**

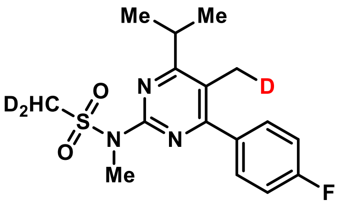


A dried 5 mL stainless-steel milling jar was charged with corresponding alkyl bromide (0.20 mmol), $BaTiO_3$ (5.0 eq.) under air, then add KO*t*Bu (1.0 eq.), $D_2O$ (5.0 eq.) and four stainless-steel balls (7 mm, diameter) were added under air. After the jar was closed in air, the jar was placed in Gladman vibration ball mill GT300 (30 Hz). After grinding for 3 h, the reaction mixture was washed with ethyl acetate. The solvent was evaporated and the crude residue was purified through by flash chromatography on silica gel (eluted with PE/EA=10:1) to afford product 2an (30.4 mg, 45% yield, 91% D) as a white solid. $^{1}H$ NMR (400 MHz, $CDCl_3$) δ 7.60-7.52 (m, 2H), 7.18-7.10 (m, 2H), 3.54 (s, 3H), 3.49 (t, $J$ = 6.6 Hz, 0.83H), 3.33-3.25 (m, 1H), 2.28-2.25 (m, 2.09H), 1.29 (d, $J$ = 6.7 Hz, 6H). $^{13}C$ NMR (100 MHz, $CDCl_3$) δ 175.34, 164.61, 164.34, 161.86, 156.76, 134.71 (d, $J_{C-F}$ = 3.0 Hz), 131.20 (d, $J_{C-F}$ = 9.1 Hz), 118.57 (d, $J_{C-F}$ = 5.1 Hz), 115.18 (d, $J_{C-F}$ = 22.2 Hz), 42.34-41.37 (m, C-D), 33.06, 31.81, 21.20, 14.25-13.65 (m, C-D). $^{19}F$ NMR (376 MHz, $CDCl_3$) δ -111.98 (s, 1F). Spectroscopic data match those previously reported in the literature [24].

**3-(propanoyl-2-D)spiro[benzo[*e*][1,3]oxazine-2,1'-cyclohexan]-4(*3H*)-one (2ao)**

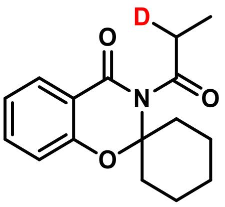


A dried 5 mL stainless-steel milling jar was charged with corresponding alkyl bromide (0.20 mmol), $BaTiO_3$ (5.0 eq.) under air, then add $Et_3N$ (1.0 eq.), $D_2O$ (2.5 eq.) and four stainless-steel balls (7 mm, diameter) were added under air. After the jar was closed in air, the jar was placed in Gladman vibration ball mill GT300 (30 Hz). After grinding for 2 h, the reaction mixture was washed with ethyl acetate. The solvent was evaporated and the crude residue was purified through by flash chromatography on silica gel (eluted with PE/EA=10:1) to afford product 2ao (43.9 mg, 80% yield, 127% D) as a white solid. $^{1}H$ NMR (400 MHz, $CDCl_3$) δ 7.93 (dd, $J$ = 7.8, 1.7 Hz, 1H), 7.53-7.44 (m, 1H), 7.11-7.04 (m, 1H), 6.97 (d, $J$ = 8.2 Hz, 1H), 2.86-2.73 (m, 0.73H), 2.32-2.21 (m, 2H), 2.04 (d, $J$ = 10.9 Hz, 2H), 1.74-1.55 (m, 5H), 1.33-1.22 (m, 1H), 1.18 (d, $J$ = 6.1 Hz, 3H). $^{13}C$ NMR (100 MHz, $CDCl_3$) δ 181.71, 162.73, 15 5.05, 135.54, 128.15, 122.18, 117.39, 117.07, 94.83, 34.95-34.34 (m, C-D), 32.99, 24.22, 22.20, 9.48 (t, $J_{C-D}$ = 6.1 Hz). Spectroscopic data match those previously reported in the literature [14].

## 11. References


[1] K. Ohkubo, T. Kobayashi, S. Fukuzumi, *Angew. Chem. Int. Ed.* **2011**, *50*, 8652-8655.

[2] Z.-R. Wang, C. Zhu, Z.-T. Ni, H. Hojo, H. Einaga, *ACS Catal.* **2022**, *12*, 14976-14989.

[3] W. Ou, X.-D. Xiang, R. Zou, Q. Xu, K. P. Loh, C.-L. Su, *Angew. Chem. Int. Ed.* **2021**, *60*, 6357-6361.

[4] J.-M. Cheng, J. Sheng, X. Cheng, *Org. Lett.* **2023**, *25*, 5602-5607.

[5] B.-Q. He, X.-S. Wu, *Org. Lett.* **2023**, *25*, 6571-6576.

[6] Y.-L. Sun, F.-F. Tan, R.-G. Hu, C.-H. Hu, Y. Li, *Chin. J. Chem.* **2022**, *40*, 1903-1908.

[7] X.-N. Yang, Y.-F. Guo, H.-E. Tong, H.-Y. Guo, *Org. Lett.* **2023**, *25*, 5486-5491.

[8] P. Fan, C. Zhang, L.-C. Zhang, C. Wang, *Org. Lett.* **2020**, *22*, 3875-3878.

[9] D. D. Youmans, H. N. Tran, L. M. Stanley, *Org. Lett.* **2023**, *25*, 3559-3563.

[10] Z.-L. Ma, Y.-H. Wang, *Org. Biomol. Chem.* **2018**, *16*, 7470-7476.

[11] S.-H. Li, C.-Y. Zhang, S. Wang, W.-Q. Yang, X.-R. Fang, S.-L. Fan, Q. Zhang, X.-X. Li, Y.-S. Yang, *Org. Lett.* **2024**, *26*, 1728-1733.

[12] D. Ranolia, I. Avigdori, K. Singh, A. Koronatov, N. Fridman, M. Gandelman, *Org. Lett.* **2022**, *24*, 3915-3919.

[13] G. S. Coumbarides, J. Eames, N. Weerasooriya, *J Labelled Cpd Radiopharm*, **2001**, *44*, 871-879.

[14] K. Kondo, M. Seki, T. Kuroda, T. Yamanaka, T. Iwasaki, *J. Org. Chem.* **1997**, *62*, 2877-2884.

[15] a) C. C. Gruber, G. Oberdorfer, C. V. Voss, J. M. Kremsner, C. O. Kappe, W. Kroutil, *J. Org. Chem.* **2007**, *72*, 5778-5783; b) D. J.Schenk, W. J. S. Lockley, C. S. Elmore, D. Hesk, D. Roberts, *J. Label Compd. Radiopharm* **2016**, *59*, 136-146.

[16] a) A.Villalpando, C. E. Ayala, C. B. Watson, R. Kartika, *J. Org. Chem.* **2013**, *78*, 3989-3996; b) H. B. Zhao, A. J. McMillan, T. Constantin, R. C. Mykura, F. Juliá, D. Leonori, *J. Am. Chem. Soc.* **2021**, *143*, 14806-14813.

[17] a) G. A. Olah, J. L. Grant, R. J. Spear, J. M. Bollinger, A. Serianz, G. Sipos *J. Am. Chem. Soc.* **1976**, *98*, 2501-2507; b) H. -R. Zhao, A. T. Jose, A. Asany, S. M. Khan, M. R. Biscoe. *Org. Lett.* **2022**, *24*, 8714-871; c) T. A. Thane, E. R. Jarvo, *Org. Lett.* **2022**, *24*, 5003-5008; d) Y. K. Takada, J. Caner, S. Kaliyamoorthy, H. Naka, S. Saito, *Chem. Eur. J.* **2017**, *23*, 18025-18032.

[18] D. Wood, S. Lin, *Angew. Chem. Int. Ed.* **2023**, *62*, e202218858.

[19] Y.-J. Li, Z.-Q. Ye, Y.-M. Lin, Y. Liu, Y.-M. Zhang, L. Gong, *Nat. Commun.* **2021**, *12*, 2894-2906.

[20] B.-Z. Chen, D.-W. Ji, B.-C. Zhou, X.-Y. Wang, H. Liu, B.-S. Wan, X.-P. Hu, Q.-A. Chen, *Chinese J. Catal.* **2024**, *59*, 250-259.

[21] P.-F. Li, C.-C. Guo, S.-Y. Wang, D.-K. Ma, T. Feng, Y.-W. Wang, Y.-A. Qiu, *Nat. Commun.* **2022**, *13*, 3774-3779.

[22] A.-Y. Xia, X. Xie, X.-P. Hu, W. Xu, Y.-H. Liu, *J. Org. Chem.* **2019**, *84*, 13841-13857.

[23] J.-W. Hu, J. Cao, L.-W. Xu, *Org. Chem. Front.*, **2024**, *11*, 4757-4761.

[24] D. Wood, S. Lin, *Angew. Chem. Int. Ed.* **2023**, *62*, e202218858.

## 12. NMR spectra of products 2

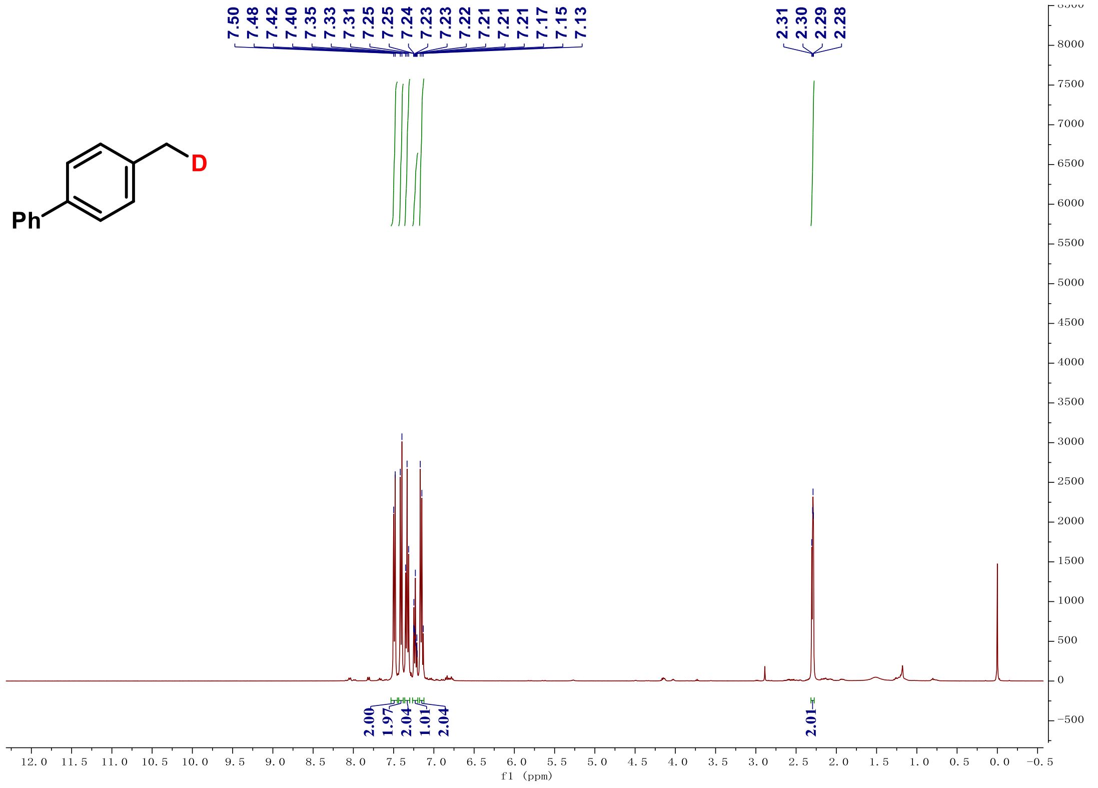


$^{1}$**H NMR** (400 MHz, $CDCl_3$) Spectrum of Compound **2a**

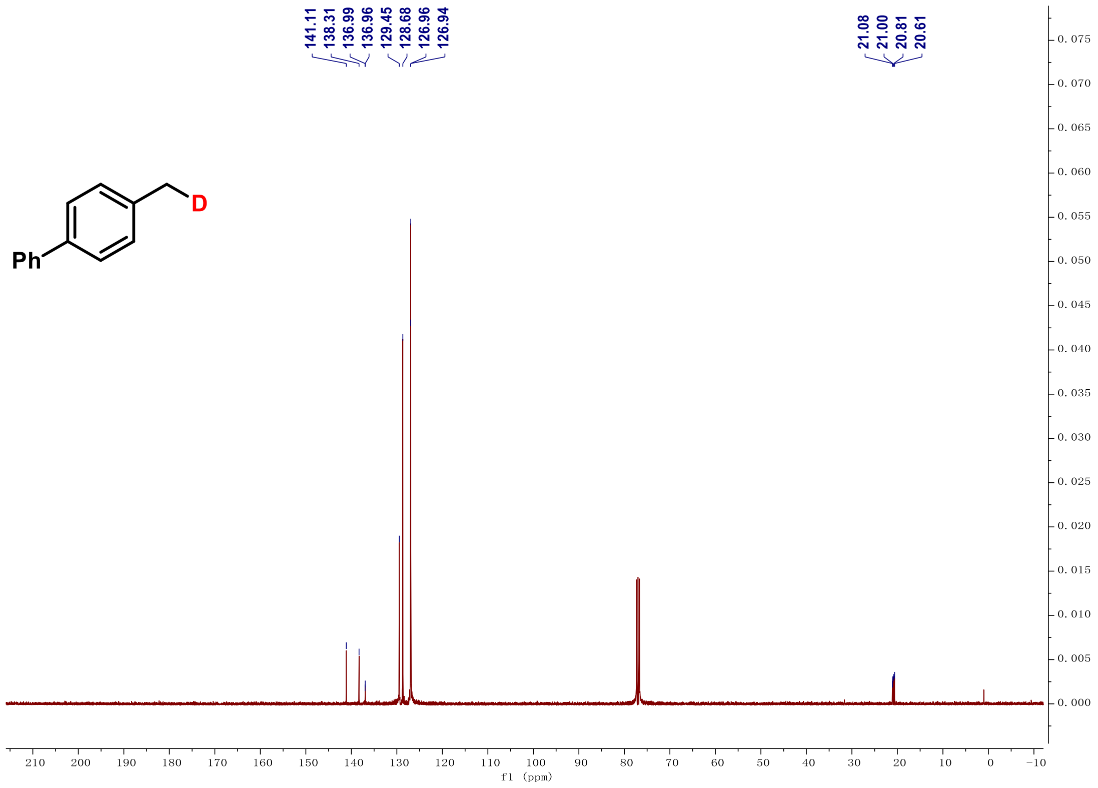


$^{13}$**C NMR** (100 MHz, $CDCl_3$) Spectrum of Compound **2a**

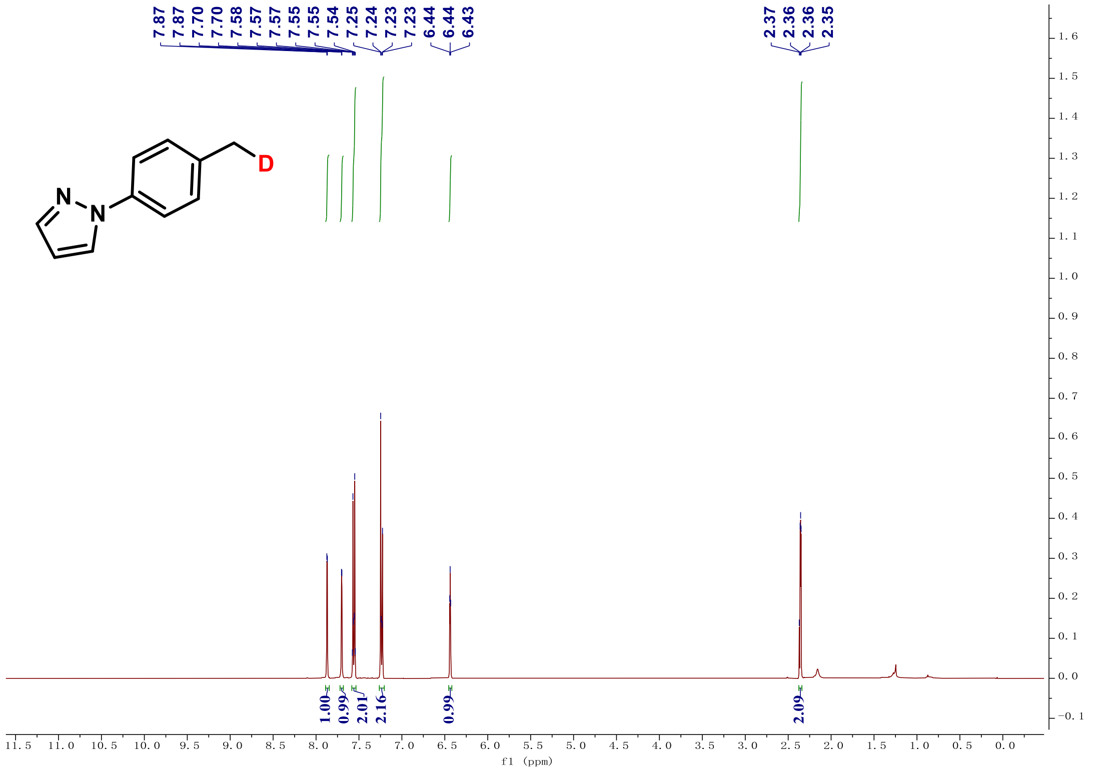


**$^{1}$H NMR** (400 MHz, $CDCl_3$) Spectrum of Compound **2b**

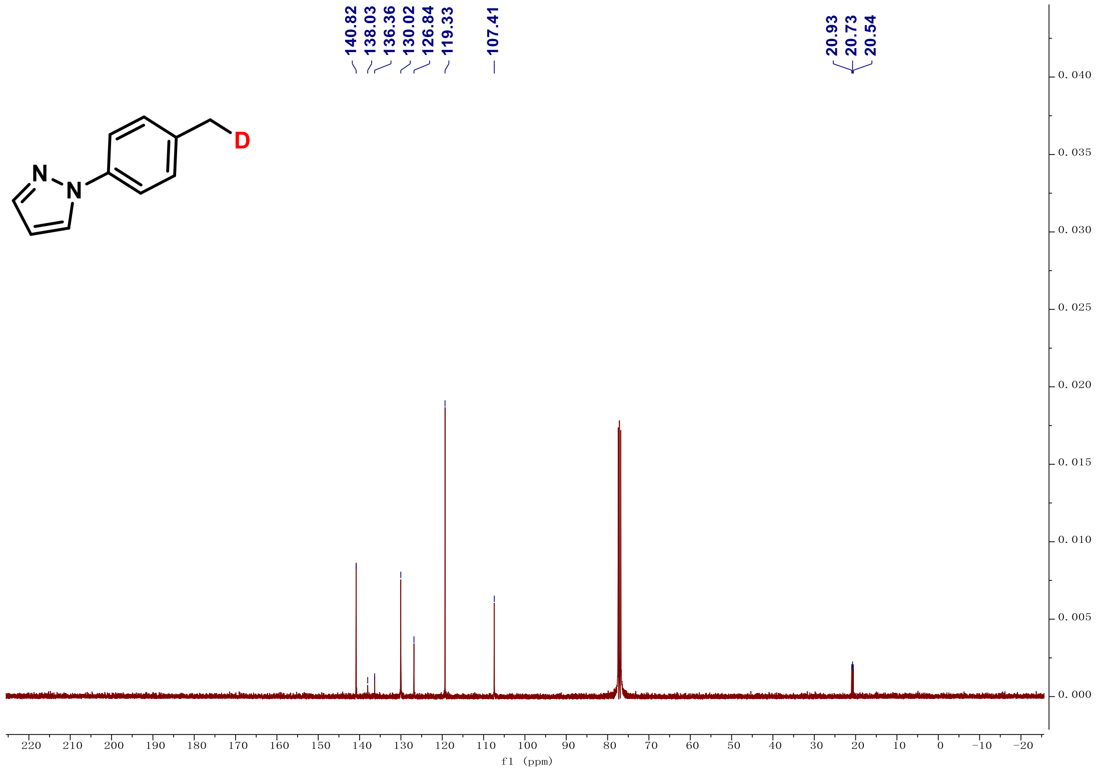


**$^{13}$C NMR** (100 MHz, $CDCl_3$) Spectrum of Compound **2b**

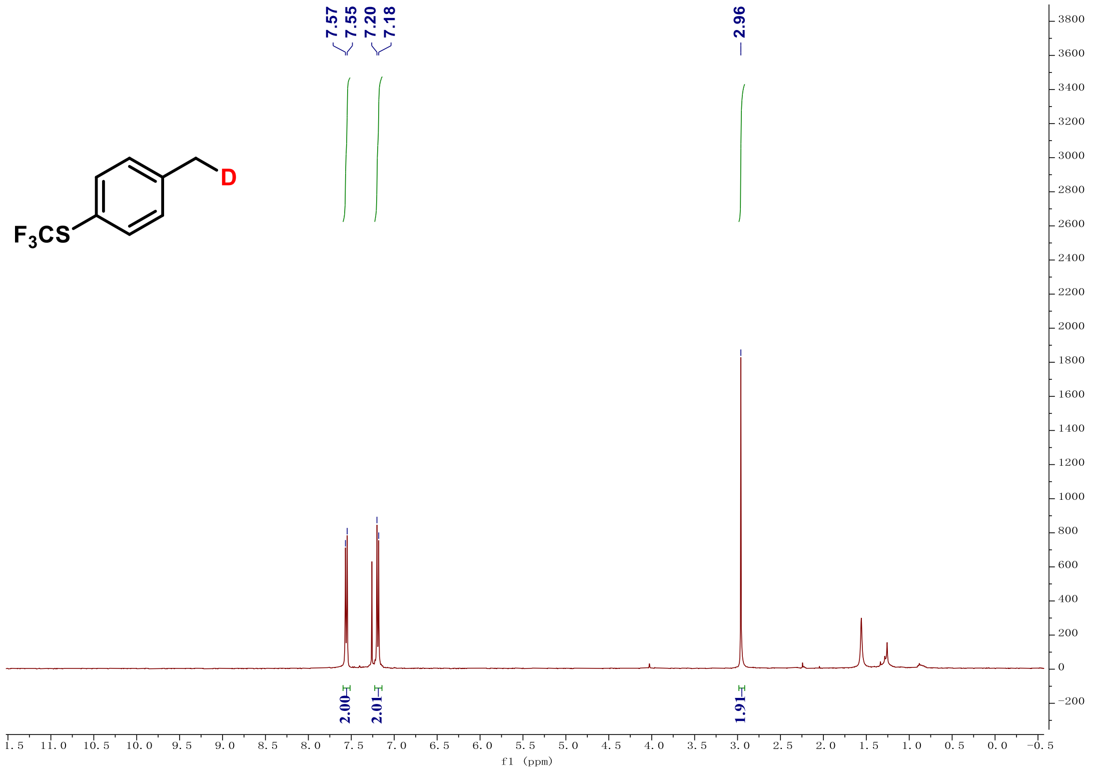


**$^{1}$H NMR** (400 MHz, $CDCl_3$) Spectrum of Compound **2c**

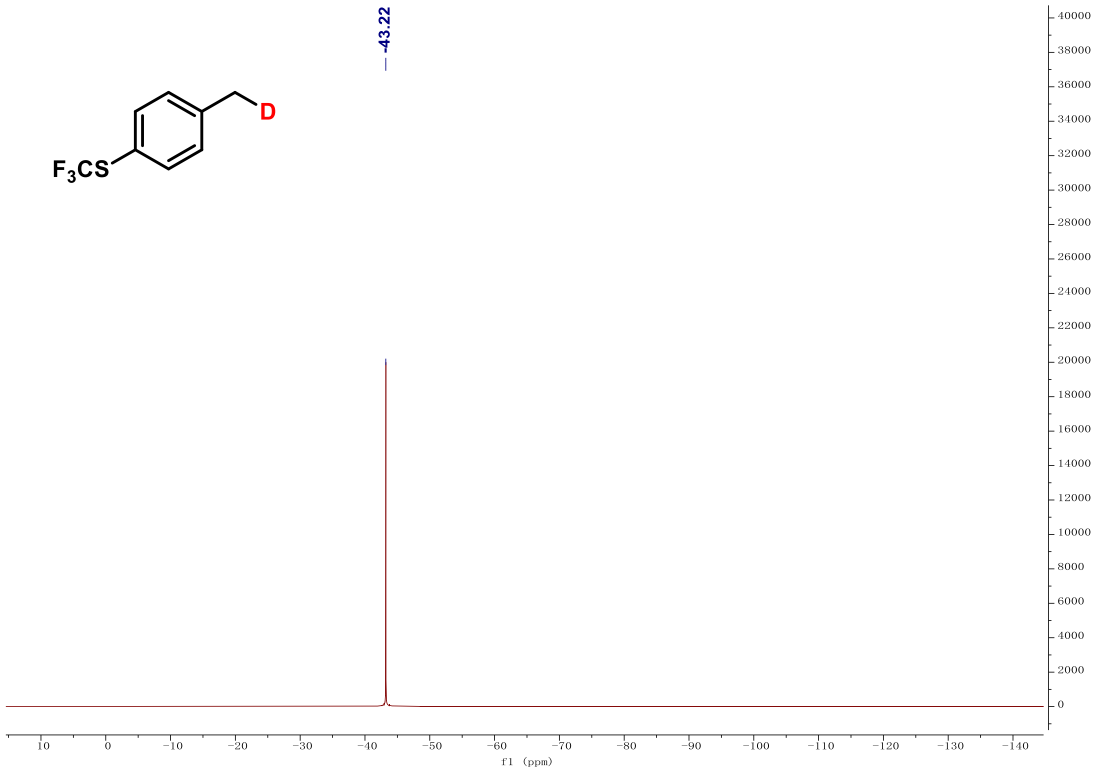


**$^{19}$F NMR** (376 MHz, $CDCl_3$) Spectrum of Compound **2c**

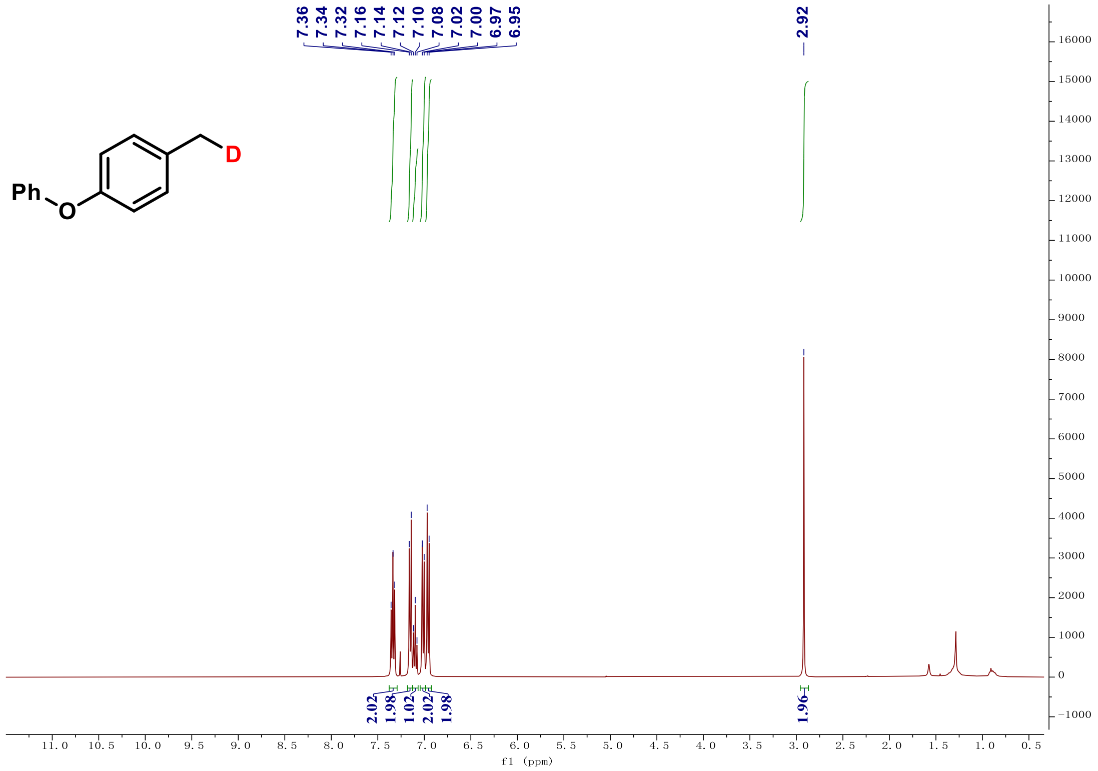


$^{1}$**H NMR** (400 MHz, $CDCl_3$) Spectrum of Compound **2d**

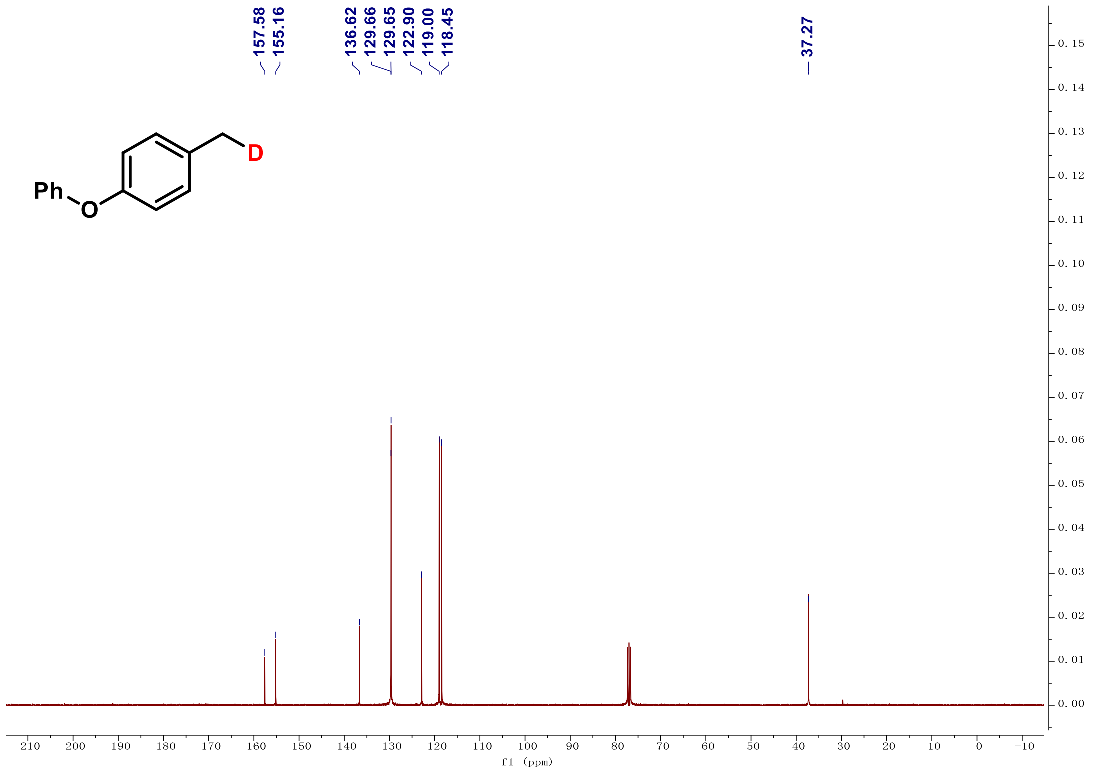


$^{13}$**C NMR** (100 MHz, $CDCl_3$) Spectrum of Compound **2d**

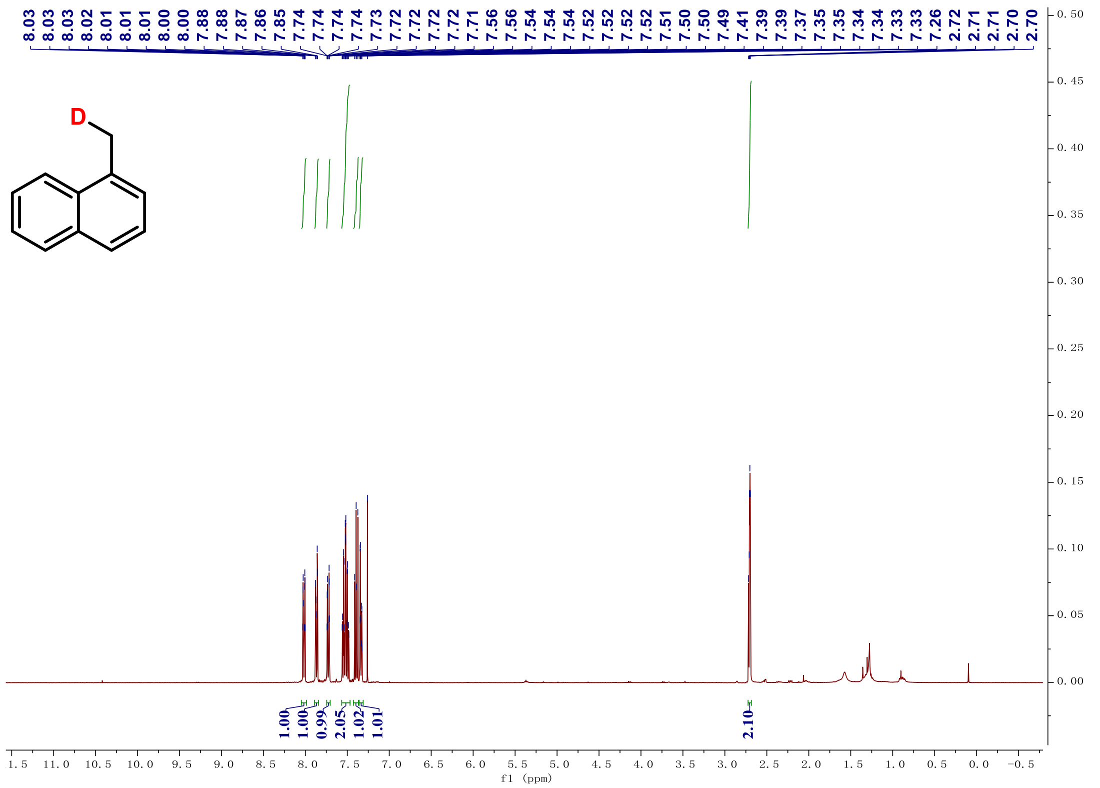


**$^{1}$H NMR** (400 MHz, $CDCl_3$) Spectrum of Compound **2e**

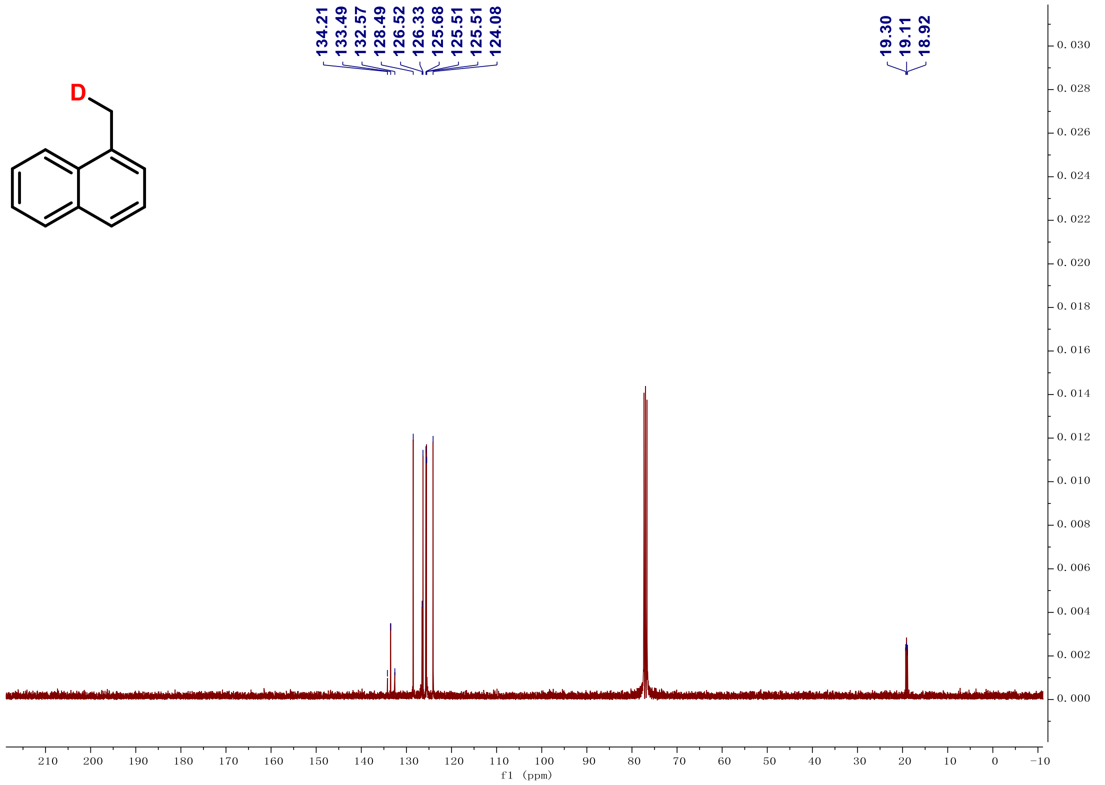


**$^{13}$C NMR** (100 MHz, $CDCl_3$) Spectrum of Compound **2e**

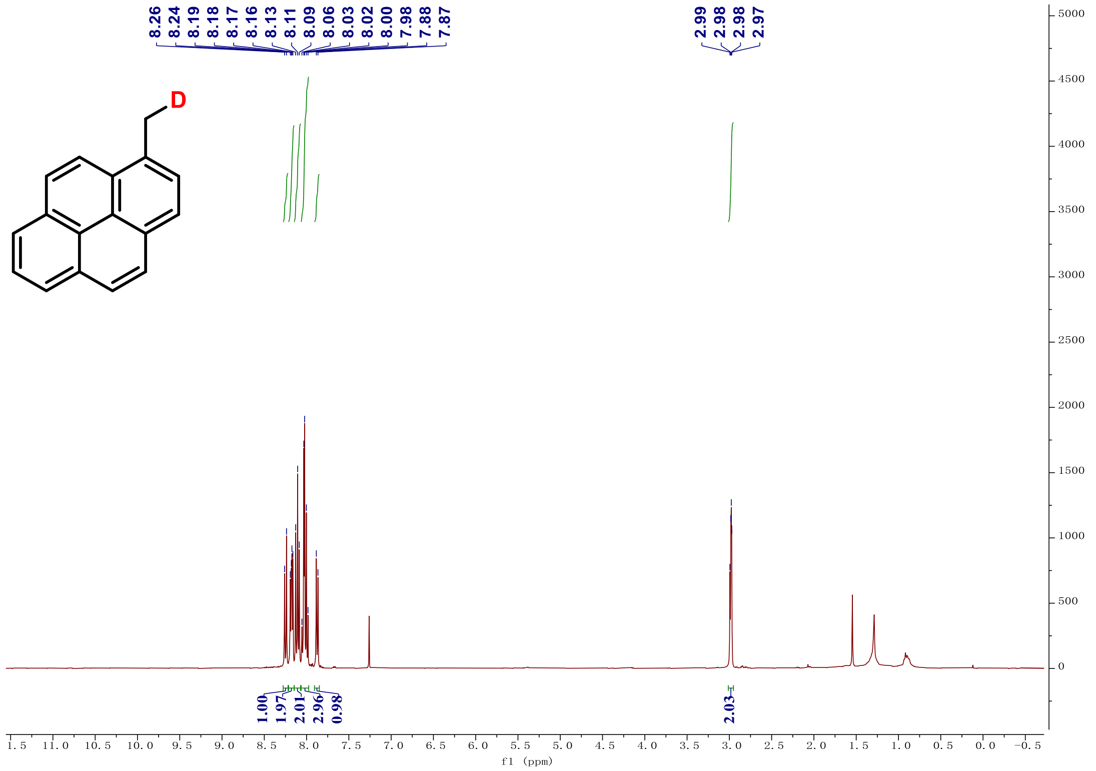


$^1$**H NMR** (400 MHz, $CDCl_3$) Spectrum of Compound **2f**

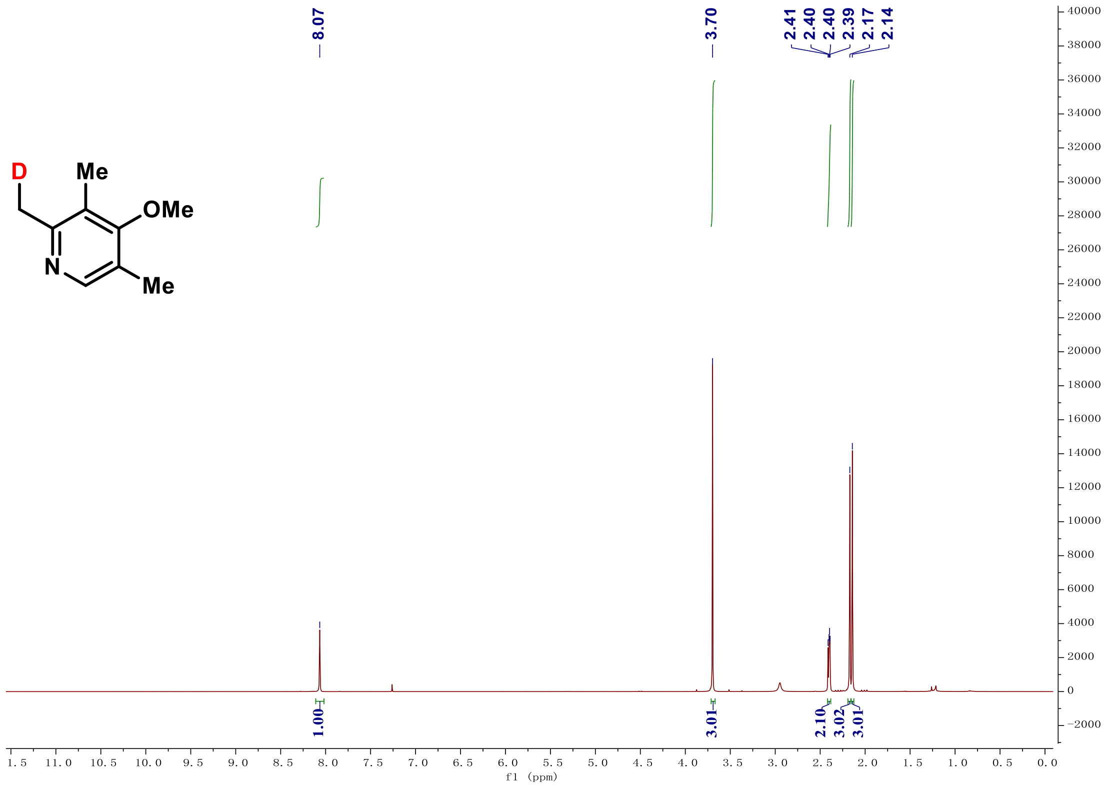


$^1$**H NMR** (400 MHz, $CDCl_3$) Spectrum of Compound **2g**

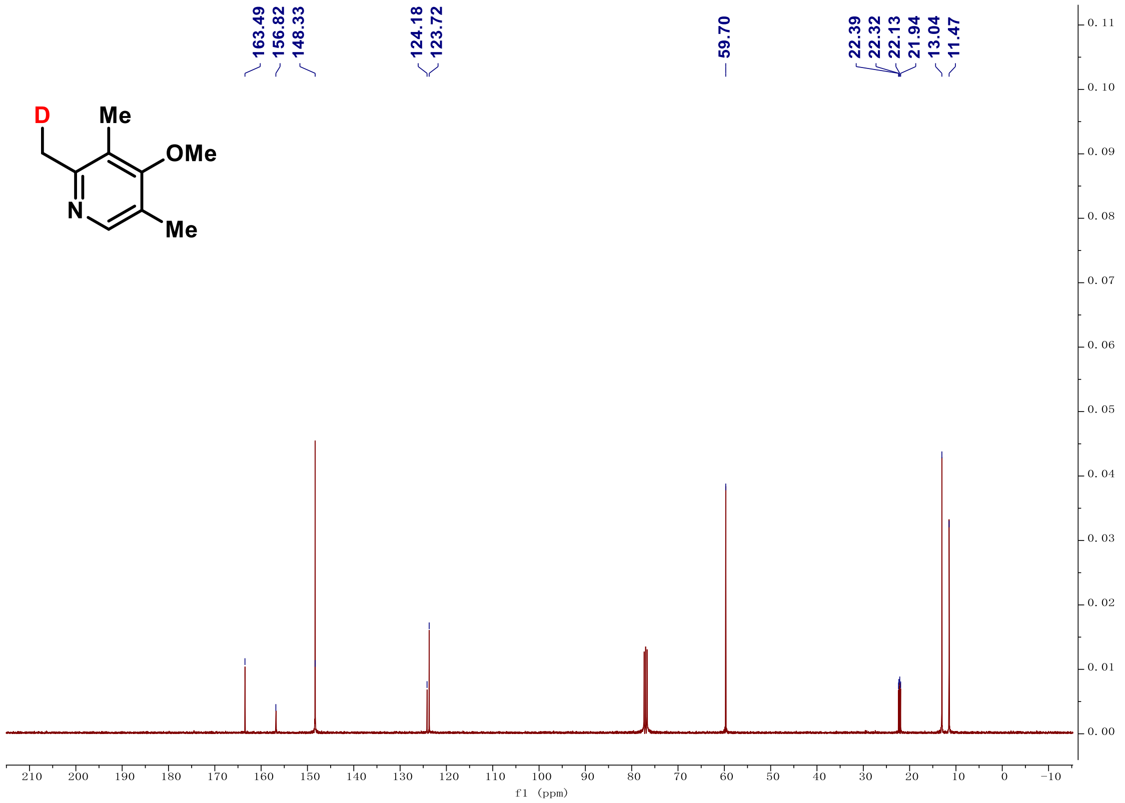


**$^{13}$C NMR** (100 MHz, $CDCl_3$) Spectrum of Compound **2g**

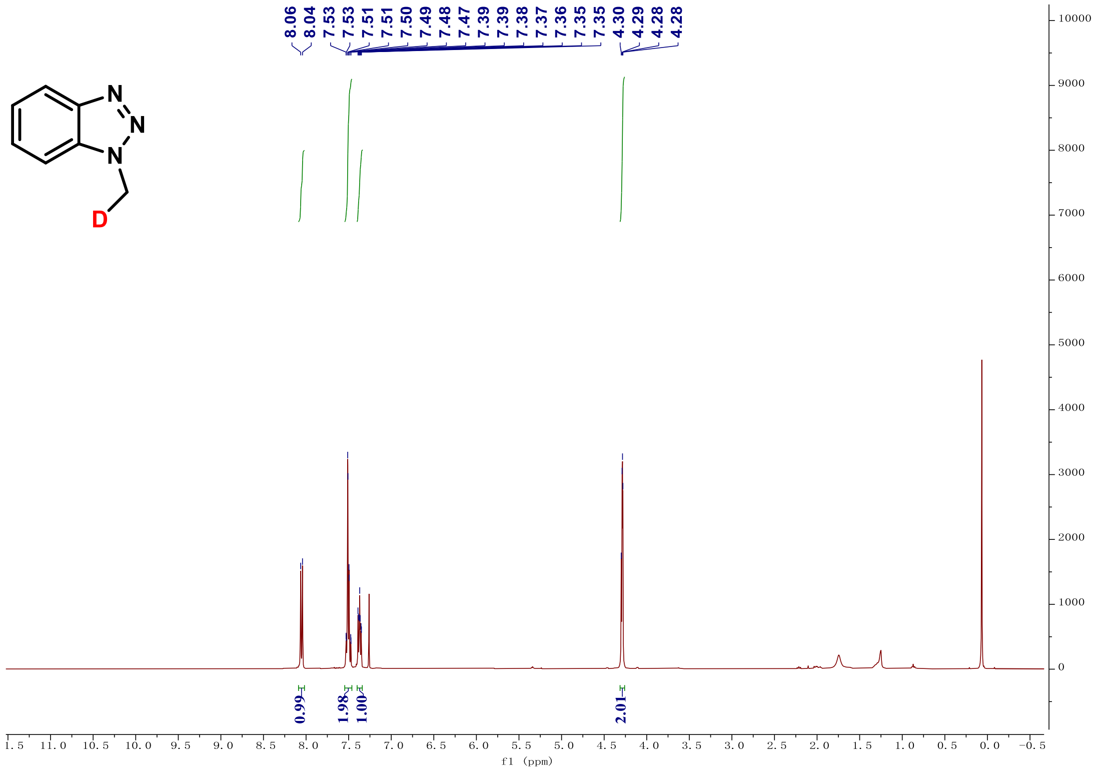


**$^{1}$H NMR** (400 MHz, $CDCl_3$) Spectrum of Compound **2h**

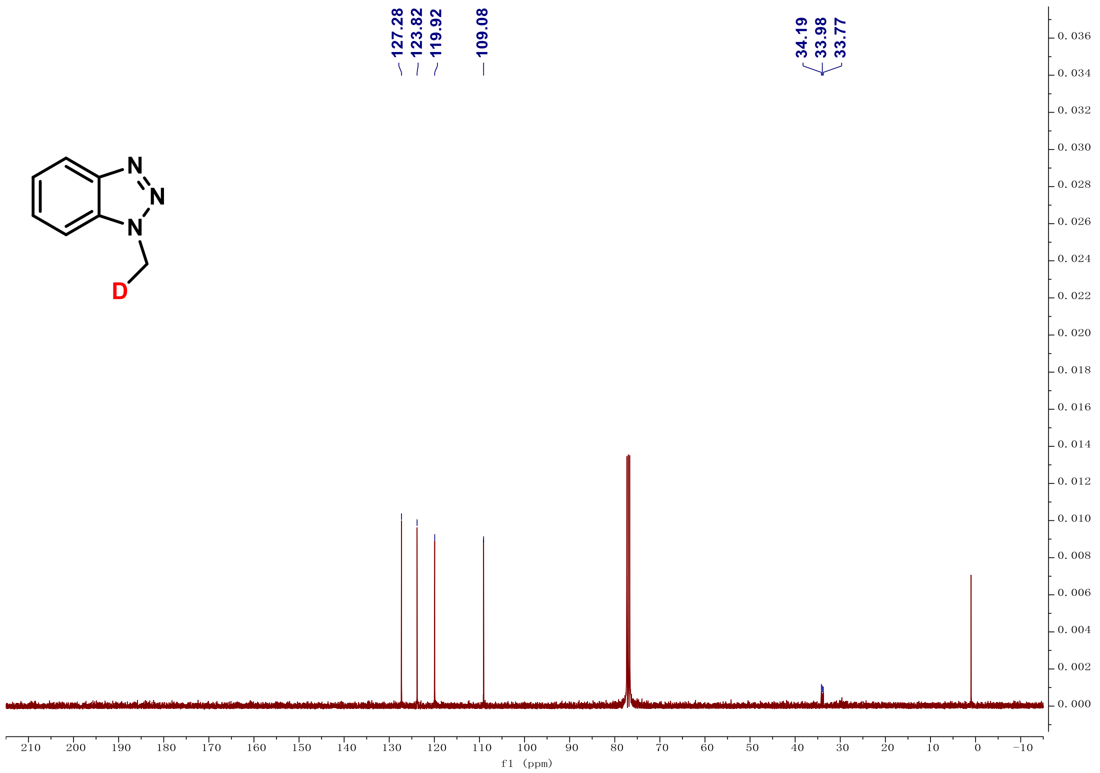

**$^{13}$C NMR** (100 MHz, $CDCl_3$) Spectrum of Compound **2h**

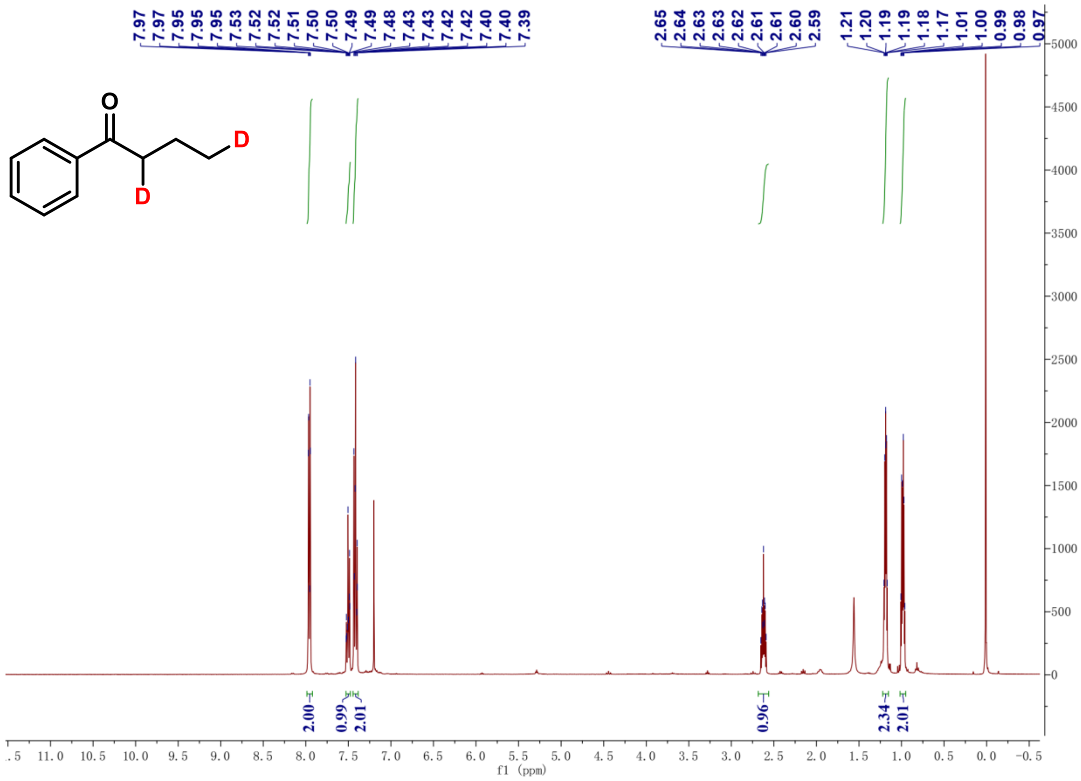

**$^{1}$H NMR** (400 MHz, $CDCl_3$) Spectrum of Compound **2i**

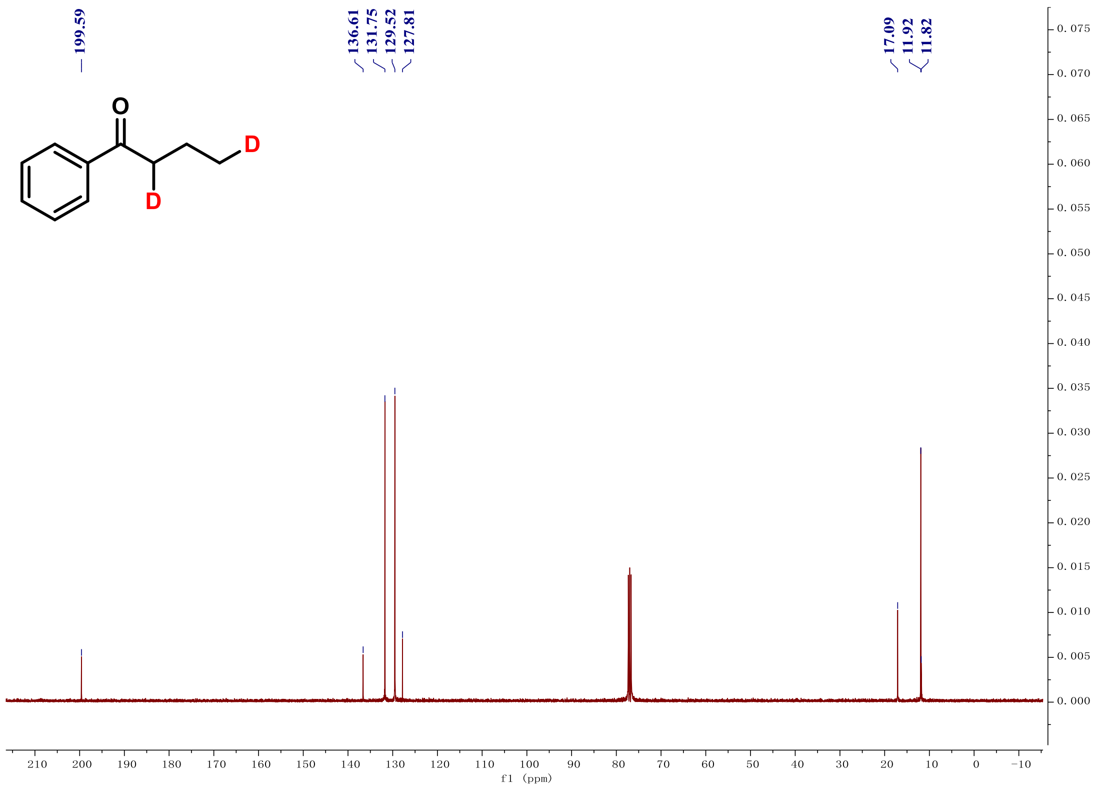


**$^{13}$C NMR** (100 MHz, $CDCl_3$) Spectrum of Compound **2i**

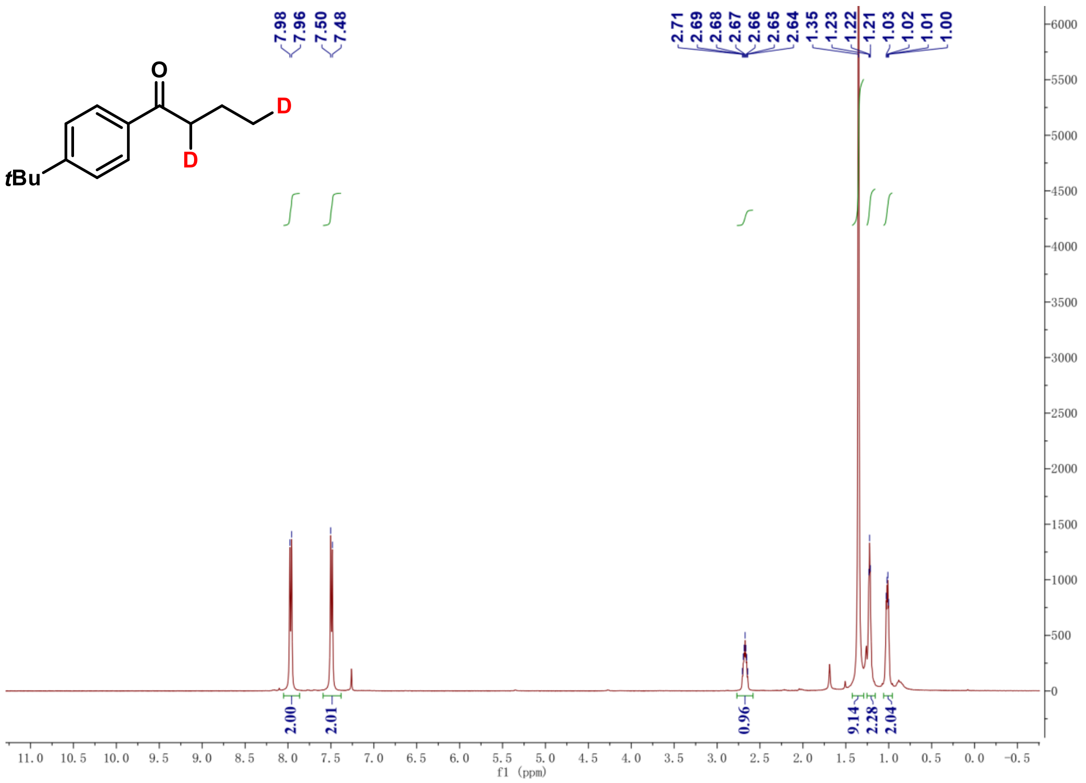


**$^{1}$H NMR** (400 MHz, $CDCl_3$) Spectrum of Compound **2j**

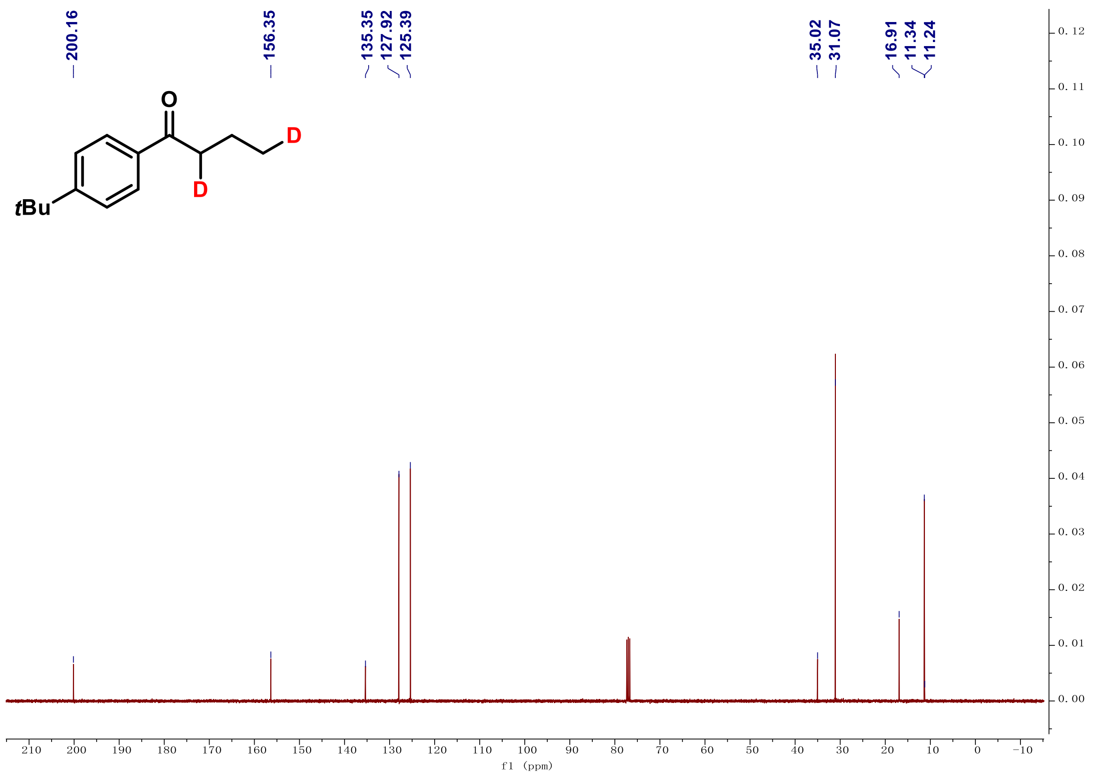


$^{13}$**C NMR** (100 MHz, $CDCl_3$) Spectrum of Compound **2j**

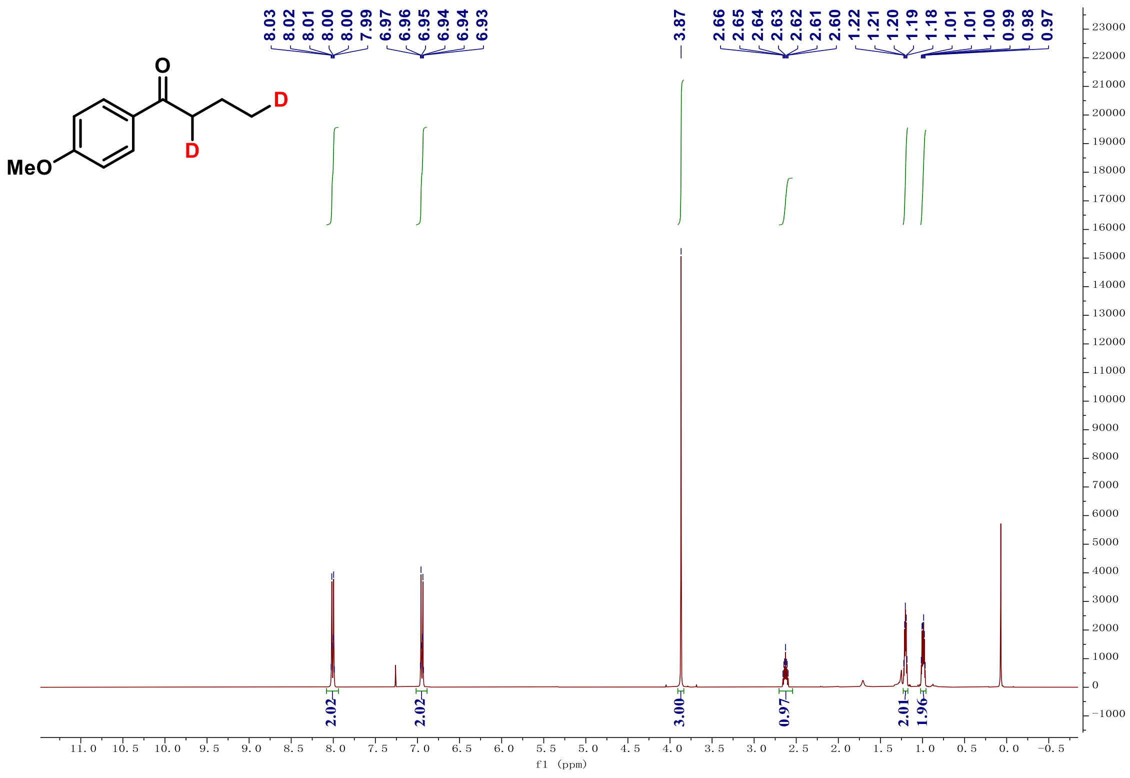


$^{1}$**H NMR** (400 MHz, $CDCl_3$) Spectrum of Compound **2k**

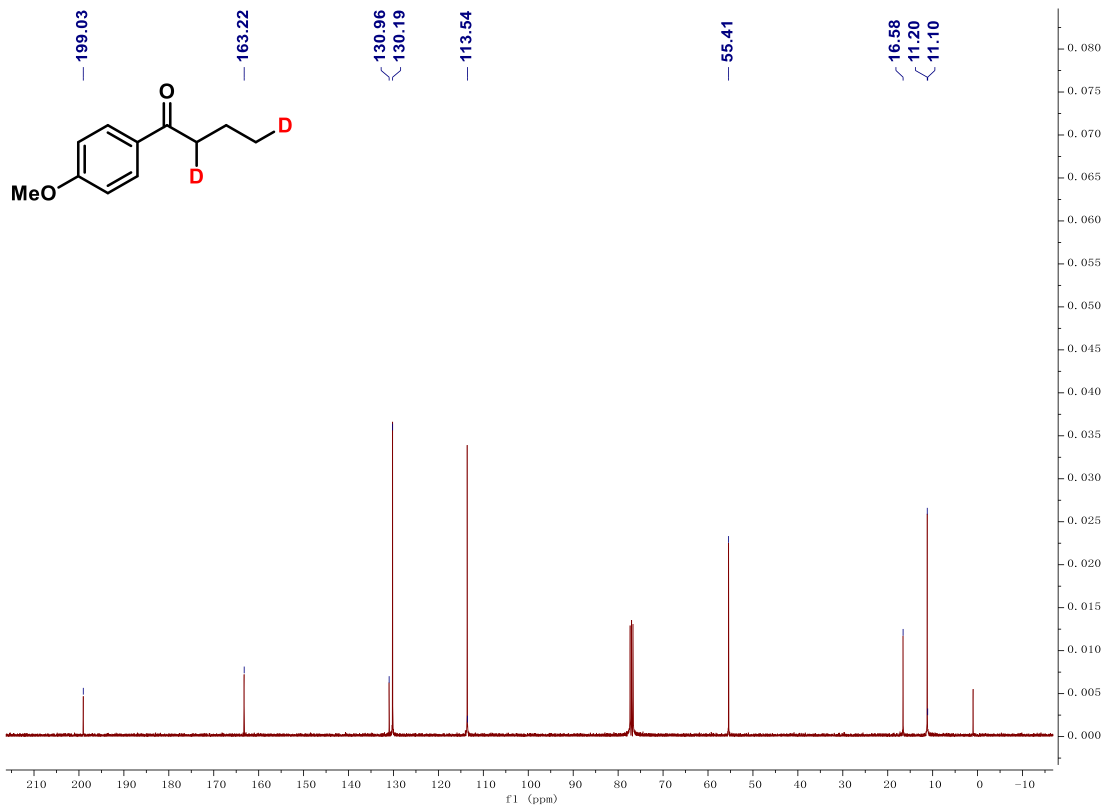


$^{13}$C **NMR** (100 MHz, $CDCl_3$) Spectrum of Compound **2k**

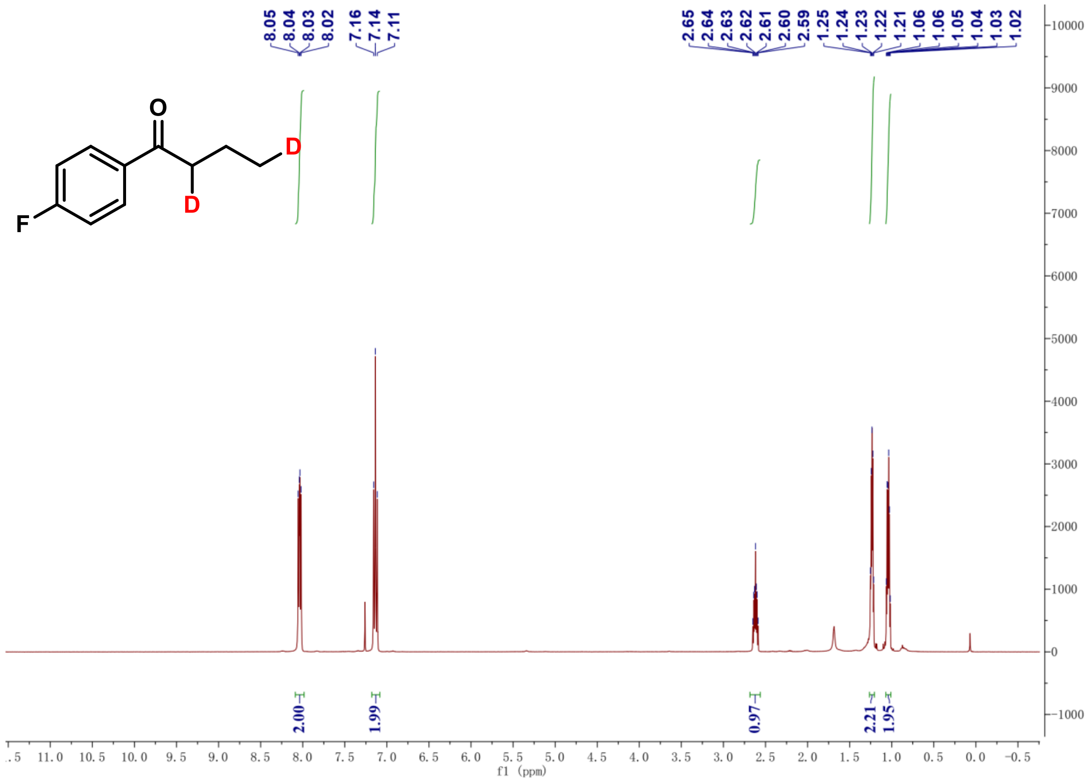


$^{1}$H **NMR** (400 MHz, $CDCl_3$) Spectrum of Compound **2l**

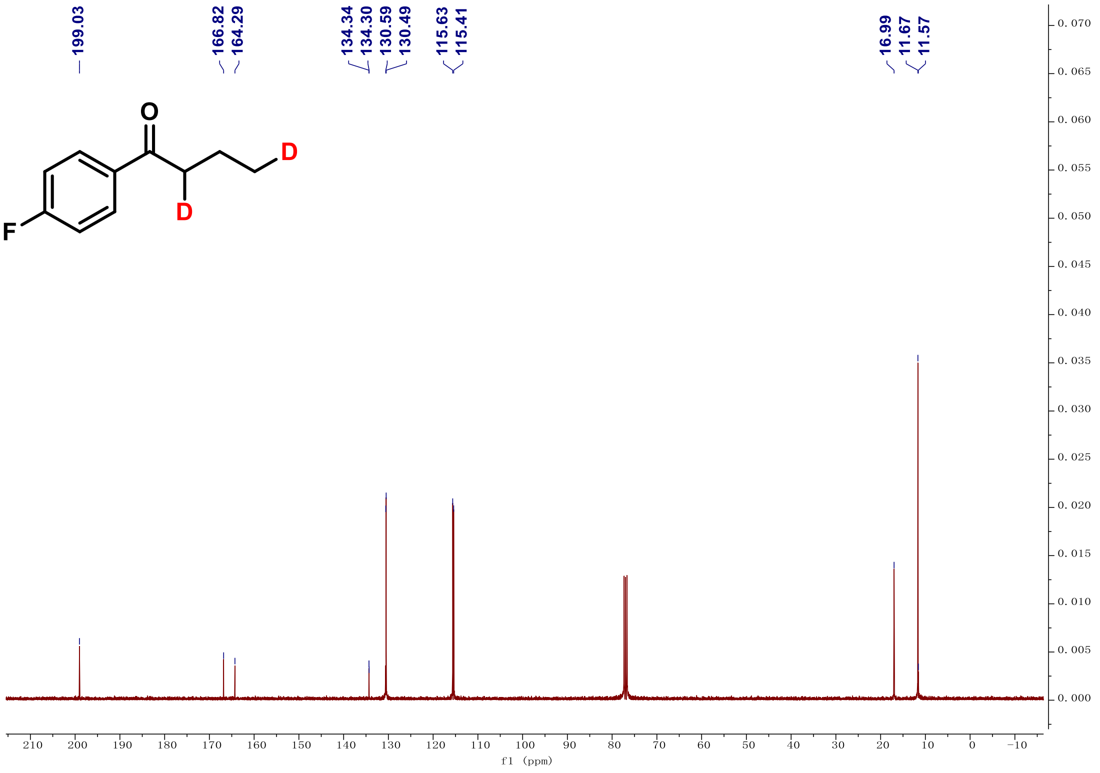


$^{13}$**C NMR** (100 MHz, $CDCl_3$) Spectrum of Compound **2l**

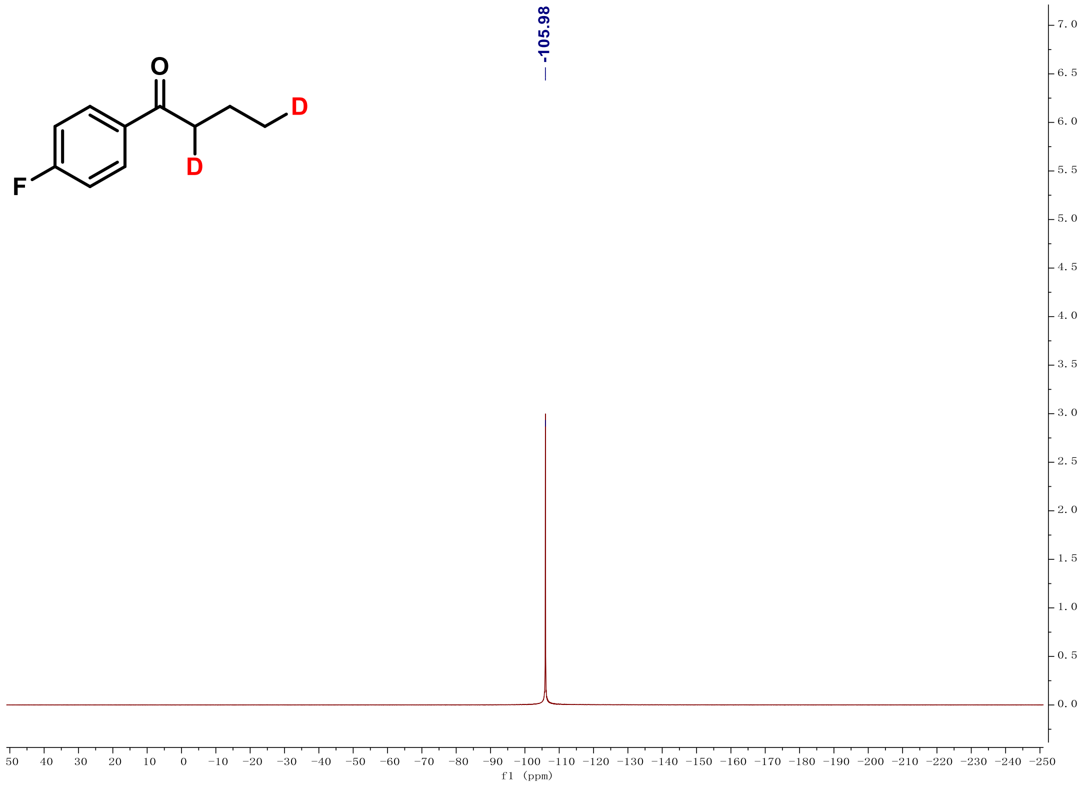


$^{19}$**F NMR** (376 MHz, $CDCl_3$) Spectrum of Compound **2l**

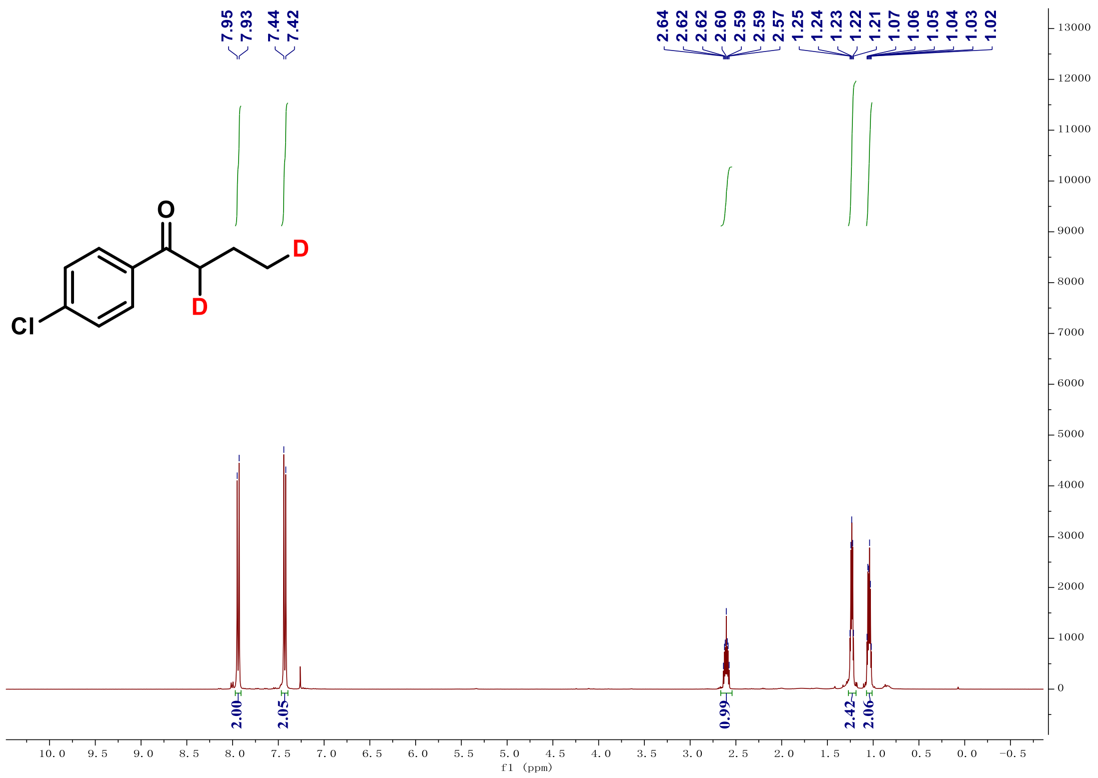


$^{1}$**H NMR** (400 MHz, $CDCl_3$) Spectrum of Compound **2m**

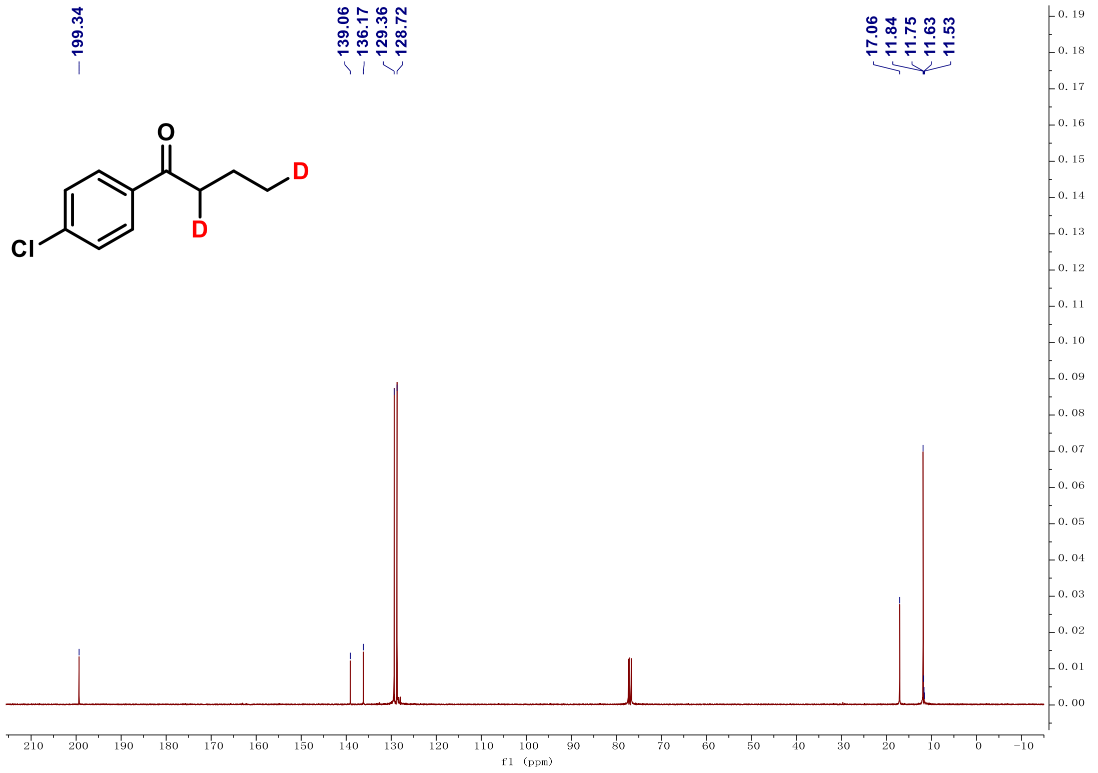


$^{13}$**C NMR** (100 MHz, $CDCl_3$) Spectrum of Compound **2m**

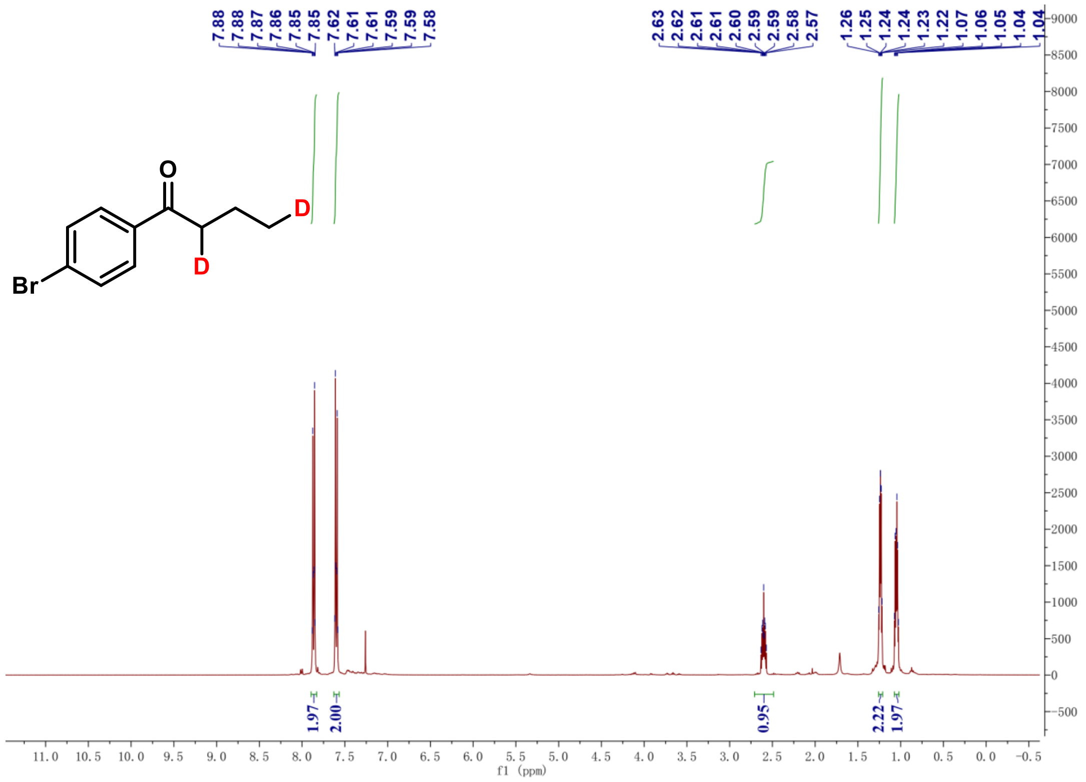


$^{1}$**H NMR** (400 MHz, $CDCl_3$) Spectrum of Compound **2n**

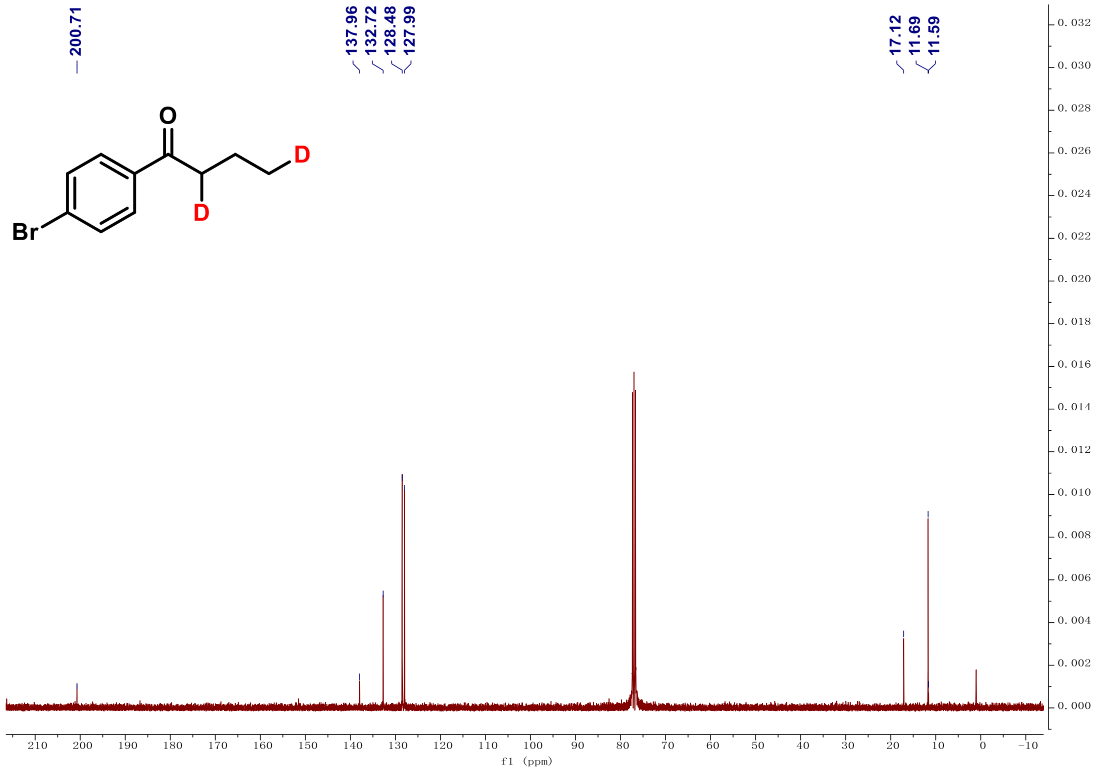


$^{13}$**C NMR** (100 MHz, $CDCl_3$) Spectrum of Compound **2n**

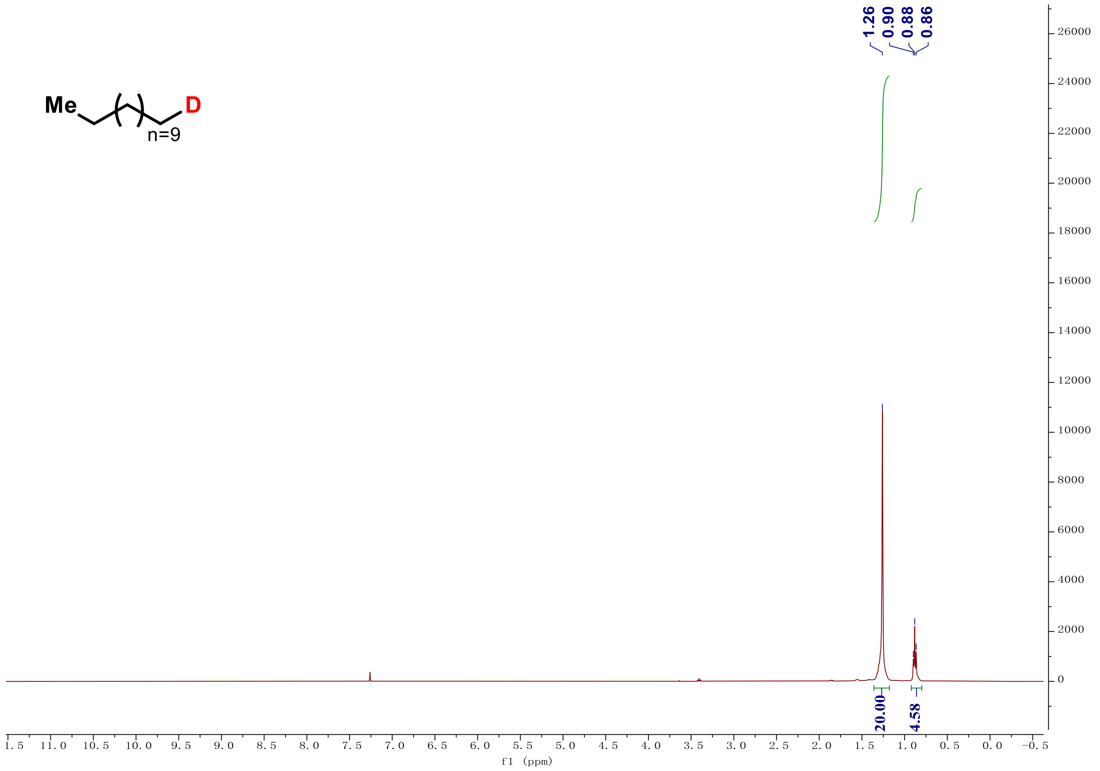


**$^{1}$H NMR** (400 MHz, $CDCl_3$) Spectrum of Compound **2o**

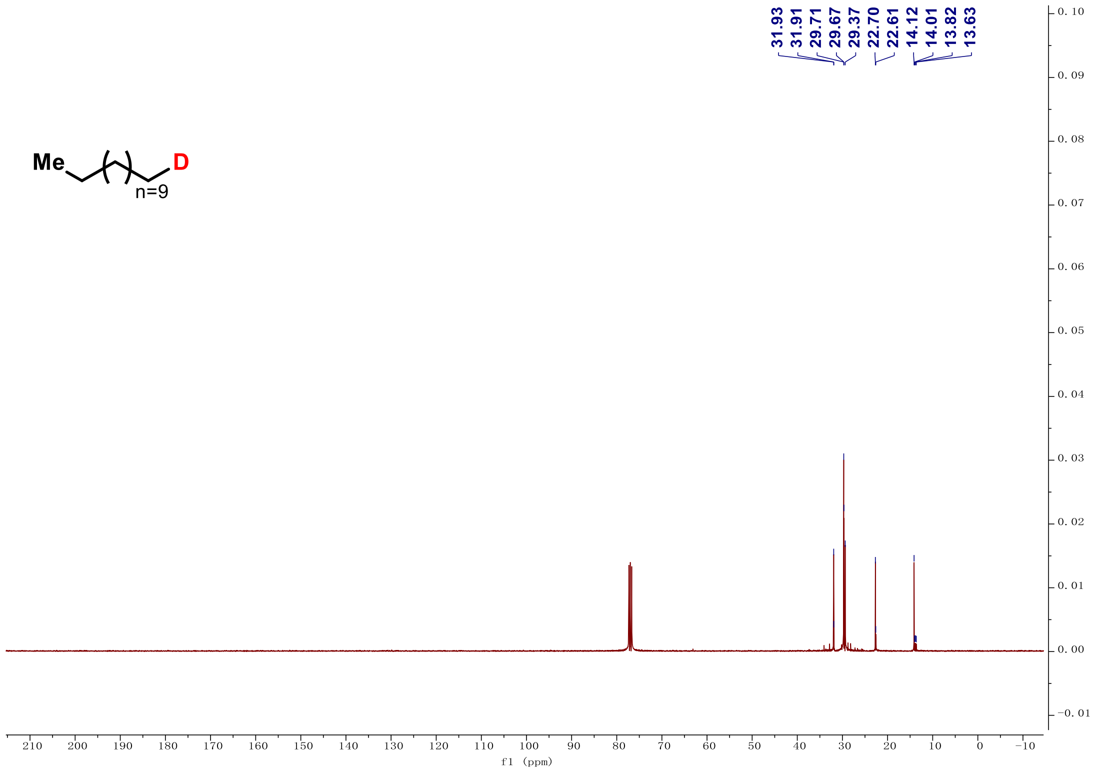


**$^{13}$C NMR** (100 MHz, $CDCl_3$) Spectrum of Compound **2o**

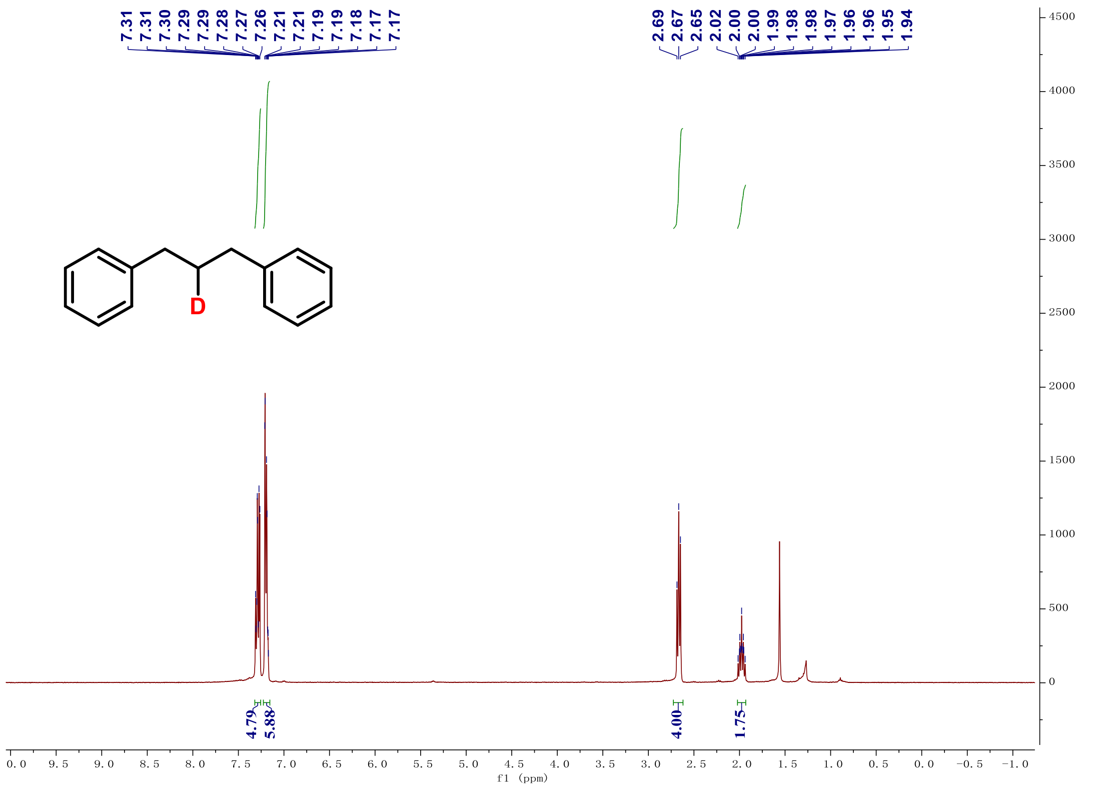


$^{1}$**H NMR** (400 MHz, $CDCl_3$) Spectrum of Compound **2p**

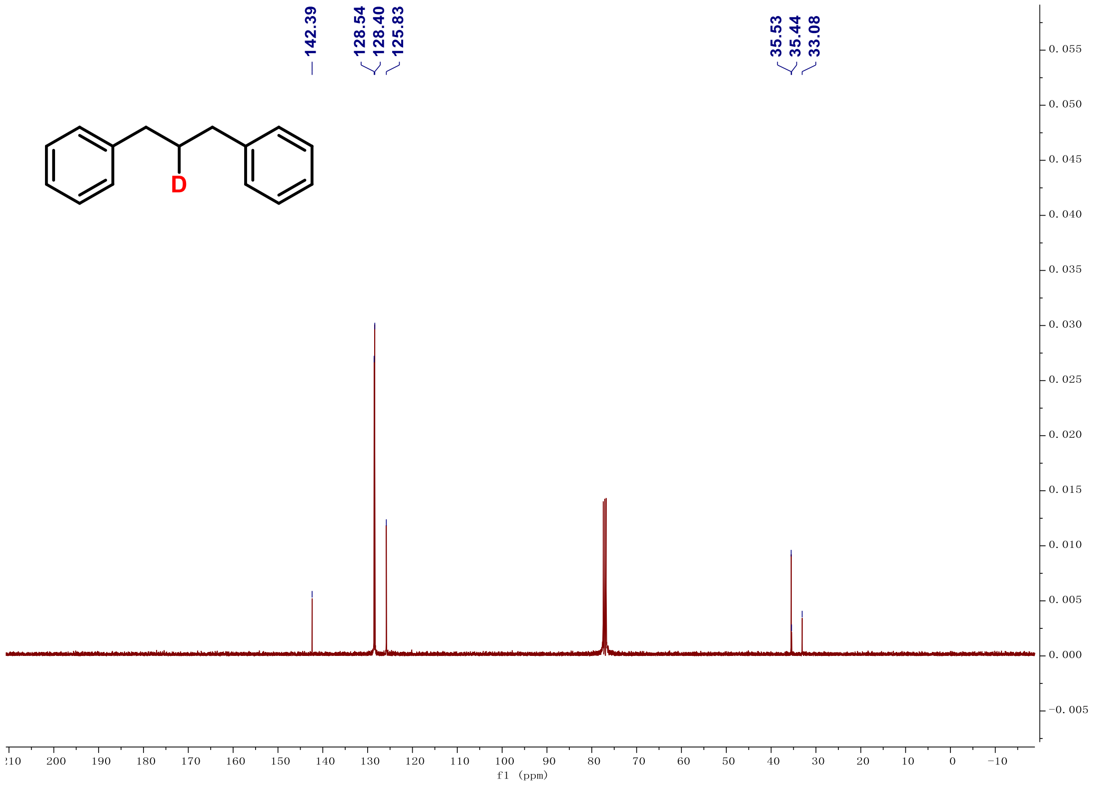


$^{13}$**C NMR** (100 MHz, $CDCl_3$) Spectrum of Compound **2p**

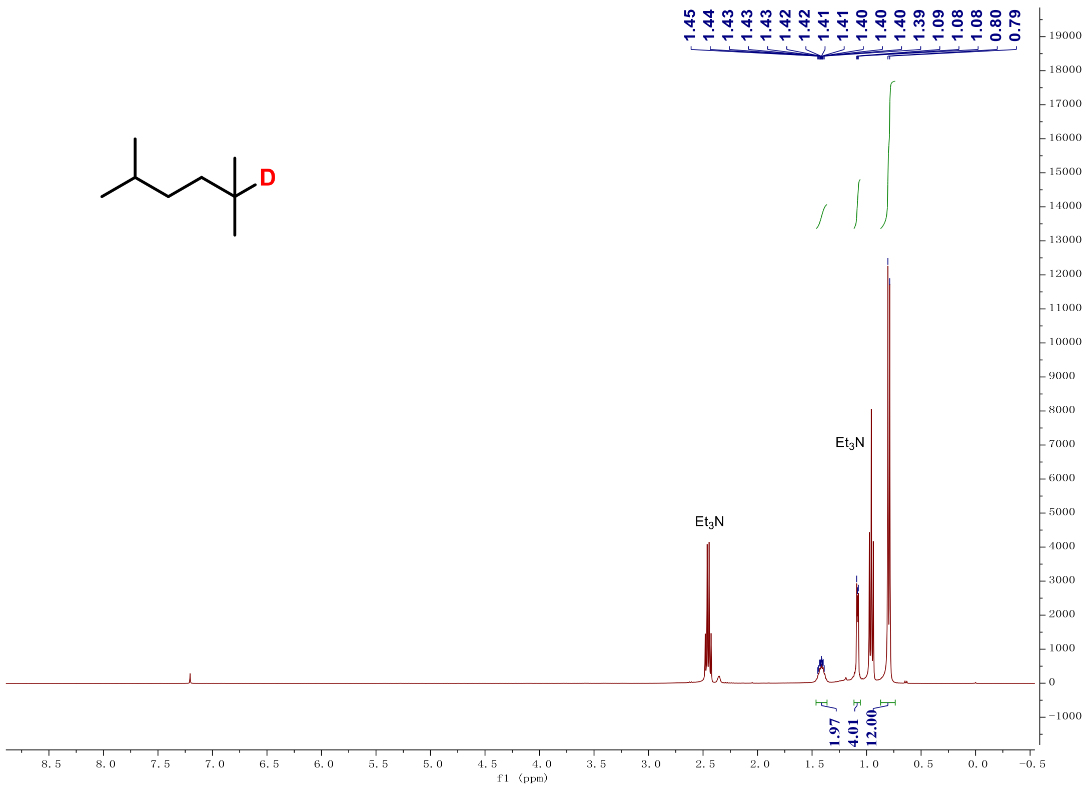


$^{1}$**H NMR (400 MHz, CDCl$_3$)** Spectrum of Compound **2q** (crude mixture obtained after the reaction)

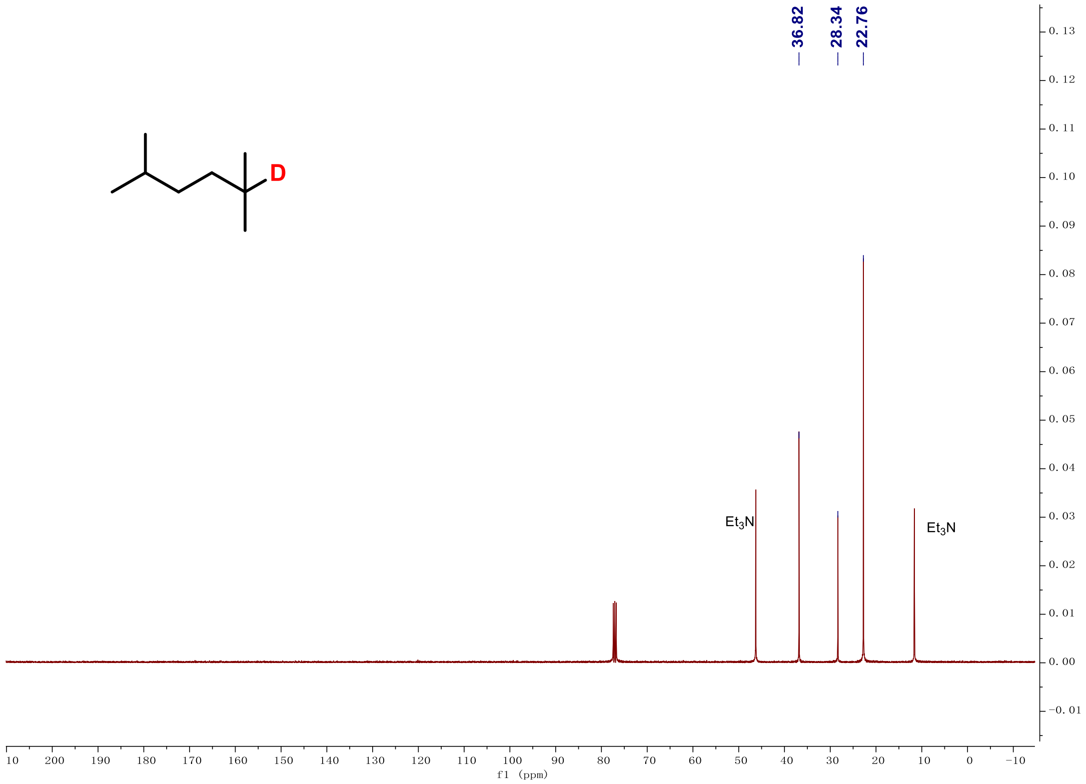


$^{13}$**C NMR (100 MHz, CDCl$_3$)** Spectrum of Compound **2q** (crude mixture obtained after the reaction)

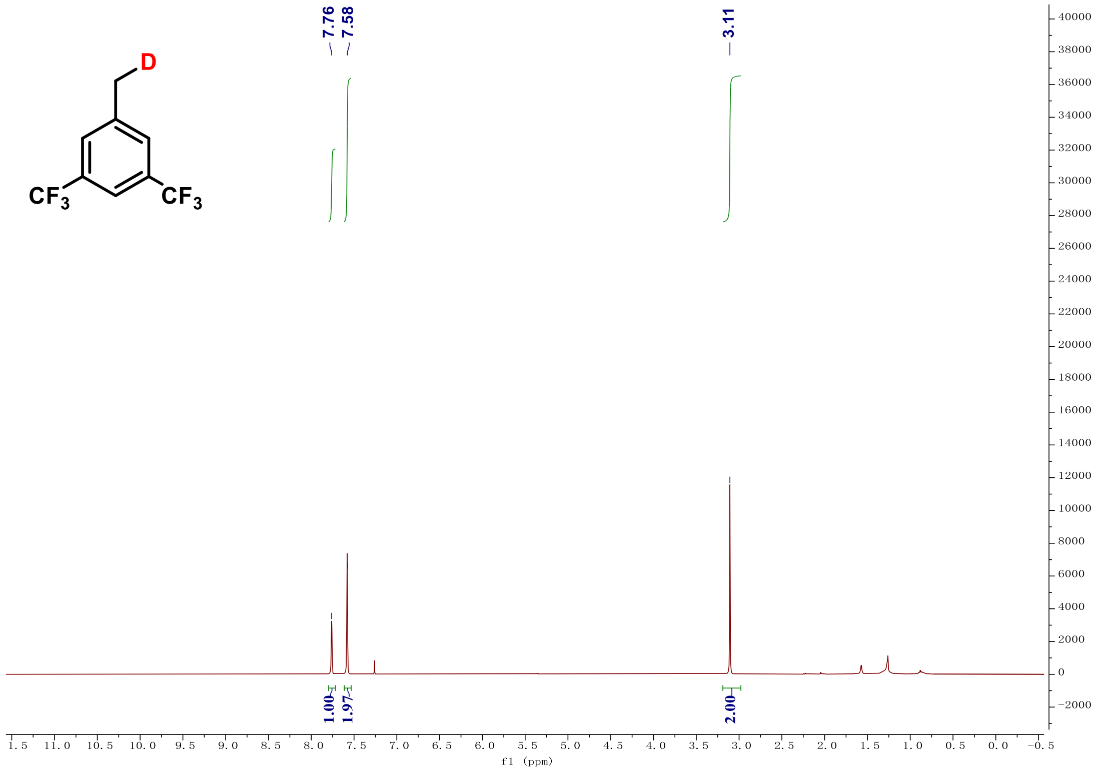


**$^{1}$H NMR** (400 MHz, $CDCl_3$) Spectrum of Compound **2r**

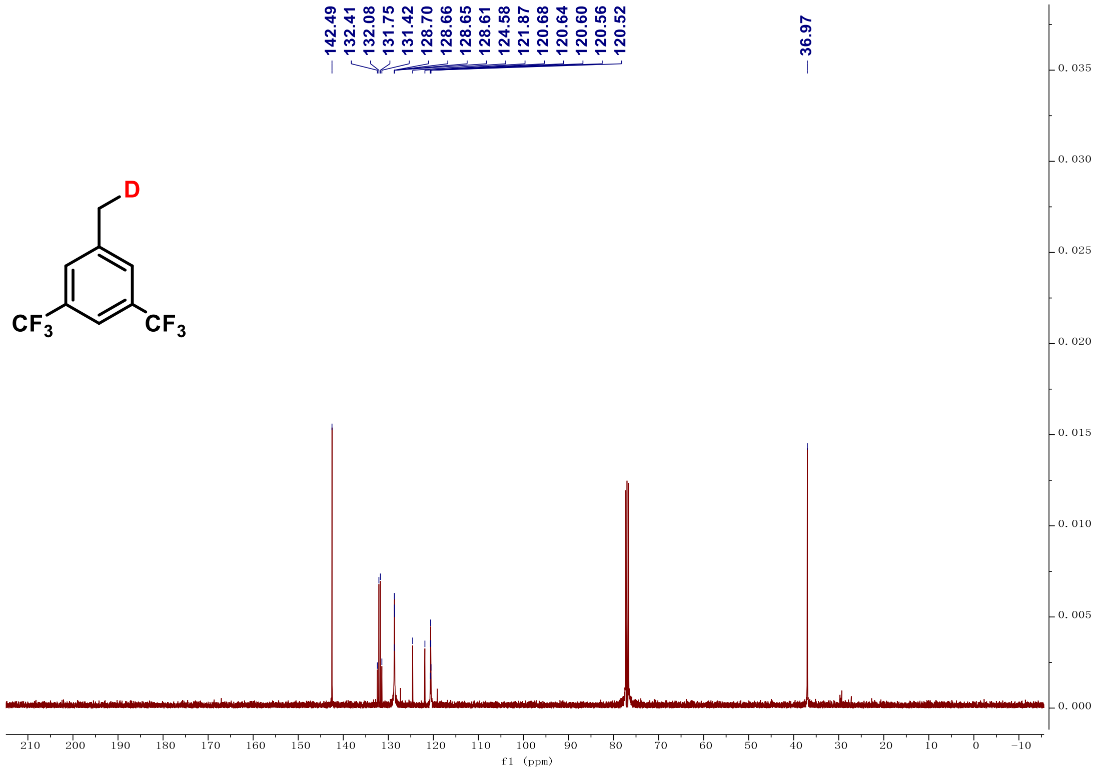


**$^{13}$C NMR** (100 MHz, $CDCl_3$) Spectrum of Compound **2r**

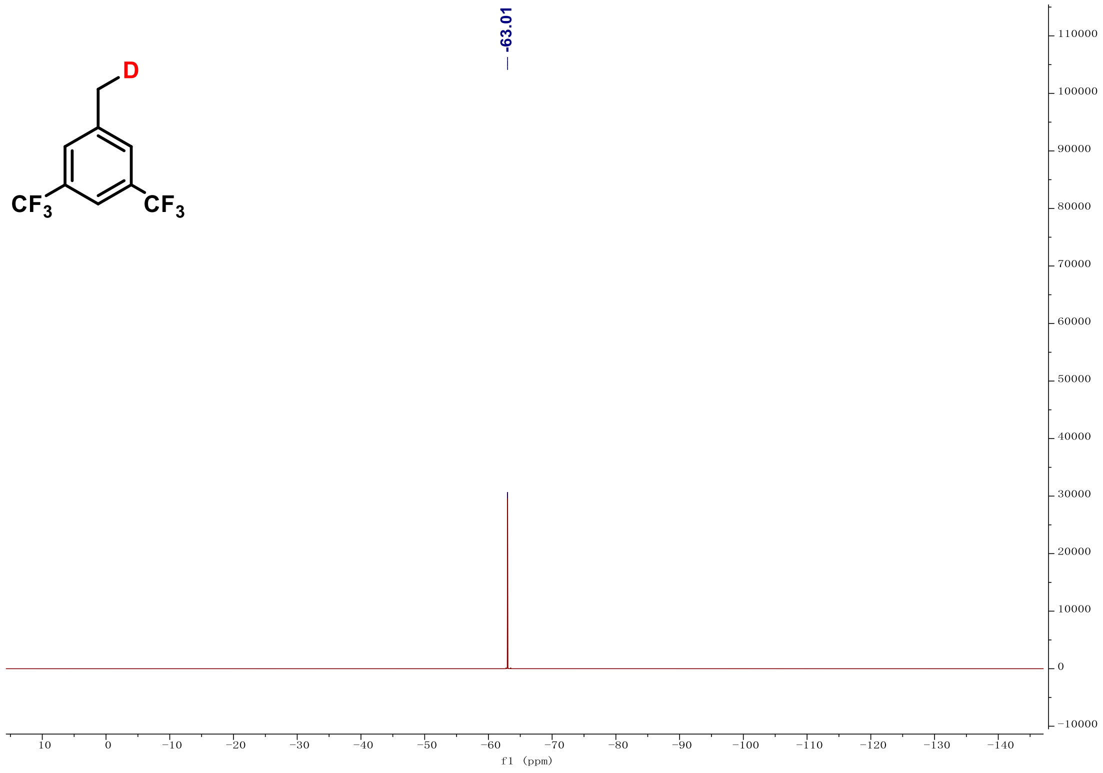


$^{19}$**F NMR** (376 MHz, $CDCl_3$) Spectrum of Compound **2r**

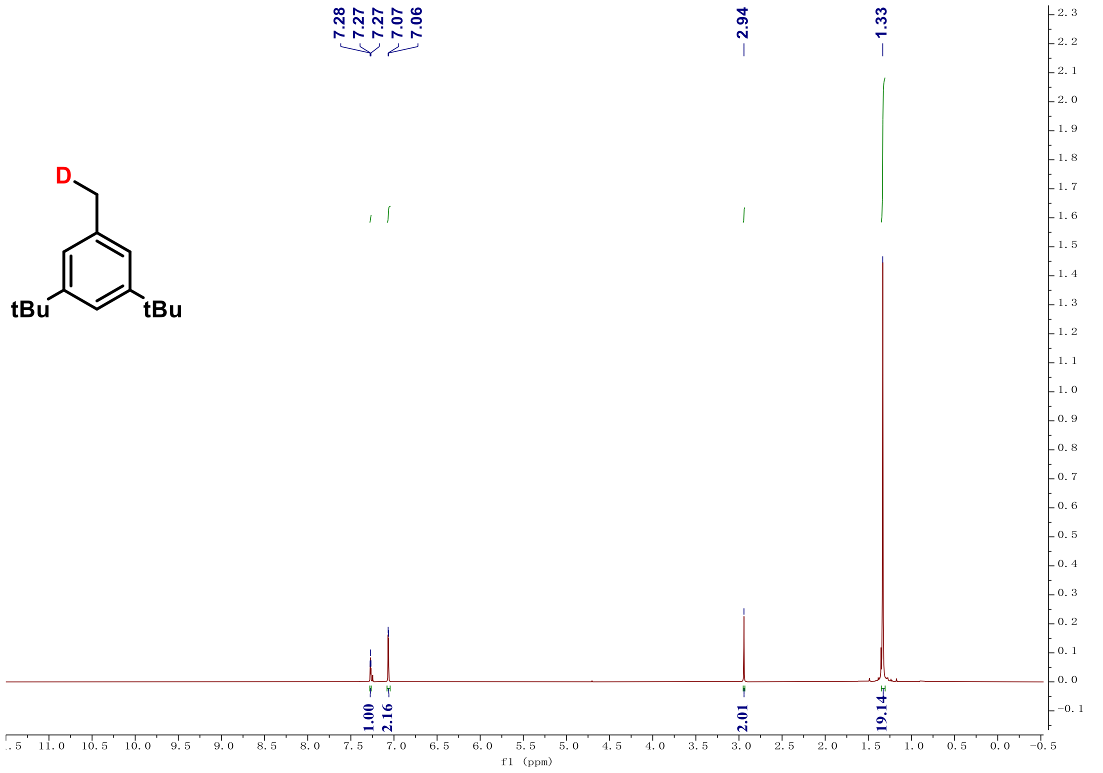


$^{1}$**H NMR** (400 MHz, $CDCl_3$) Spectrum of Compound **2s**

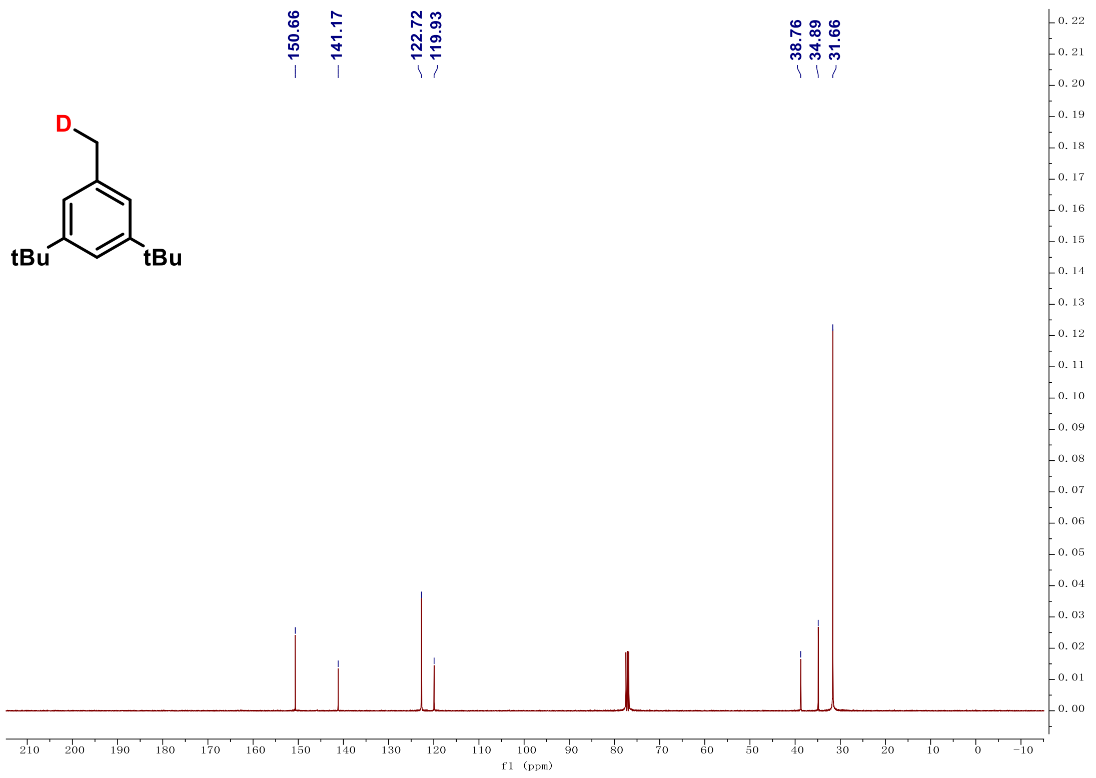


**$^{13}$C NMR** (100 MHz, CDCl₃) Spectrum of Compound **2s**

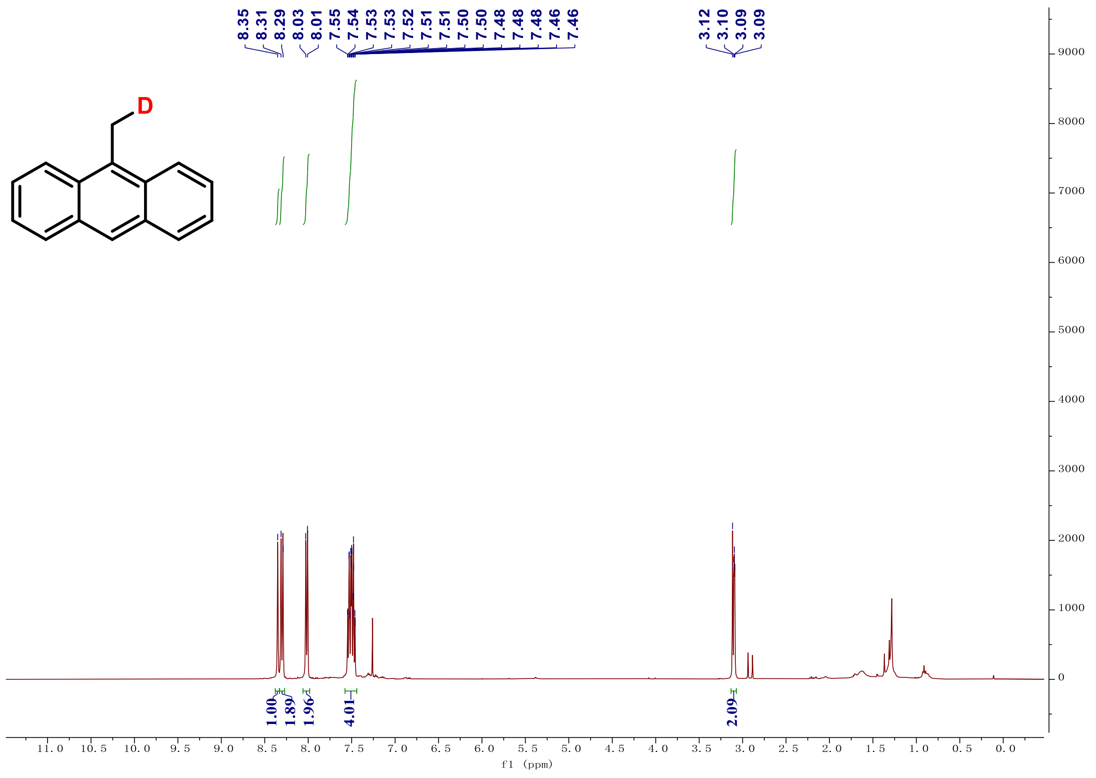


**$^{1}$H NMR** (400 MHz, CDCl₃) Spectrum of Compound **2t**

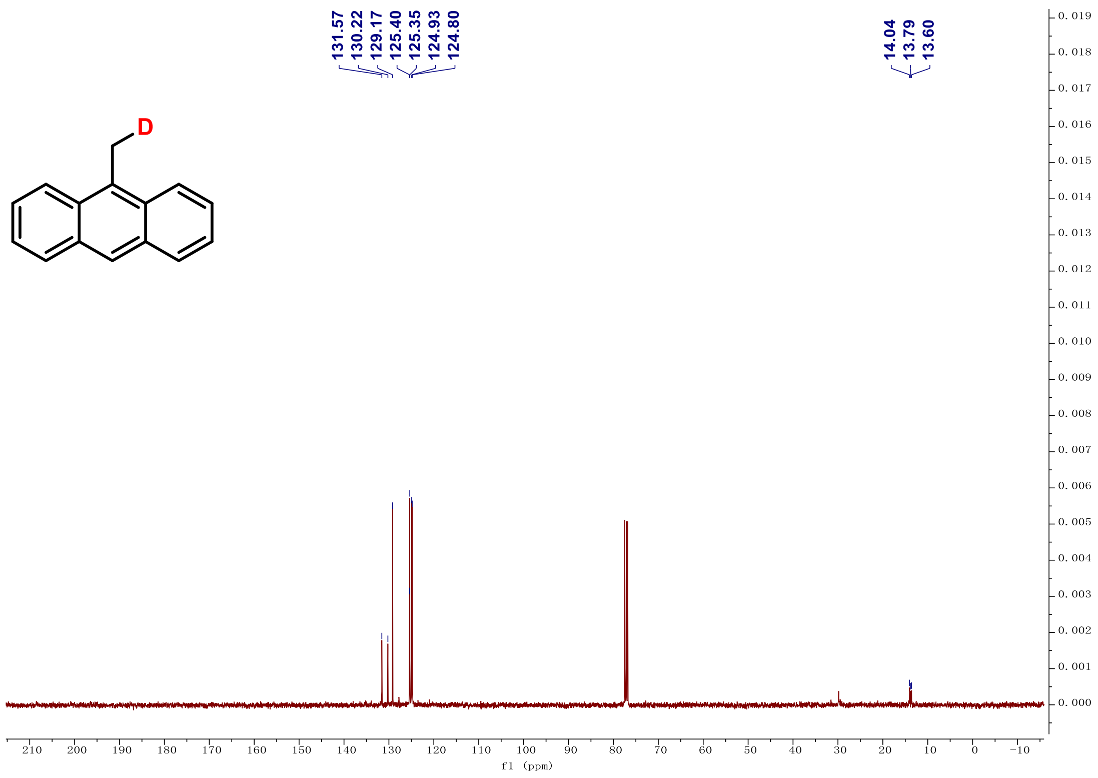


**$^{13}$C NMR** (100 MHz, $CDCl_3$) Spectrum of Compound **2t**

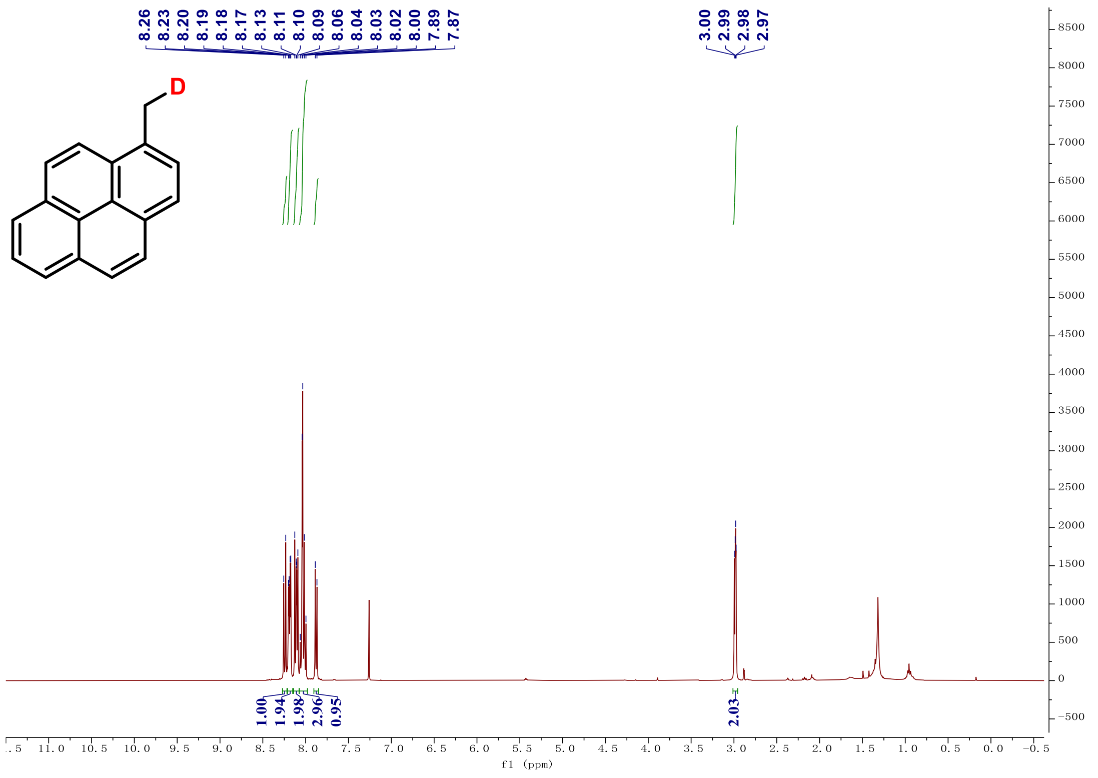


**$^{1}$H NMR** (400 MHz, $CDCl_3$) Spectrum of Compound **2u**

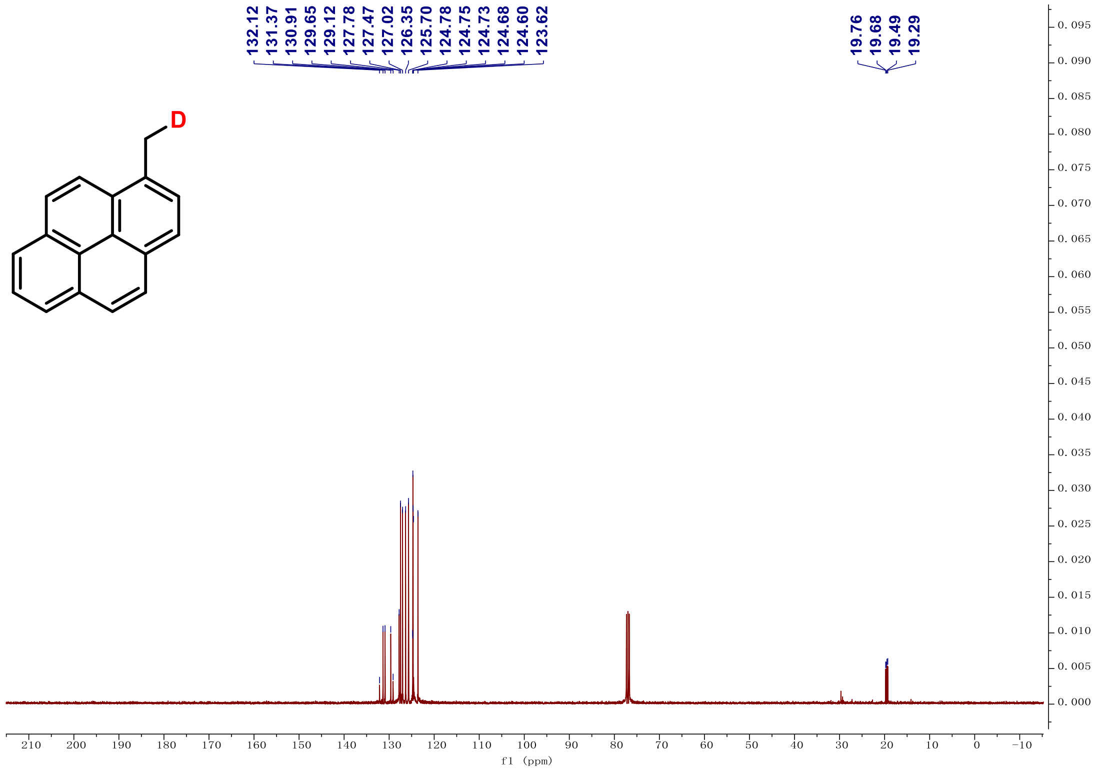


$^{13}$C NMR (100 MHz, $CDCl_3$) Spectrum of Compound **2u**

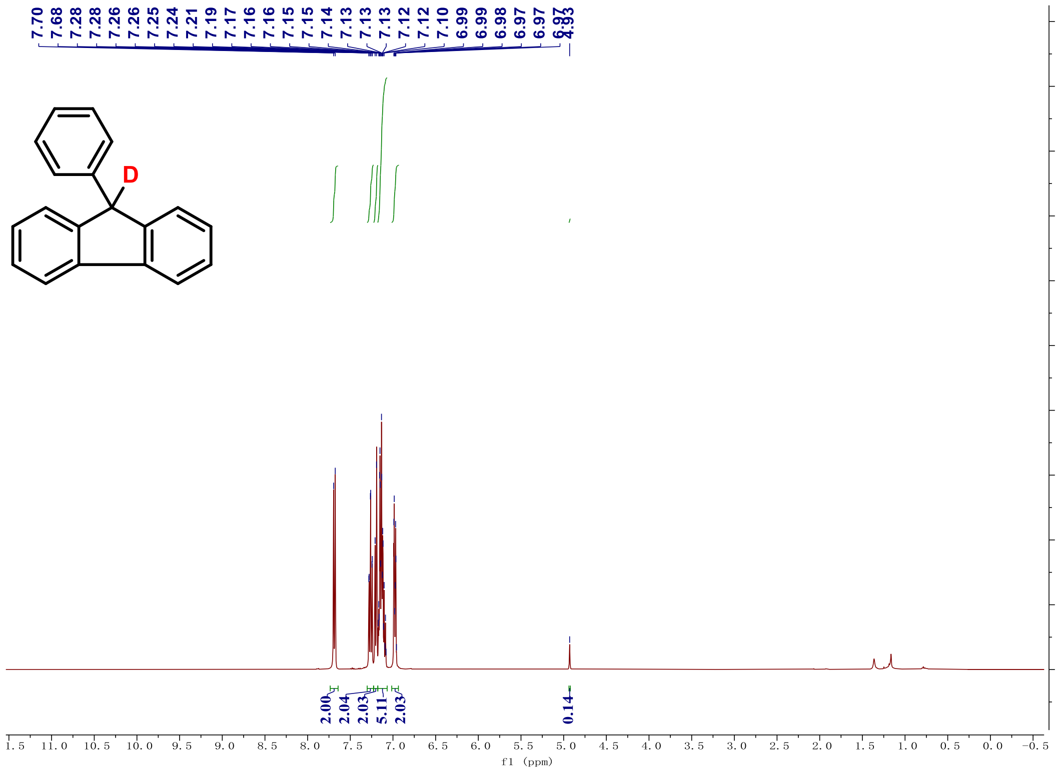


$^{1}$H NMR (400 MHz, $CDCl_3$) Spectrum of Compound **2v**

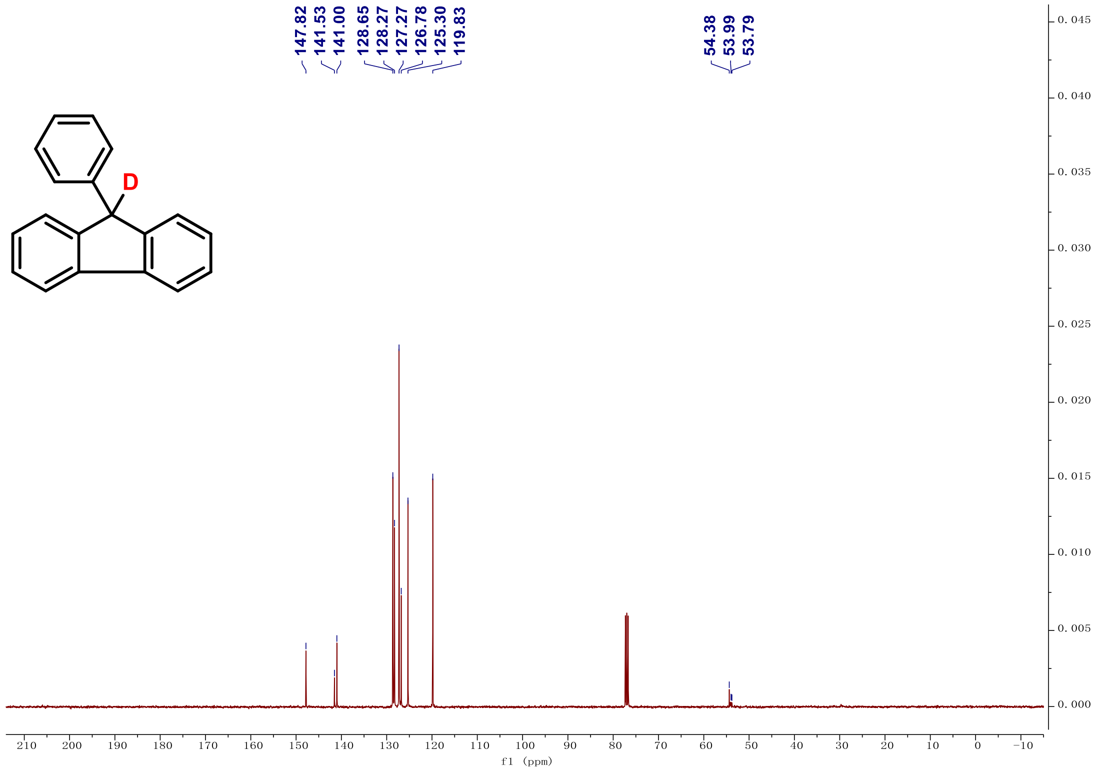


**$^{13}$C NMR** (100 MHz, $CDCl_3$) Spectrum of Compound **2v**

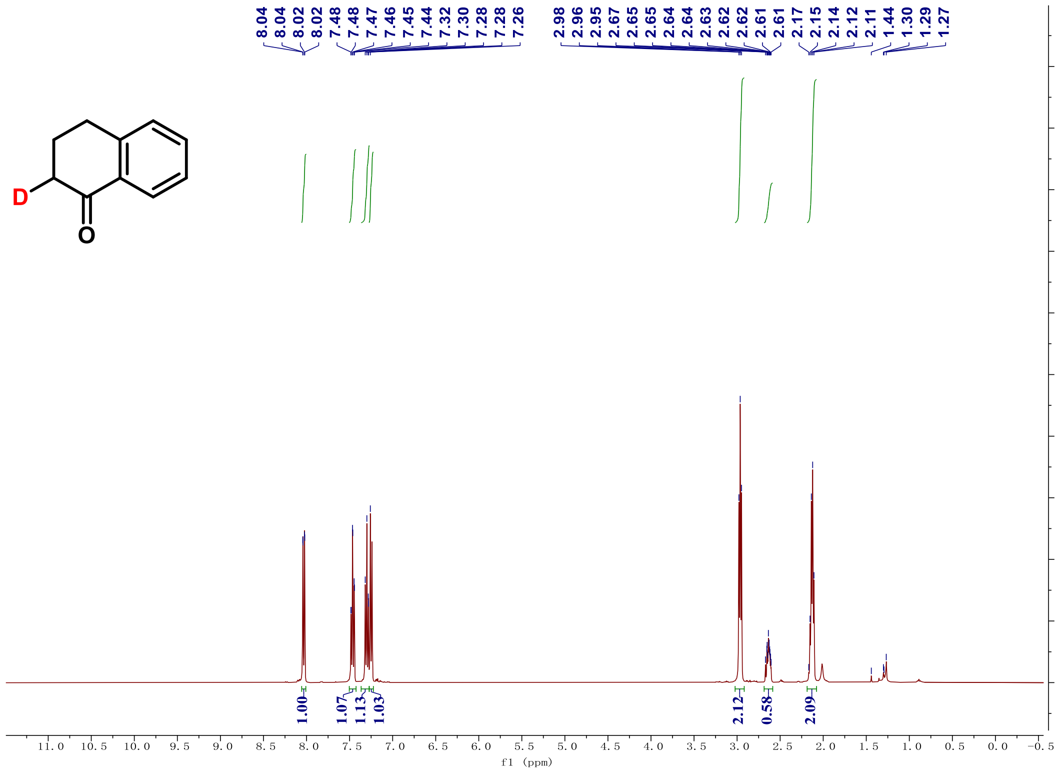


**$^{1}$H NMR** (400 MHz, $CDCl_3$) Spectrum of Compound **2w**

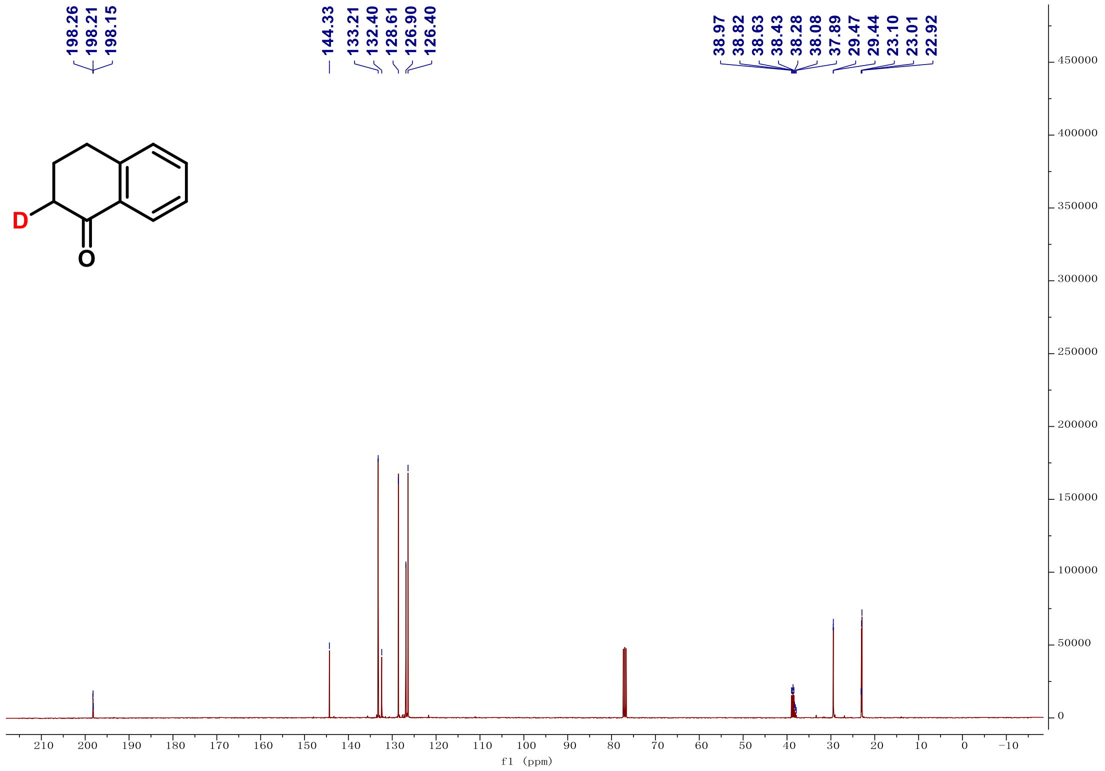

**$^{13}$C NMR** (100 MHz, CDCl$_3$) Spectrum of Compound **2w**

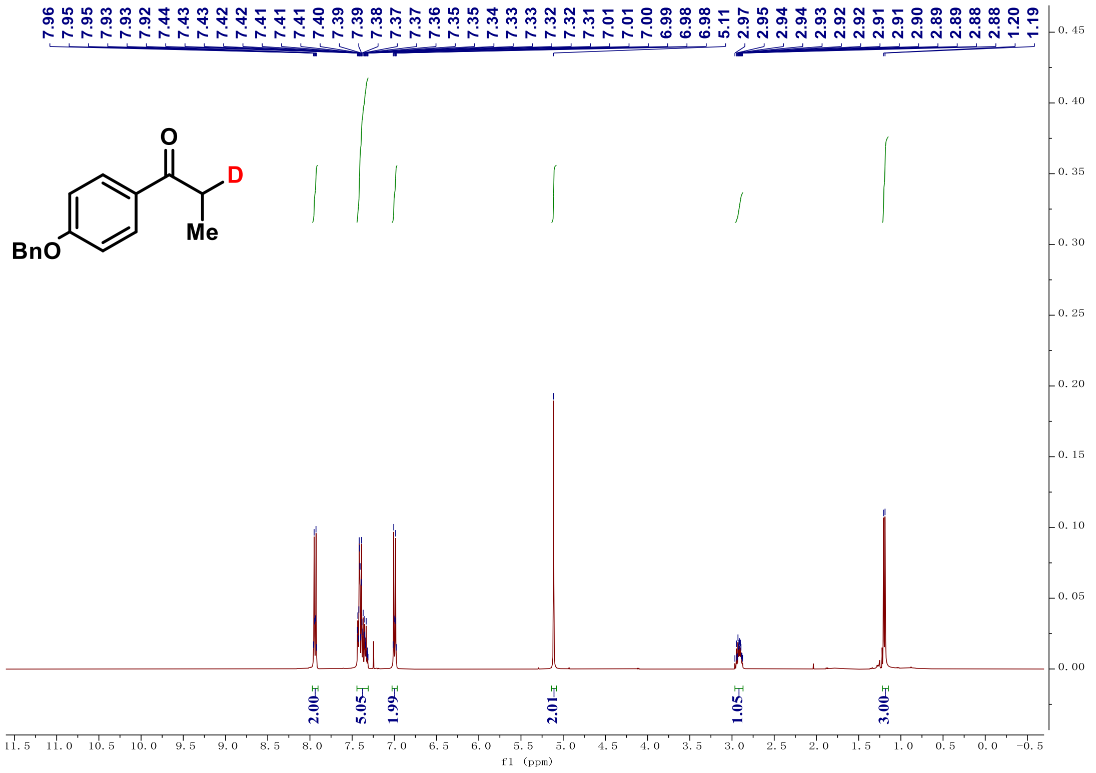

**$^{1}$H NMR** (400 MHz, CDCl$_3$) Spectrum of Compound **2x**

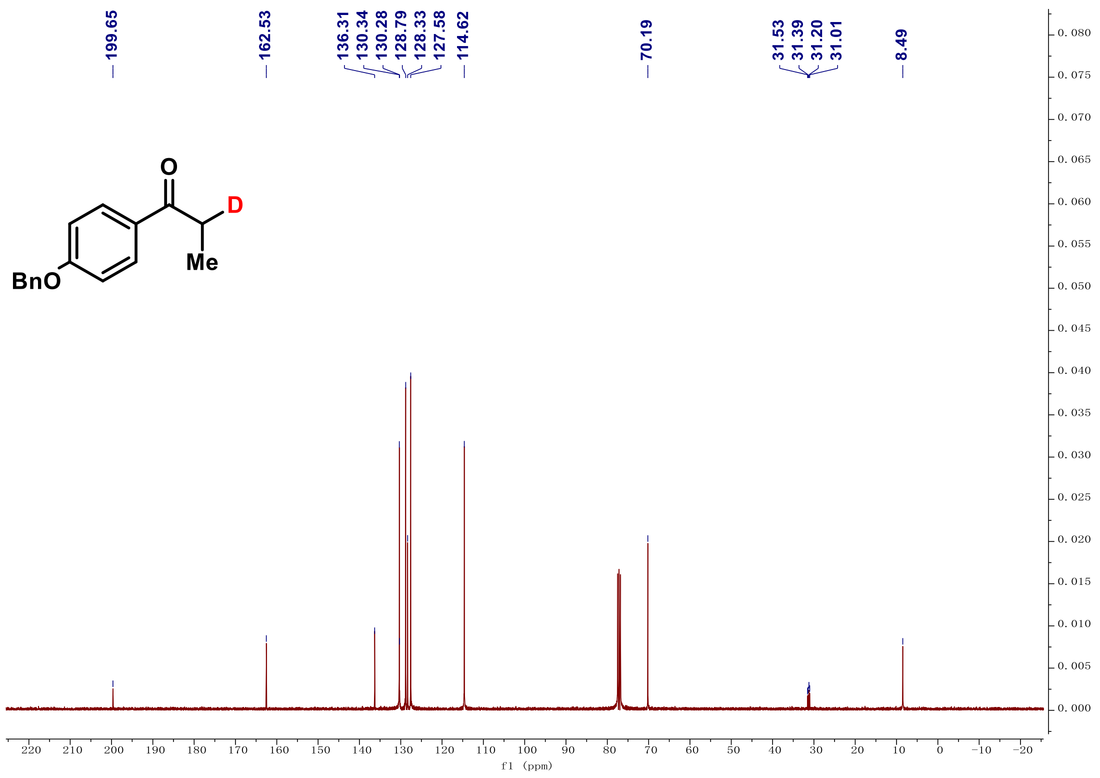


**$^{13}$C NMR** (100 MHz, $CDCl_3$) Spectrum of Compound **2x**

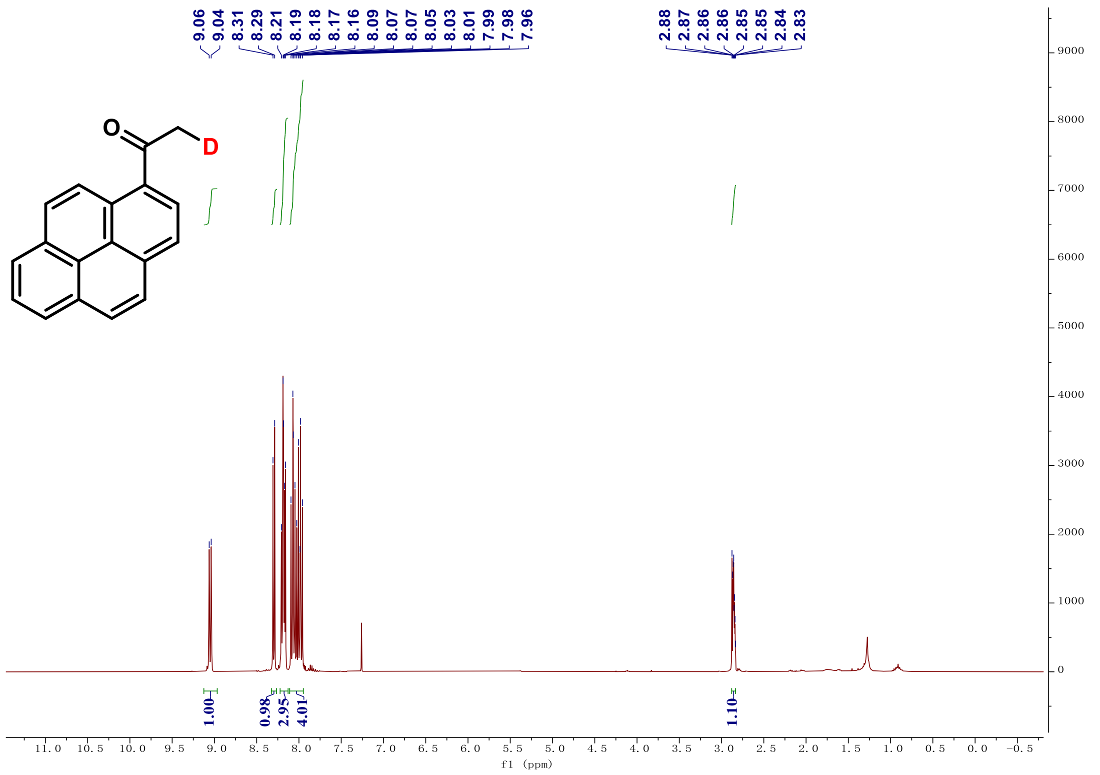


**$^{1}$H NMR** (400 MHz, $CDCl_3$) Spectrum of Compound **2y**

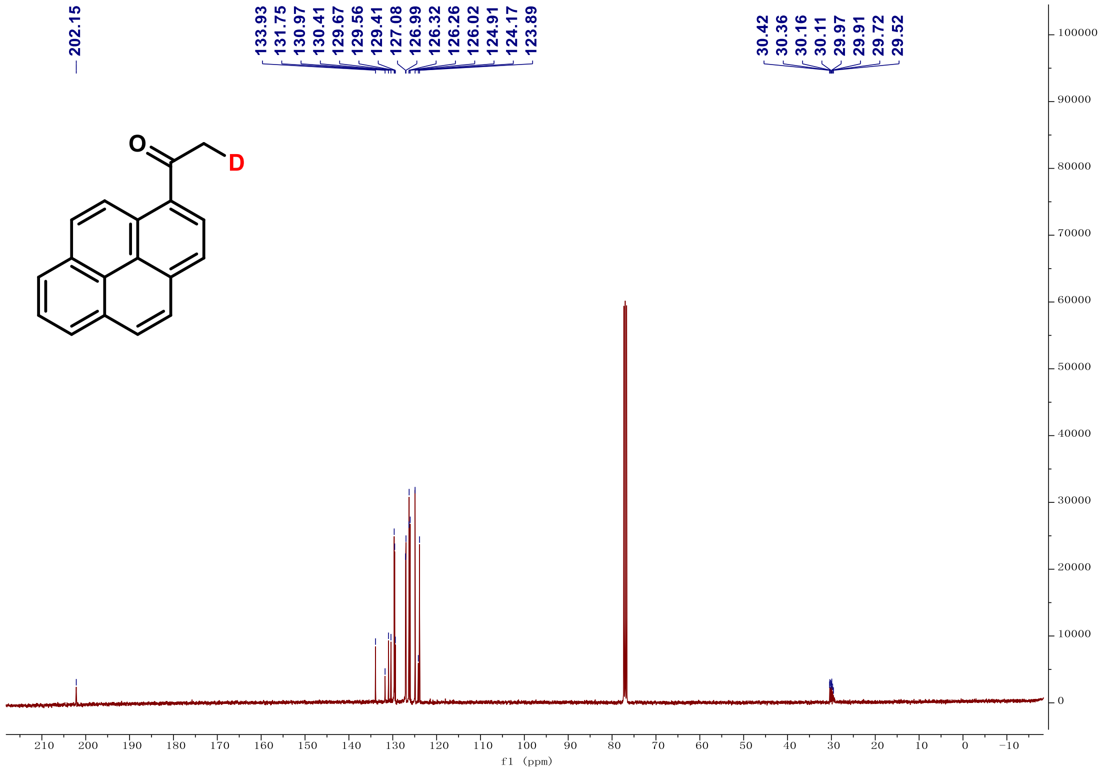


$^{13}$**C NMR** (100 MHz, $CDCl_3$) Spectrum of Compound **2y**

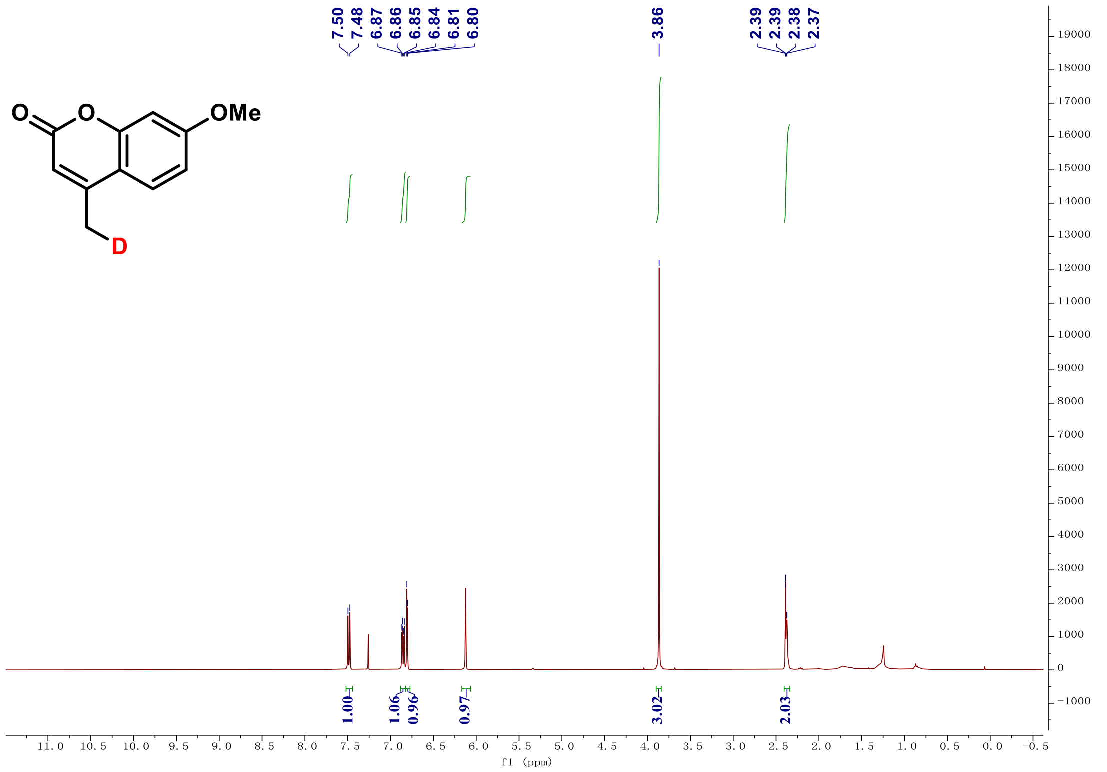


$^{1}$**H NMR** (400 MHz, $CDCl_3$) Spectrum of Compound **2z**

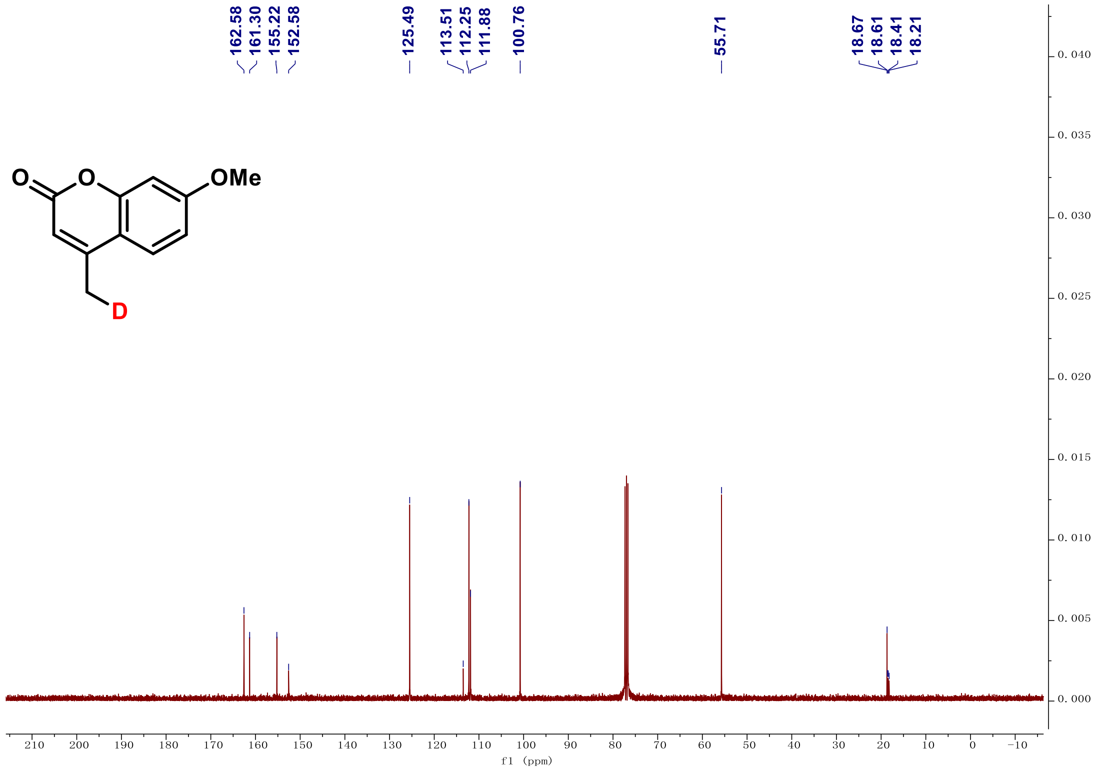


**$^{13}$C NMR** (100 MHz, CDCl$_3$) Spectrum of Compound **2z**

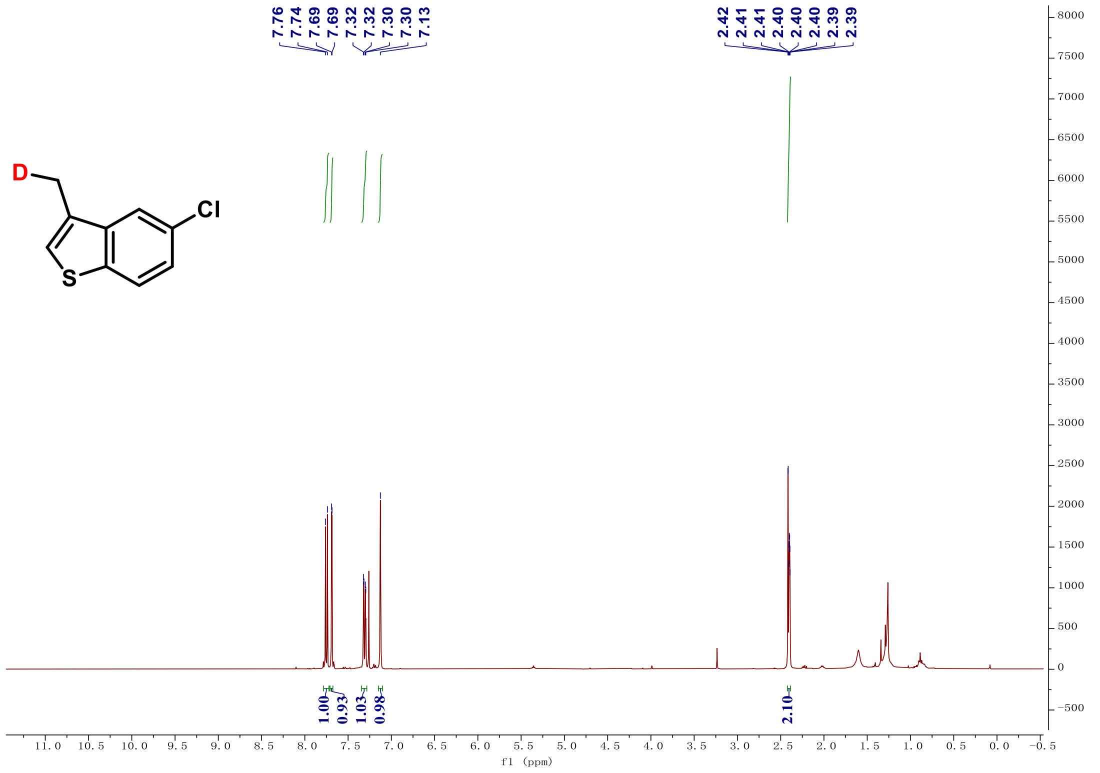


**$^{1}$H NMR** (400 MHz, CDCl$_3$) Spectrum of Compound **2aa**

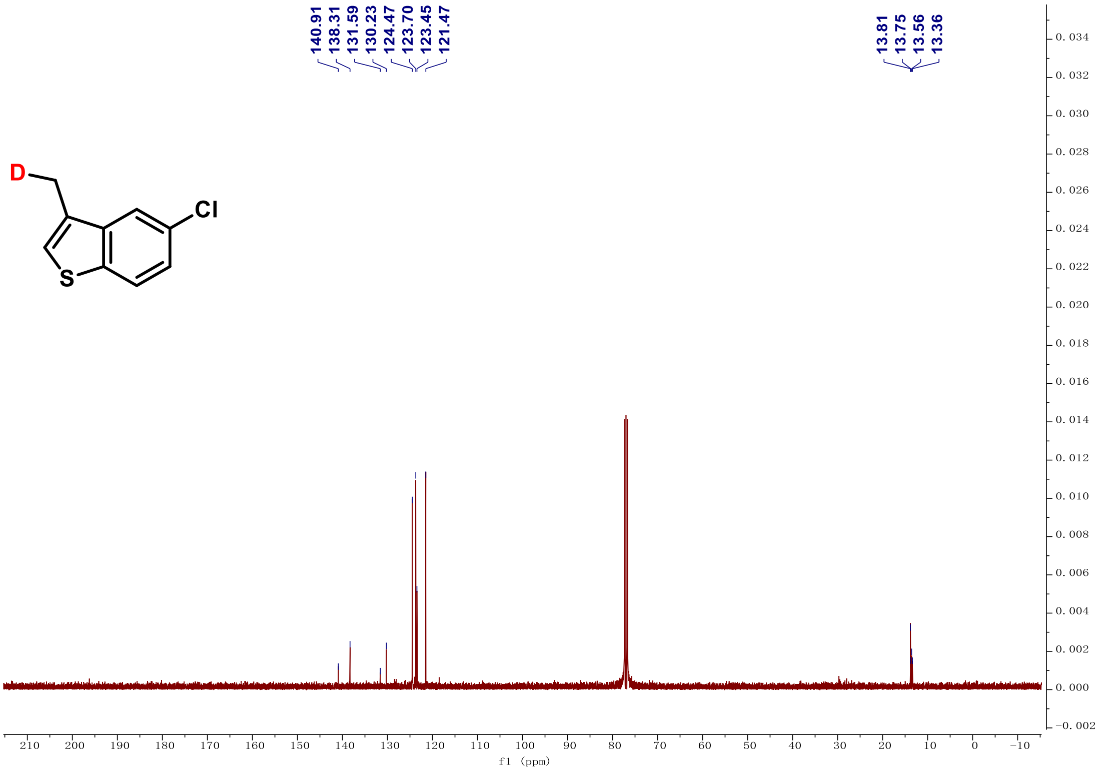


$^{13}$**C NMR** (100 MHz, $CDCl_3$) Spectrum of Compound **2aa**

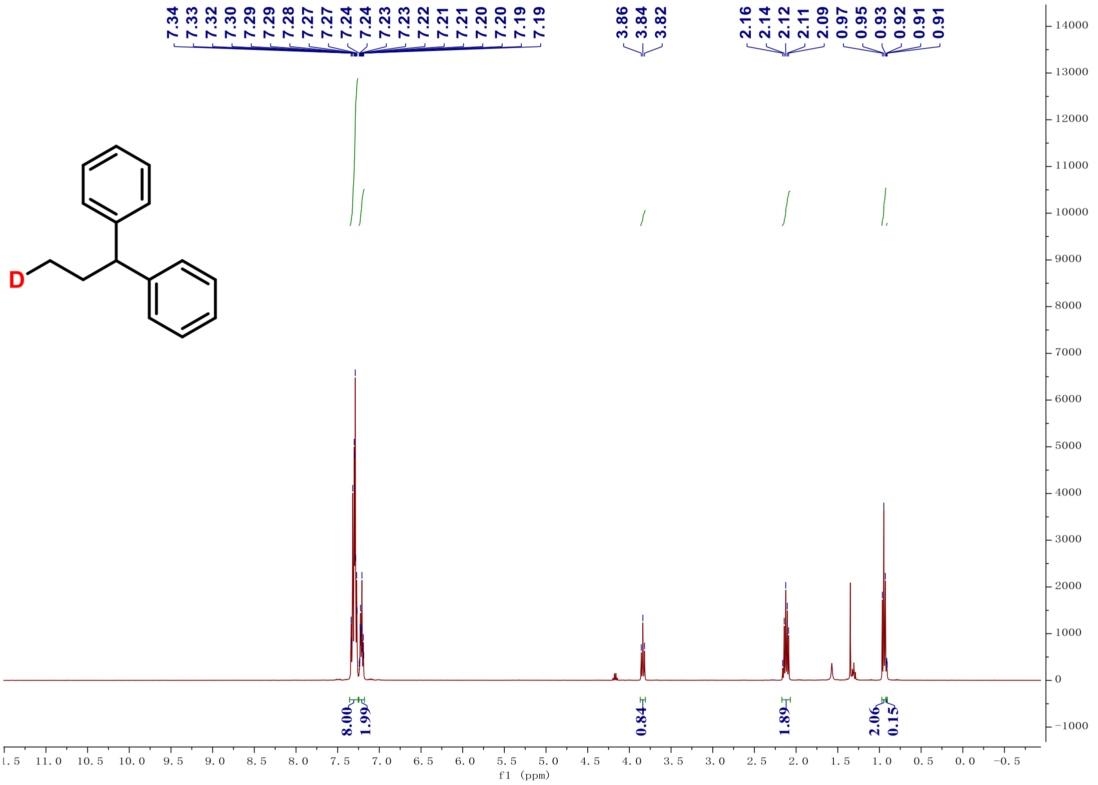


$^{1}$**H NMR** (400 MHz, $CDCl_3$) Spectrum of Compound **2ab**

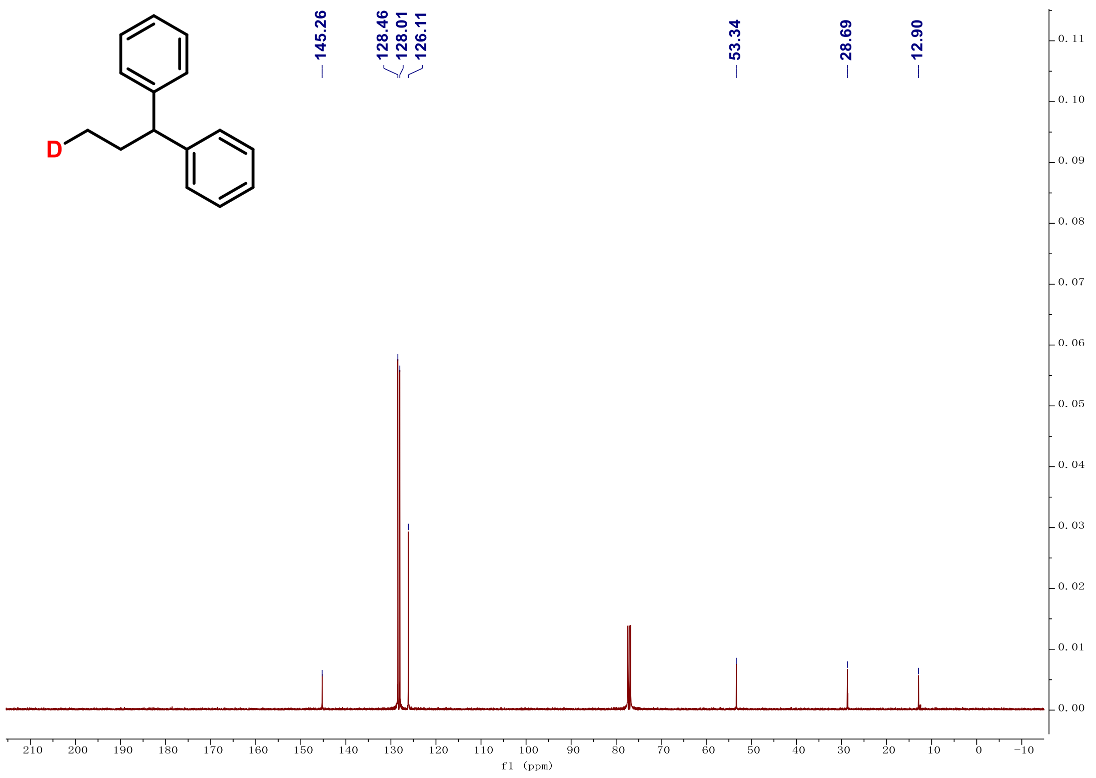


$^{13}$C **NMR** (100 MHz, $CDCl_3$) Spectrum of Compound **2ab**

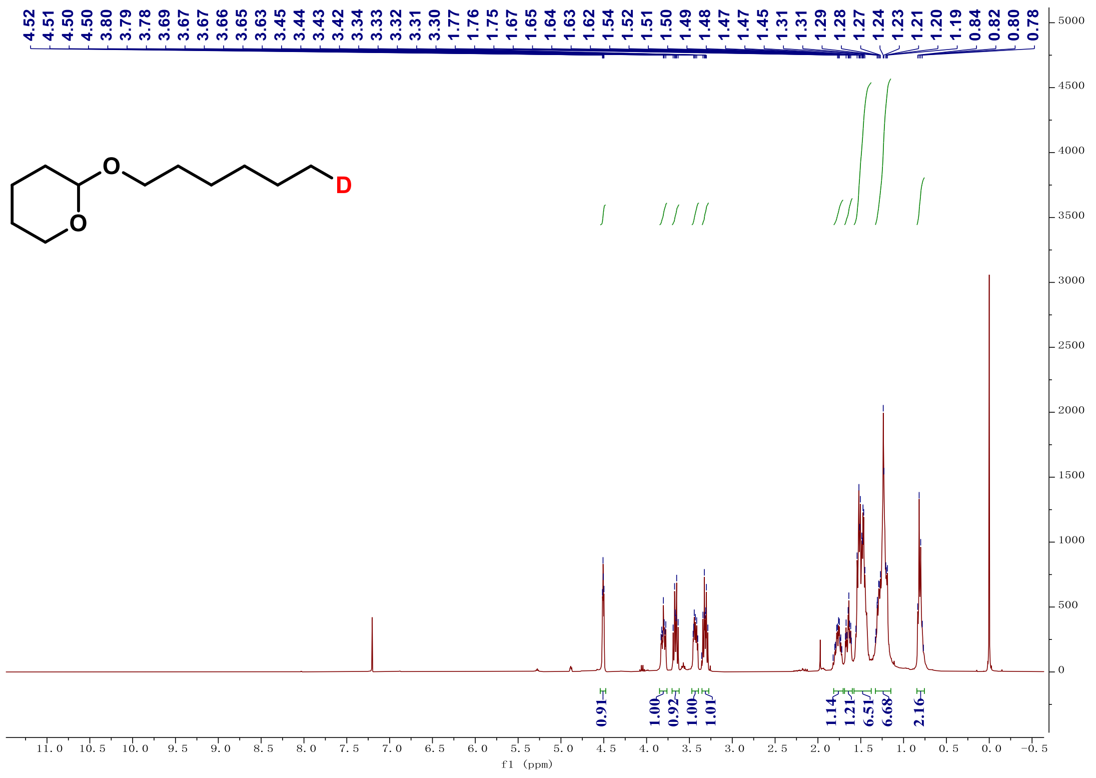


$^{1}$H **NMR** (400 MHz, $CDCl_3$) Spectrum of Compound **2ac**

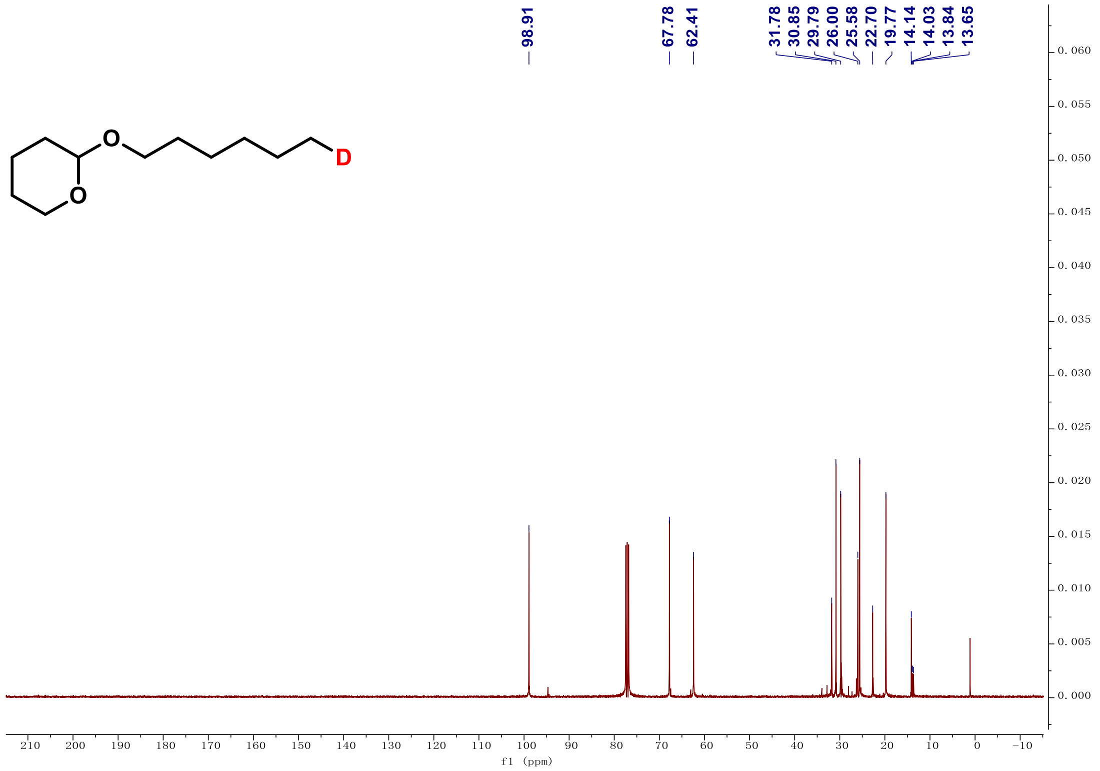


$^{13}$C NMR (100 MHz, $CDCl_3$) Spectrum of Compound **2ac**

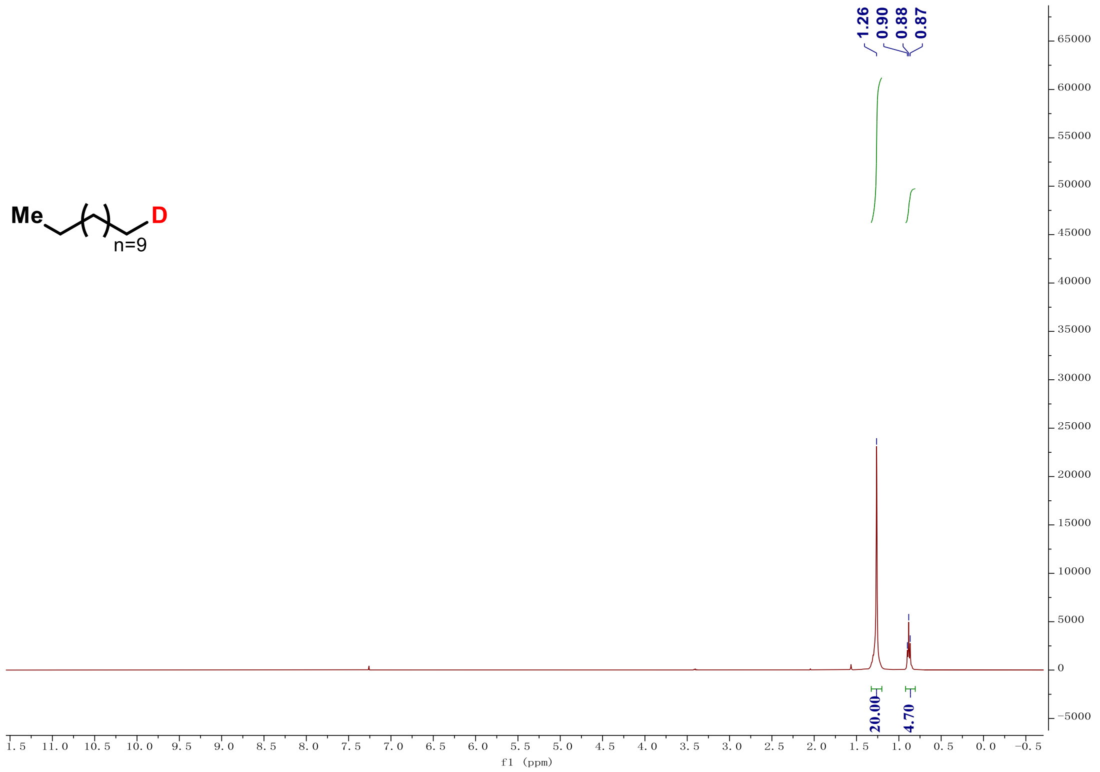


$^{1}$H NMR (400 MHz, $CDCl_3$) Spectrum of Compound **2ad**

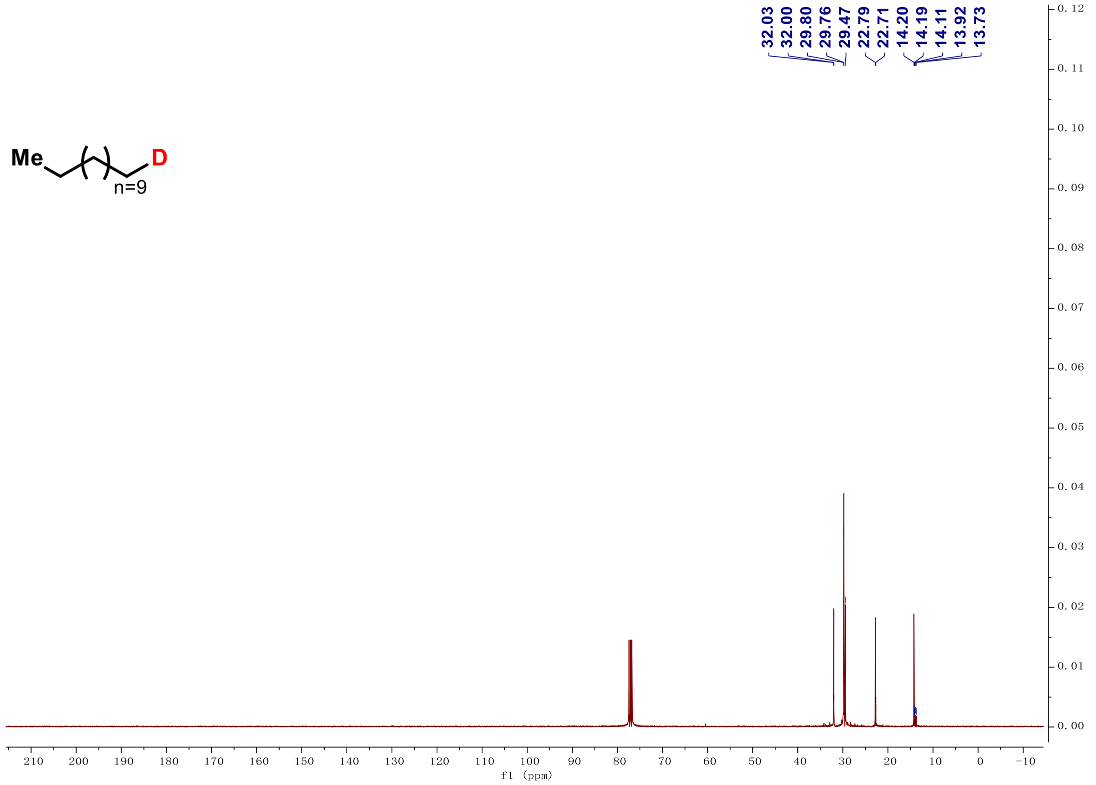


**$^{13}$C NMR** (100 MHz, $CDCl_3$) Spectrum of Compound **2ad**

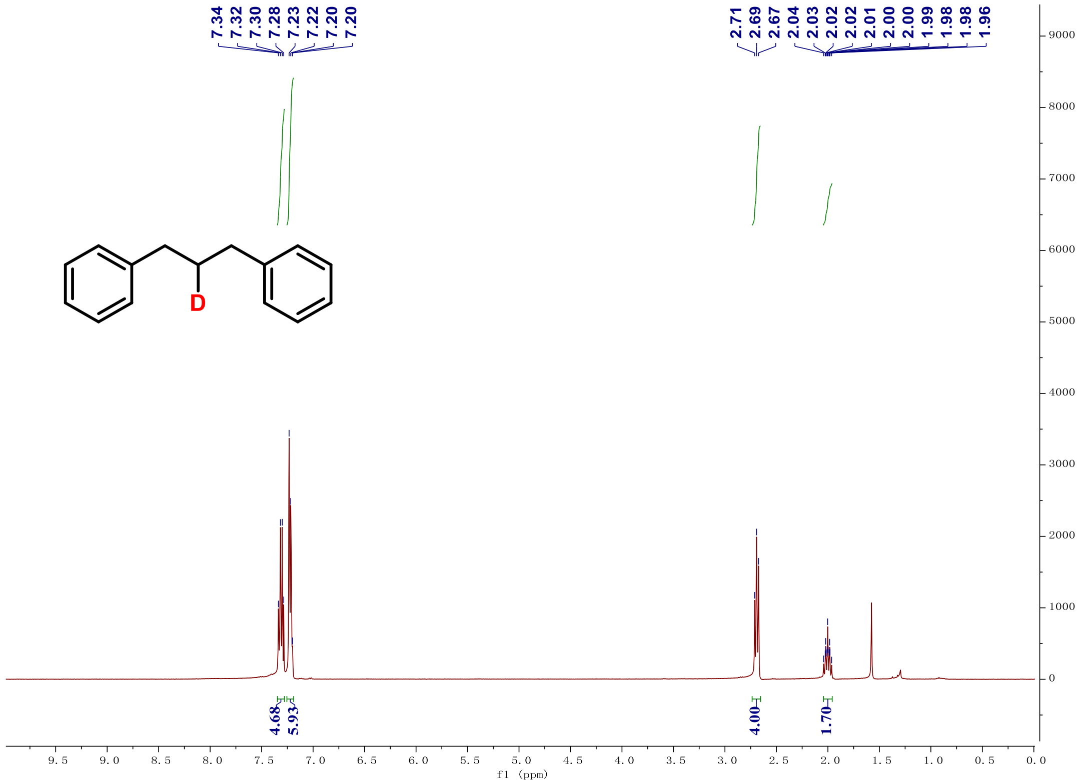


**$^{1}$H NMR** (400 MHz, $CDCl_3$) Spectrum of Compound **2ae**

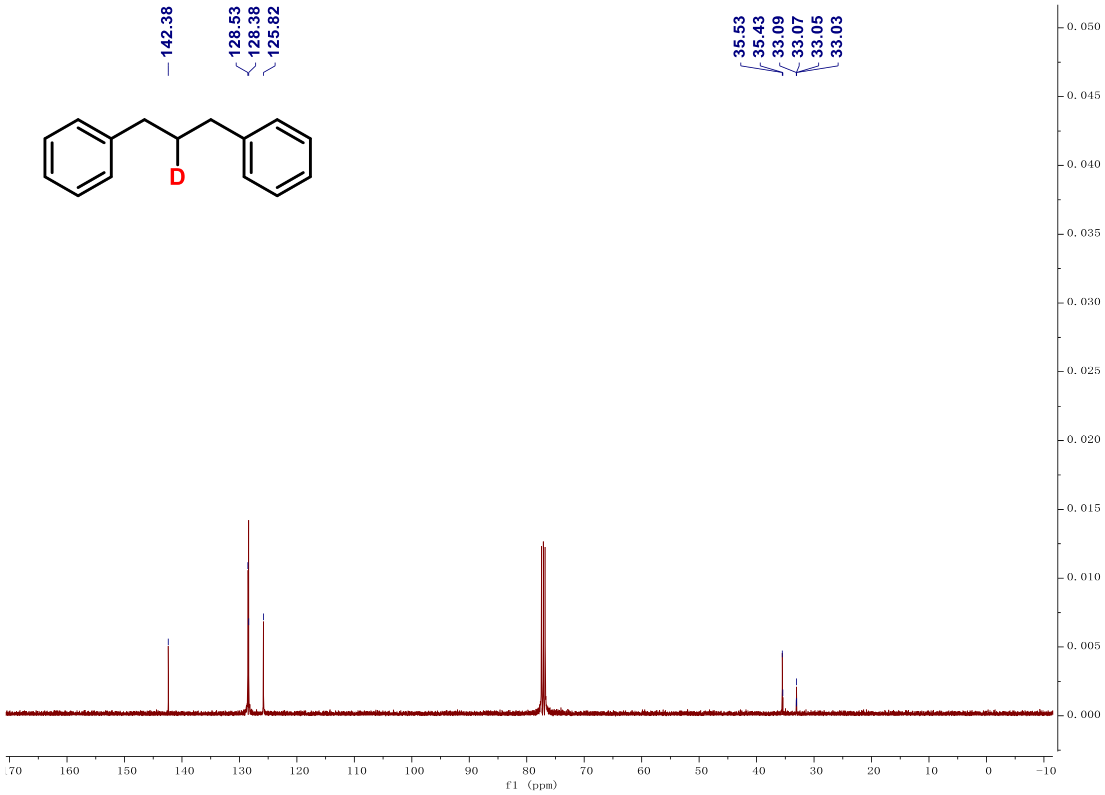


$^{13}$**C NMR** (100 MHz, $CDCl_3$) Spectrum of Compound **2ae**

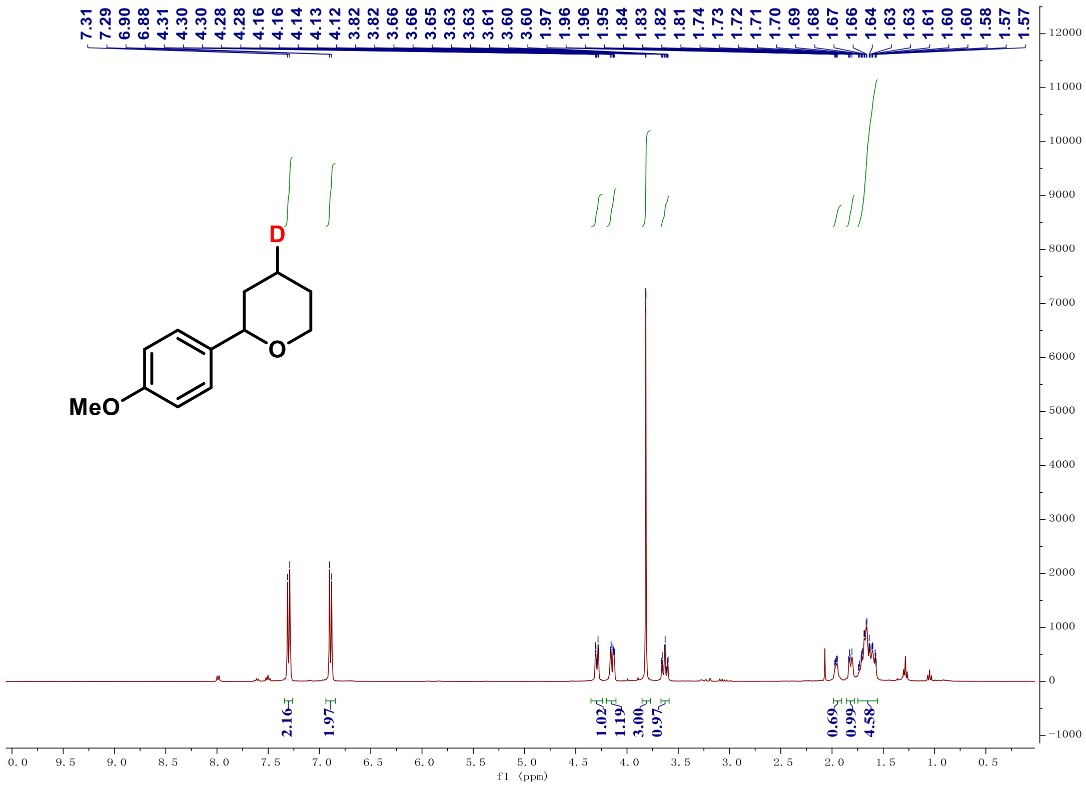


$^{1}$**H NMR** (400 MHz, $CDCl_3$) Spectrum of Compound **2af**

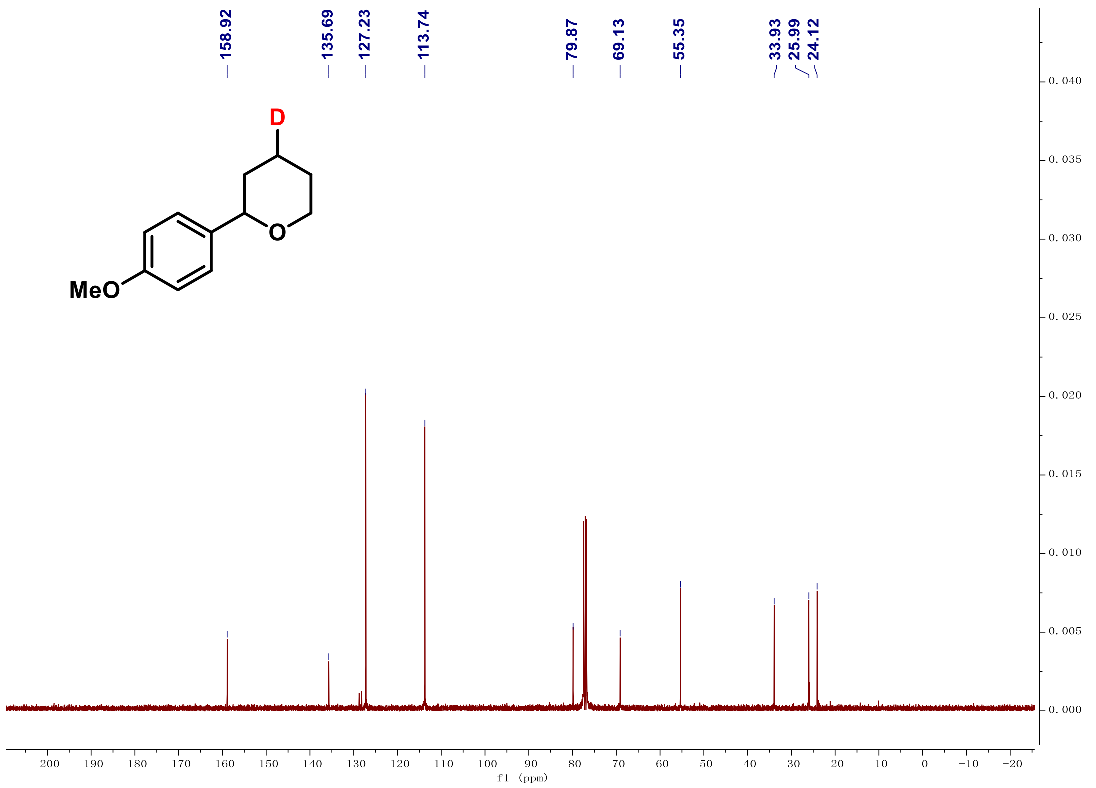


$^{13}$**C NMR** (100 MHz, $CDCl_3$) Spectrum of Compound **2af**

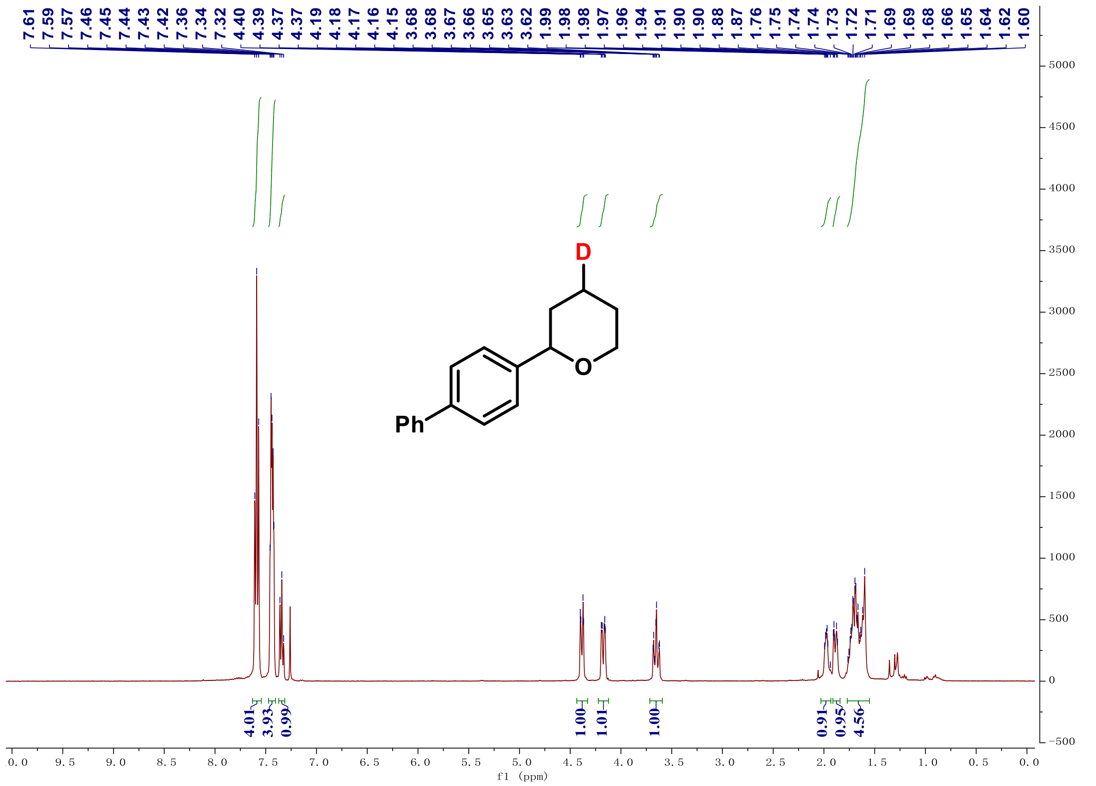


$^{1}$**H NMR** (400 MHz, $CDCl_3$) Spectrum of Compound **2ag**

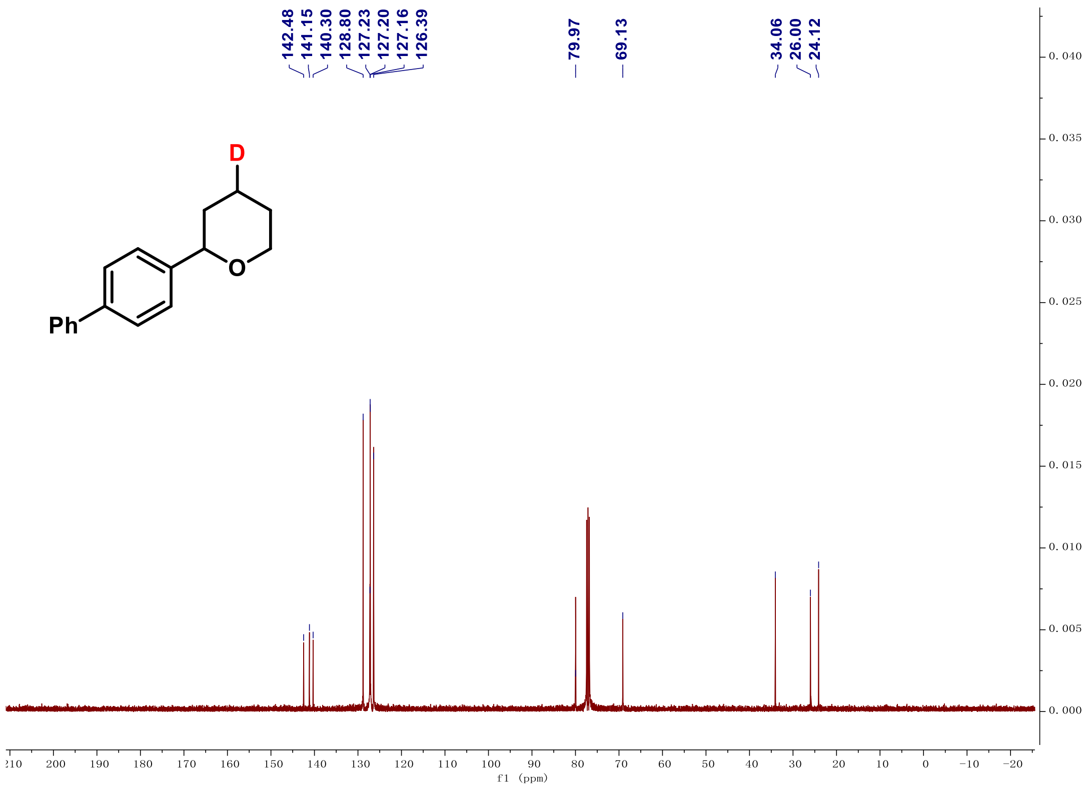


$^{13}$**C NMR** (100 MHz, $CDCl_3$) Spectrum of Compound **2ag**

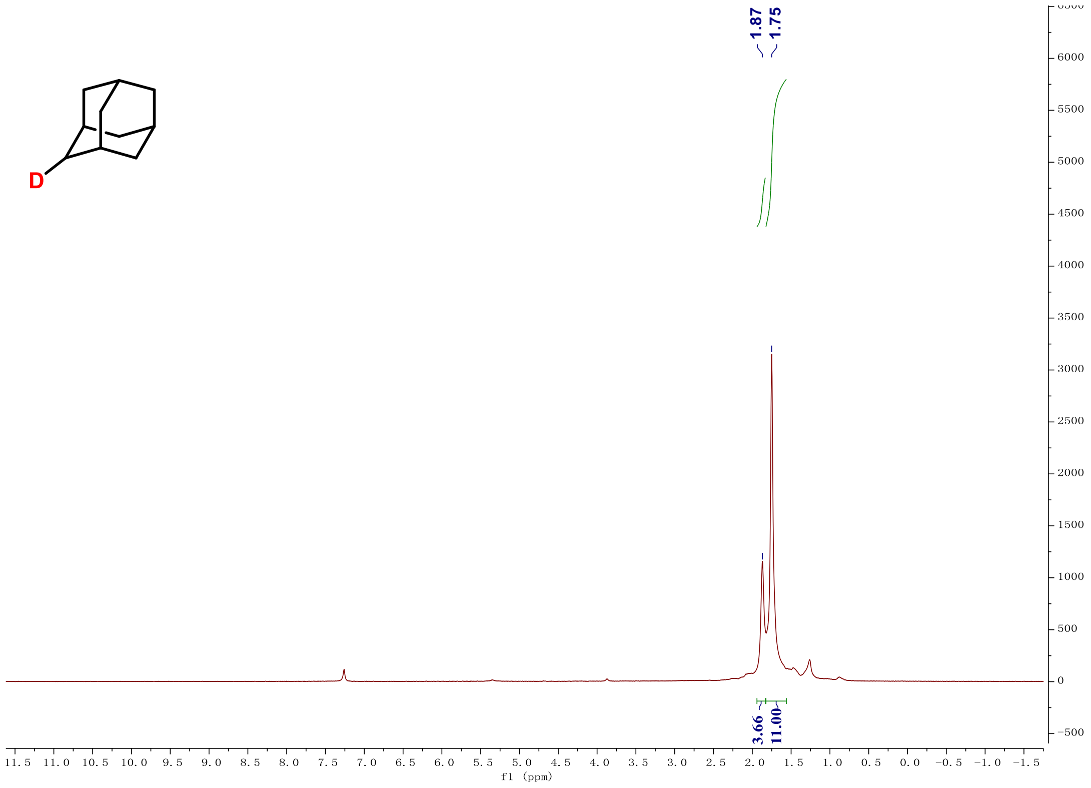


$^{1}$**H NMR** (400 MHz, $CDCl_3$) Spectrum of Compound **2ah**

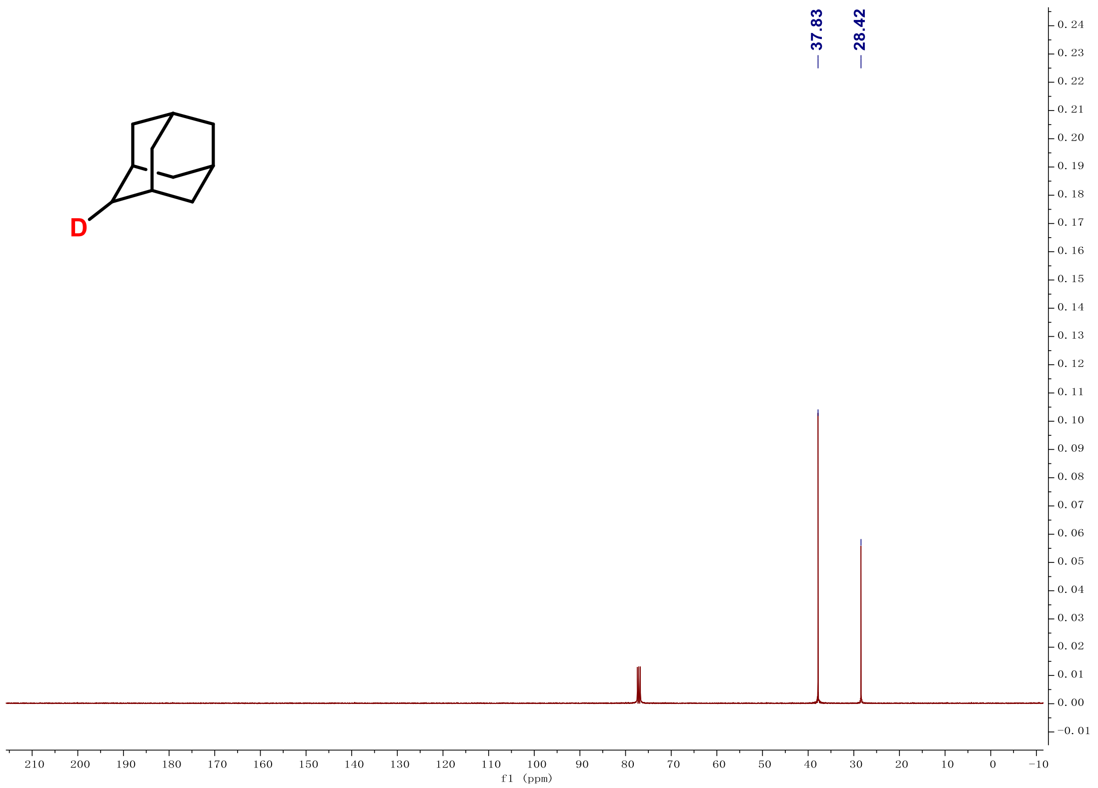


$^{13}$**C NMR** (100 MHz, $CDCl_3$) Spectrum of Compound **2ah**

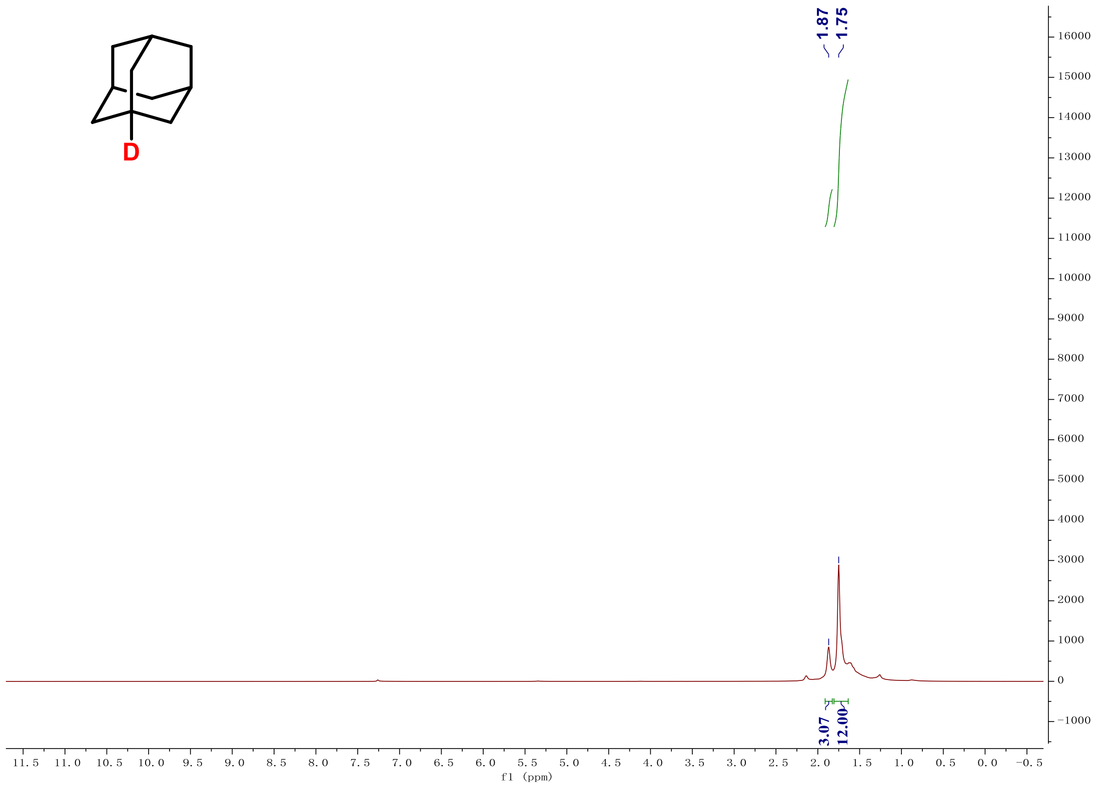


$^{1}$**H NMR** (400 MHz, $CDCl_3$) Spectrum of Compound **2ai**

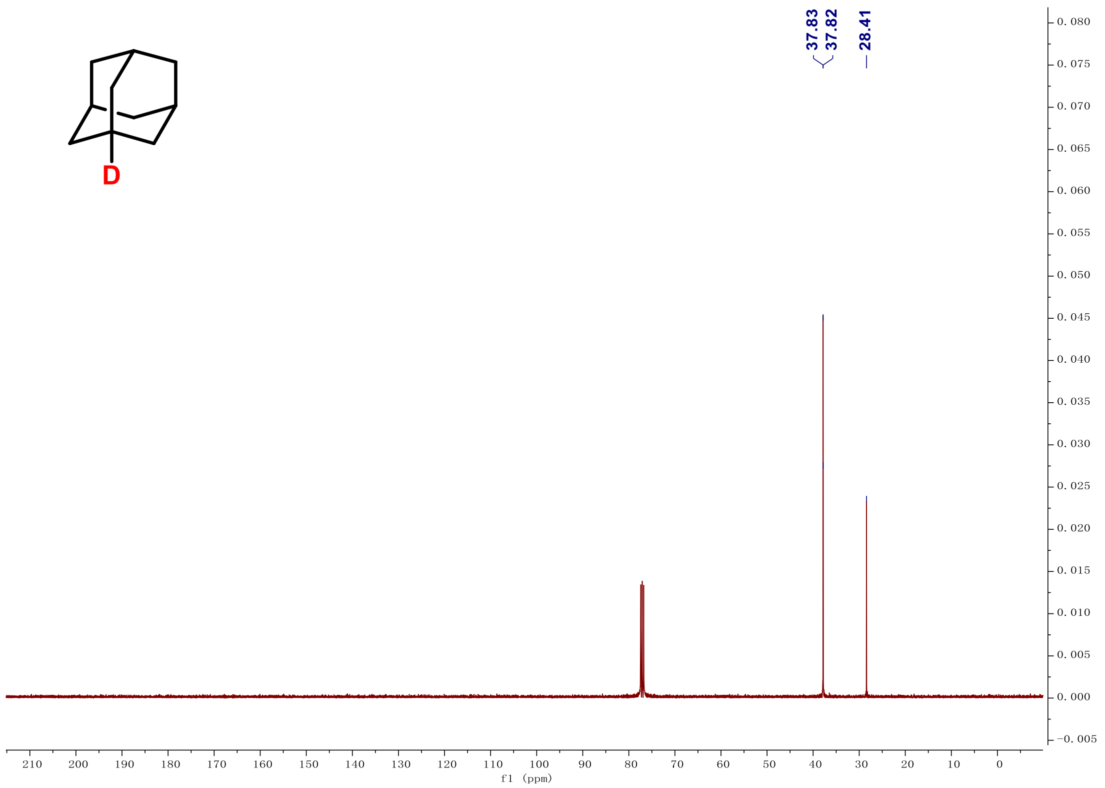


**$^{13}$C NMR** (100 MHz, $CDCl_3$) Spectrum of Compound **2ai**

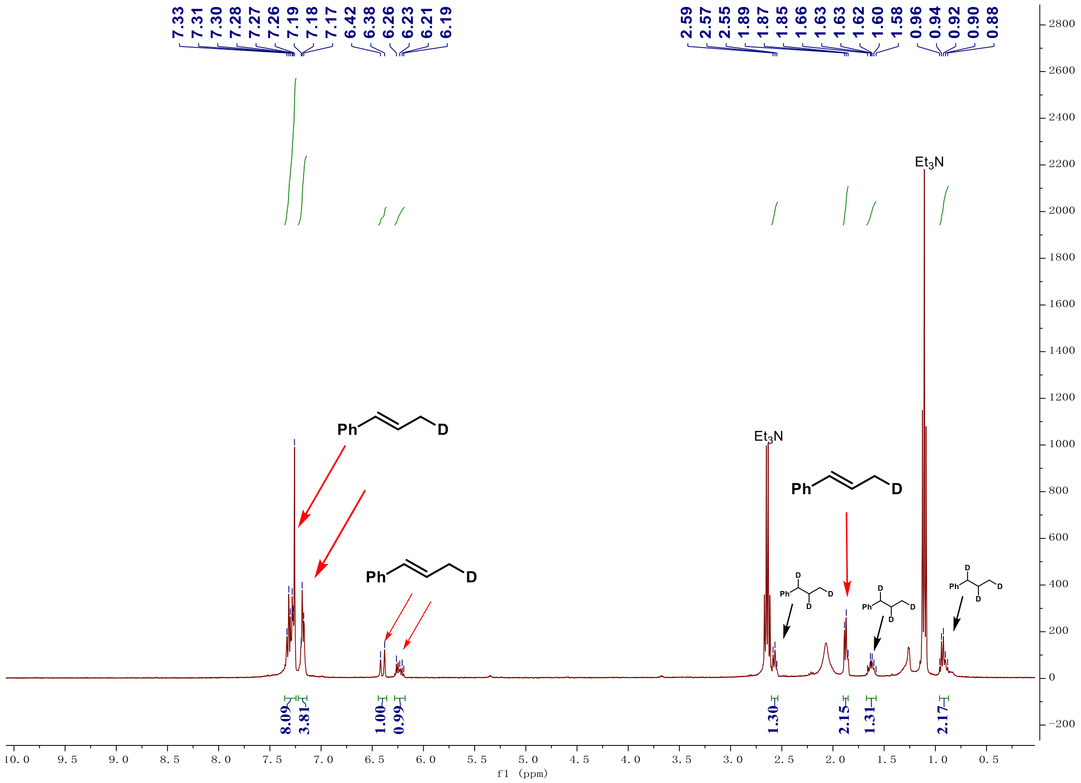


**$^{1}$H NMR (400 MHz, $CDCl_3$)** Spectrum of Compound **2aj** (crude mixture obtained after the reaction)

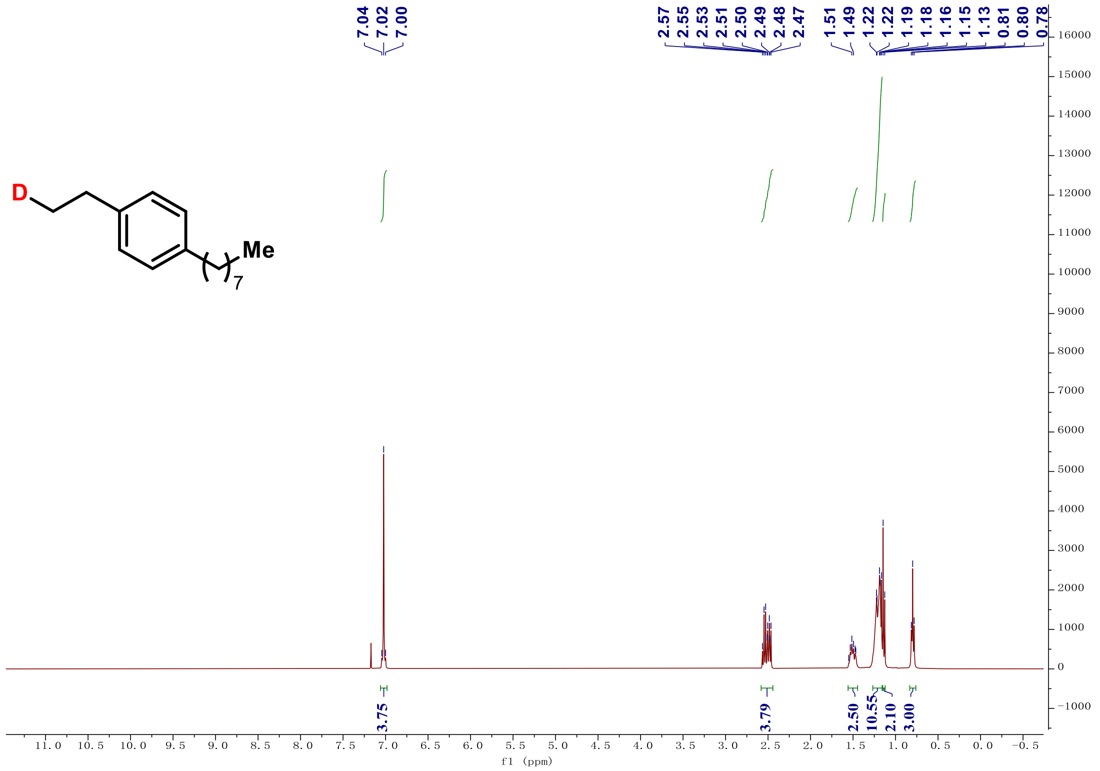


$^1$**H NMR** (400 MHz, $CDCl_3$) Spectrum of Compound **2ak**

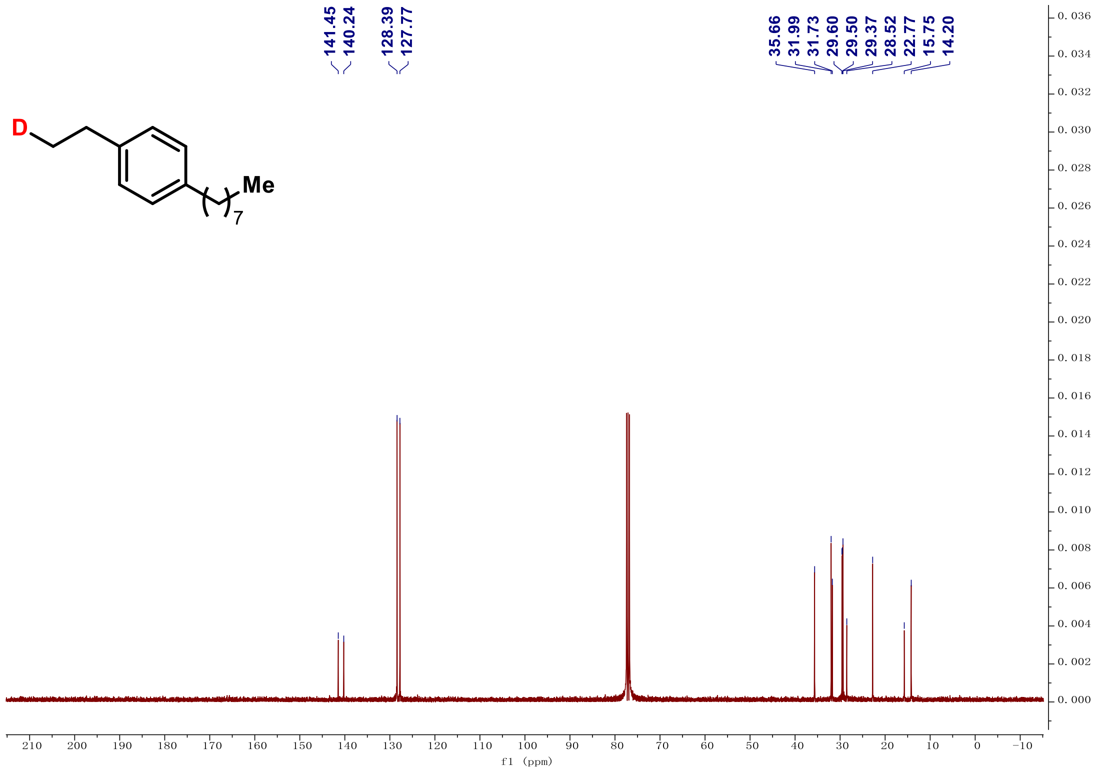


$^{13}$**C NMR** (100 MHz, $CDCl_3$) Spectrum of Compound **2ak**

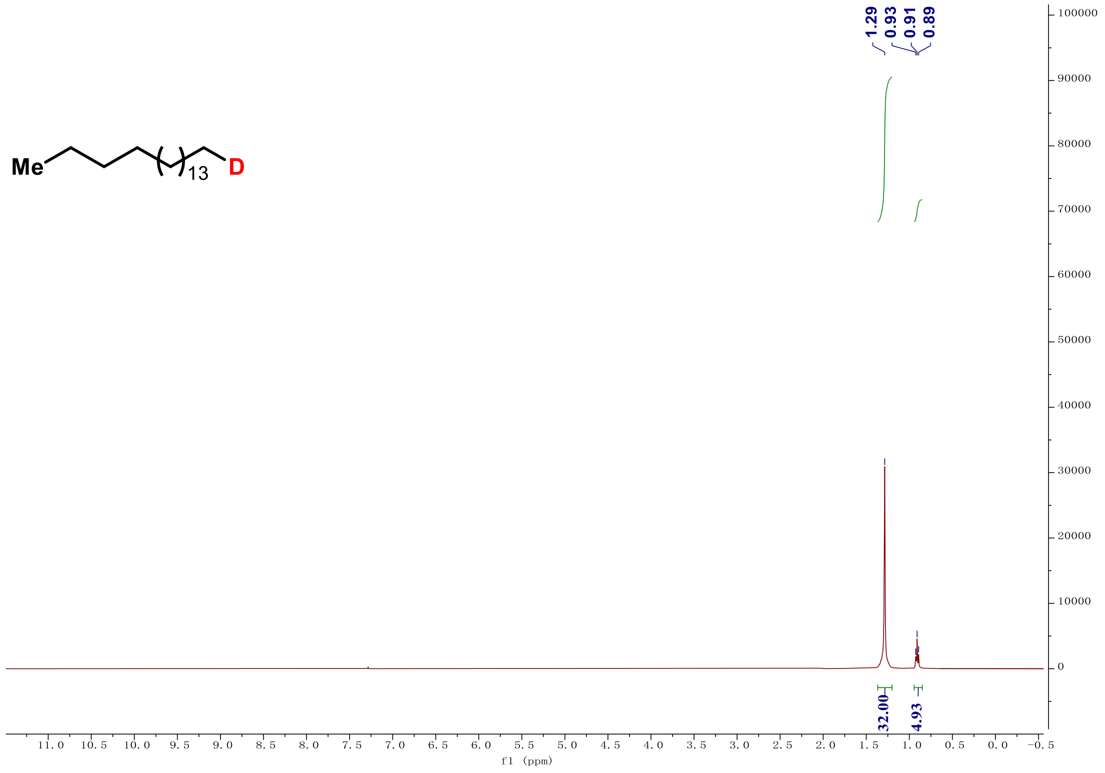


**$^{1}$H NMR** (400 MHz, DMSO-*d6*) Spectrum of Compound **2al**

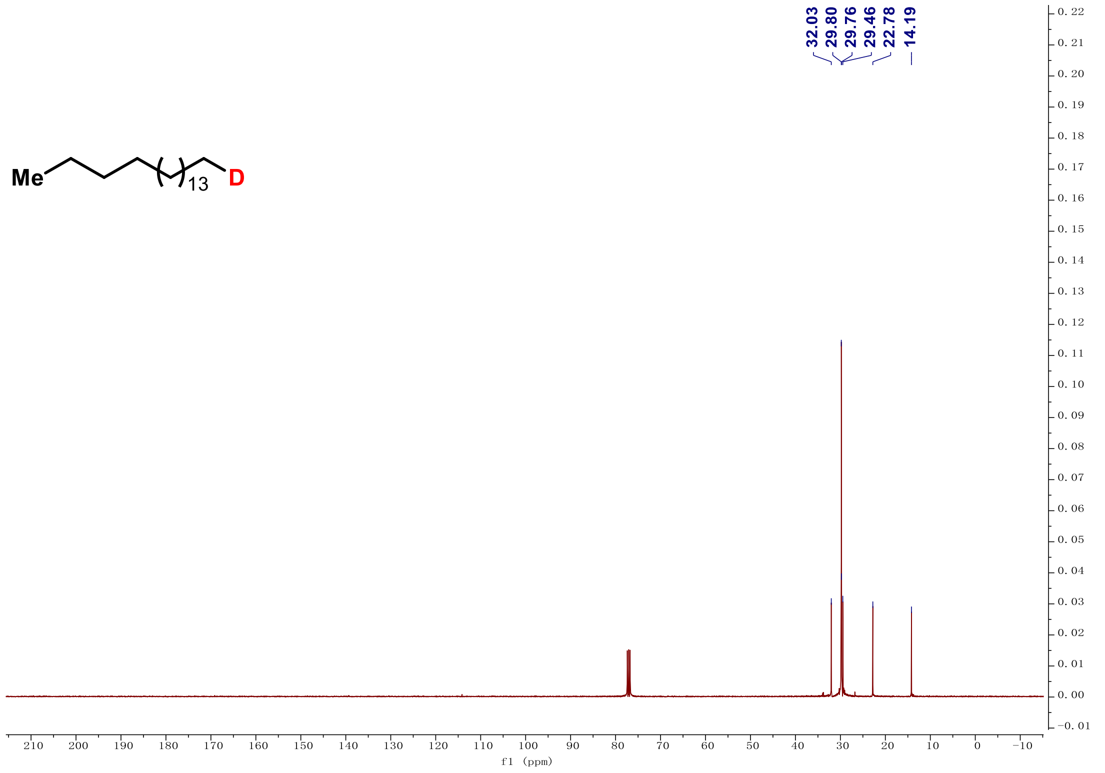


**$^{13}$C NMR** (100 MHz, $CDCl_3$) Spectrum of Compound **2al**

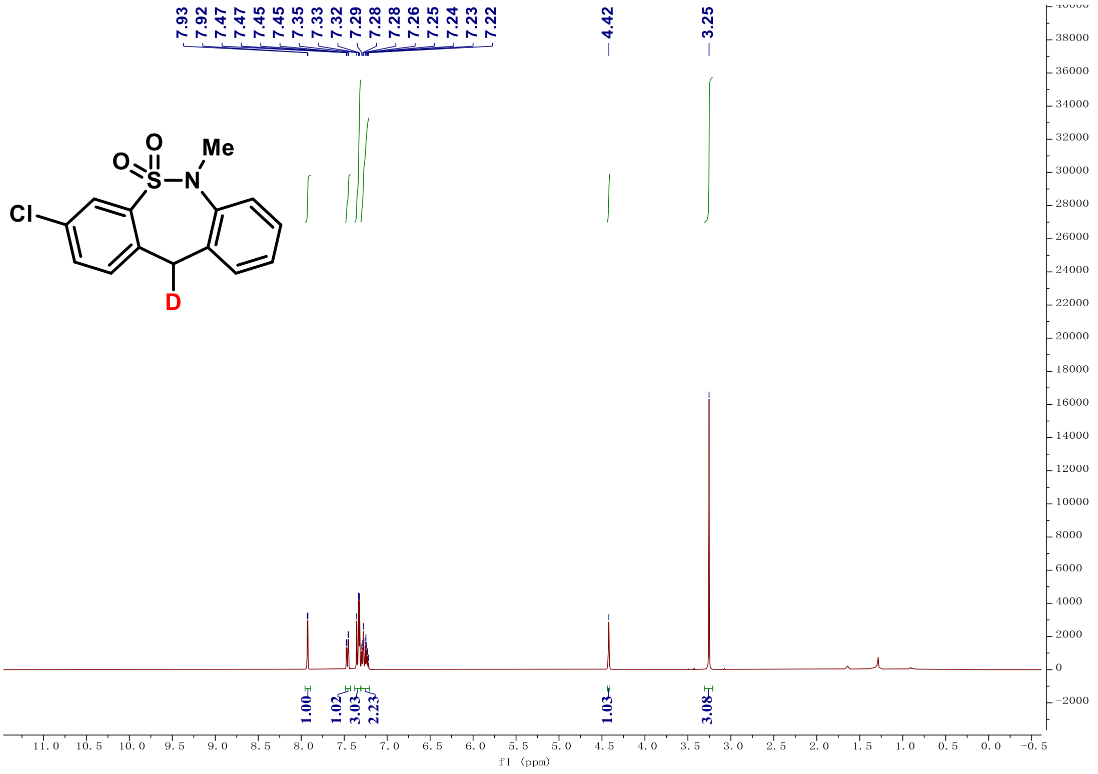


$^{1}$**H NMR** (400 MHz, $CDCl_3$) Spectrum of Compound **2am**

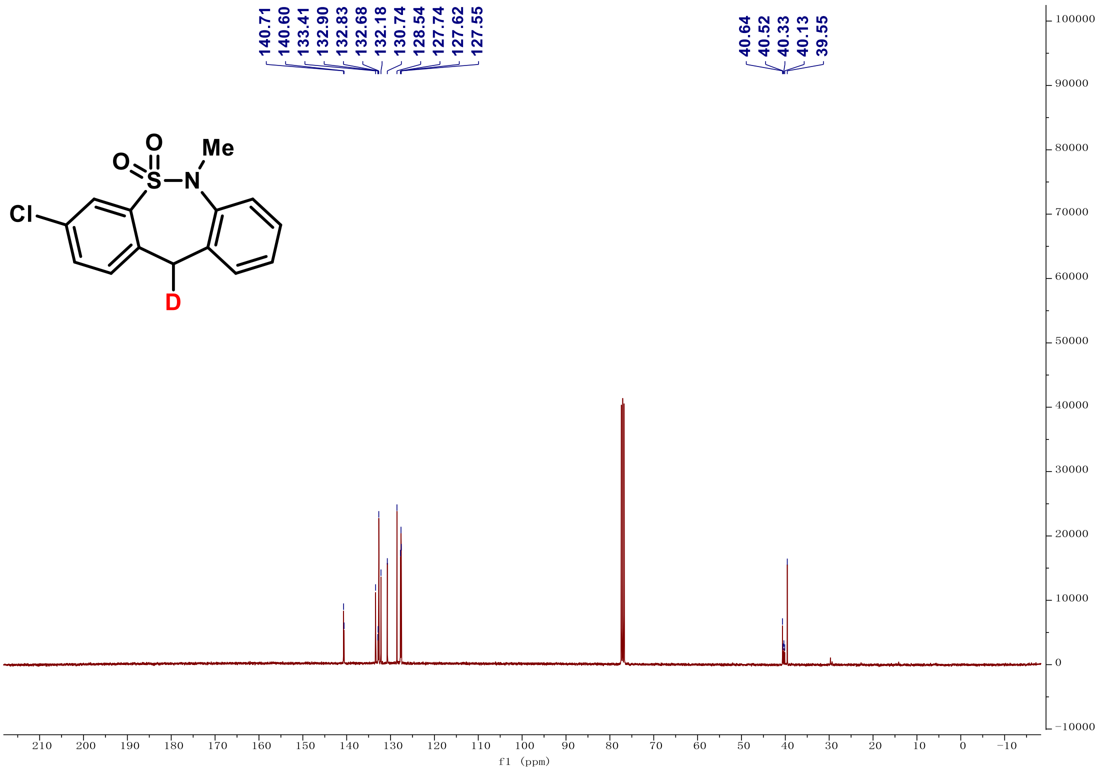


$^{13}$**C NMR** (100 MHz, $CDCl_3$) Spectrum of Compound **2am**

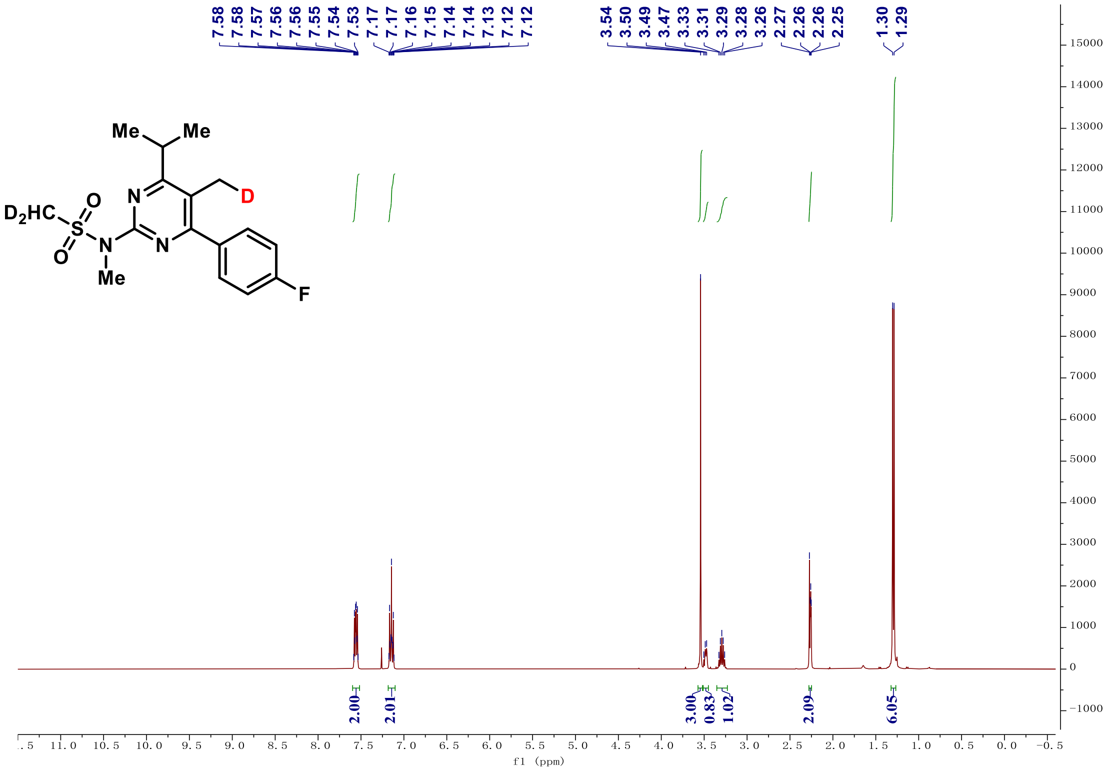


$^{1}$**H NMR** (400 MHz, $CDCl_3$) Spectrum of Compound **2an**

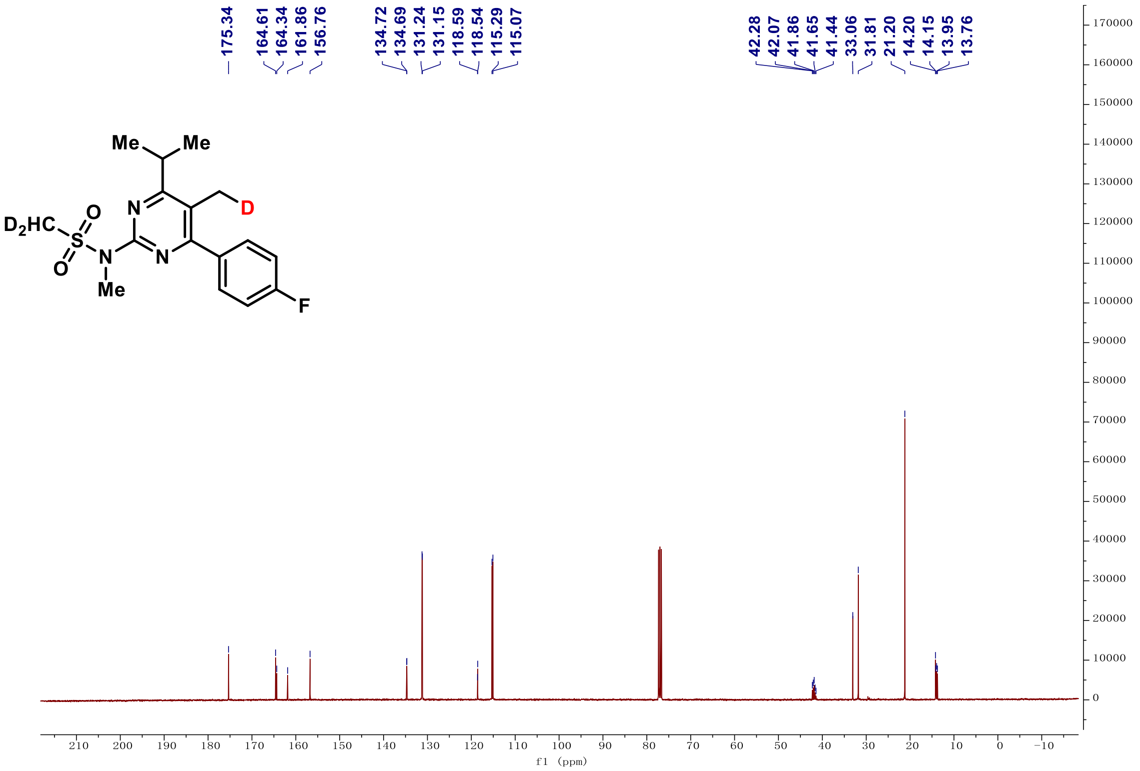


$^{13}$**C NMR** (100 MHz, $CDCl_3$) Spectrum of Compound **2an**

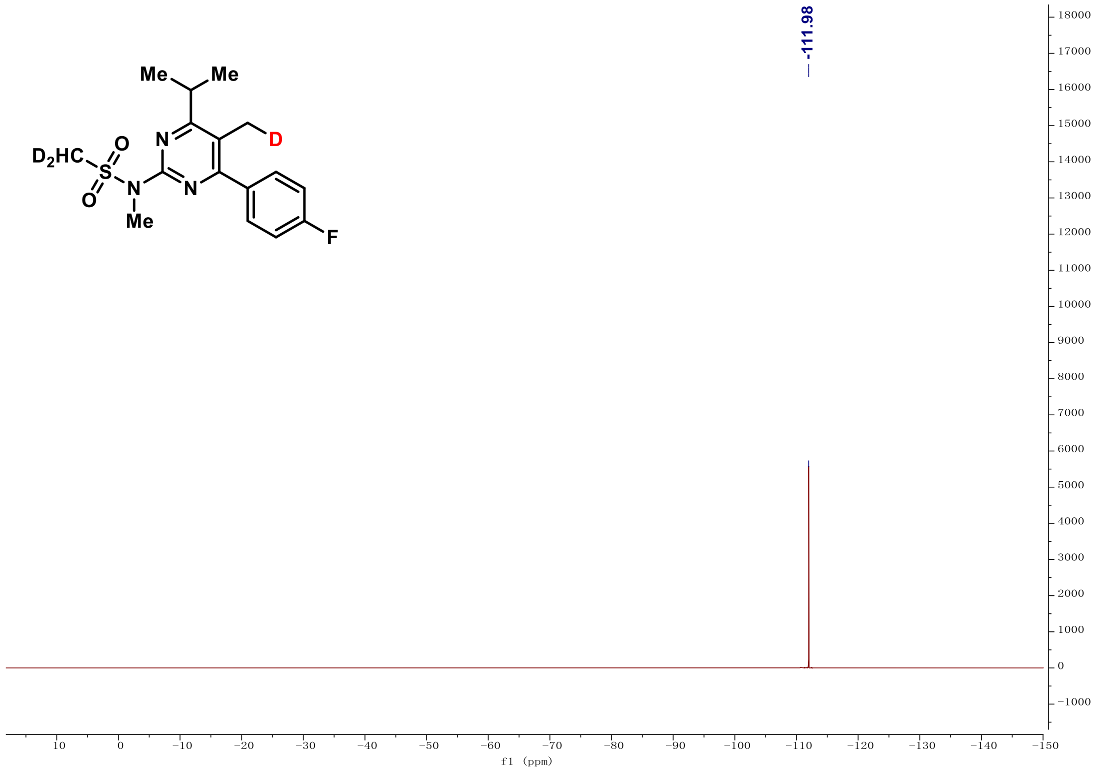

**$^{19}$F NMR** (376 MHz, $CDCl_3$) Spectrum of Compound **2an**

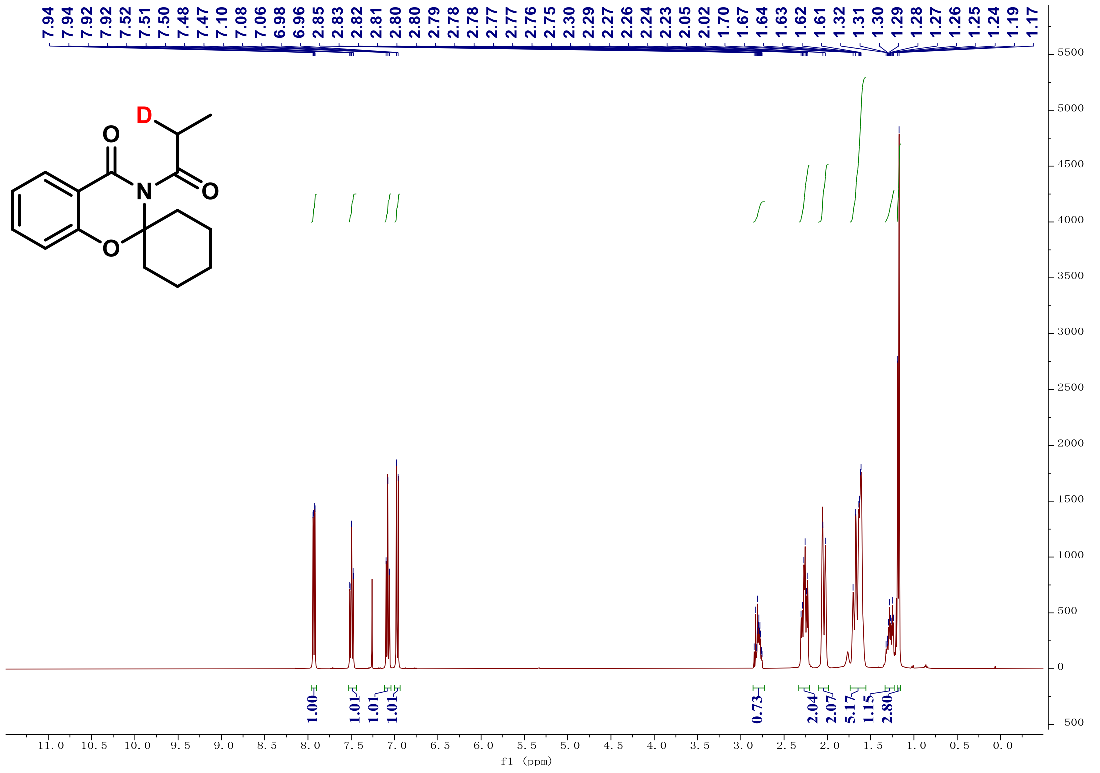

**$^{1}$H NMR** (400 MHz, $CDCl_3$) Spectrum of Compound **2ao**

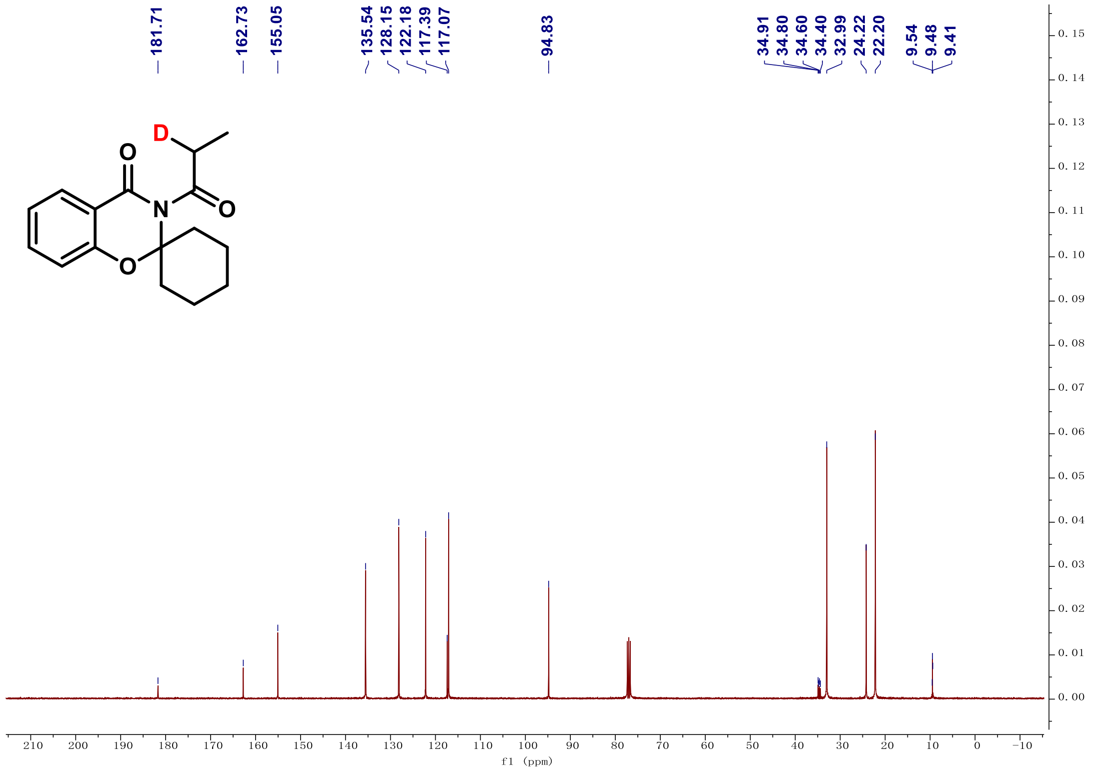


$^{13}$**C NMR** (100 MHz, $CDCl_3$) Spectrum of Compound **2ao**
